# Policies and interventions to remove gender‐related barriers to girls' school participation and learning in low‐ and middle‐income countries: A systematic review of the evidence

**DOI:** 10.1002/cl2.1207

**Published:** 2022-01-19

**Authors:** Stephanie Psaki, Nicole Haberland, Barbara Mensch, Lauren Woyczynski, Erica Chuang

**Affiliations:** ^1^ Population Council Washington District of Columbia USA; ^2^ Population Council New York New York USA

## Abstract

**Background:**

Gender disparities in education continue to undermine girls' opportunities, despite enormous strides in recent years to improve primary enrolment and attainment for girls in low‐ and middle‐income countries (LMICs). At the regional, country and subnational levels gender gaps remain, with girls in many settings less likely to complete primary school, less likely to complete secondary, and often less likely to be literate than boys. The academic and policy literatures on the topic of gender‐related barriers to girls' education are both extensive. However, there remain gaps in knowledge regarding which interventions are most likely to work in contexts with different combinations of barriers.

**Objectives:**

This systematic review identified and assessed the strength of the evidence of interventions and exposures addressing gender‐related barriers to schooling for girls in LMICs.

**Search Methods:**

The AEA RCT Registry, Africa Bibliography, African Education Research Database, African Journals Online, DEC USAID, Dissertation Abstracts, EconLit, ELDIS, Evidence Hub, Global Index Medicus, IDEAS‐Repec, Intl Clinical Trials Registry, NBER, OpenGrey, Open Knowledge Repository, POPLINE, PsychINFO, PubMed, Research for Development Outputs, ScienceDirect, Sociological Abstracts, Web of Science, as well as relevant organization websites were searched electronically in March and April of 2019. Further searches were conducted through review of bibliographies as well as through inquiries to authors of included studies, relevant researchers and relevant organizations, and completed in March 2020.

**Selection Criteria:**

We included randomized controlled trials as well as quasi‐experimental studies that used quantitative models that attempted to control for endogeneity. Manuscripts could be either published, peer‐reviewed articles or grey literature such as working papers, reports and dissertations. Studies must have been published on or after 2000, employed an intervention or exposure that attempted to address a gender‐related barrier to schooling, analyzed the effects of the intervention/exposure on at least one of our primary outcomes of interest, and utilized data from LMICs to be included.

**Data Collection and Analysis:**

A team of reviewers was grouped into pairs to independently screen articles for relevance, extract data and assess risk of bias for each included study. A third reviewer assisted in resolving any disputes. Risk of bias was assessed either through the RoB 2 tool for experimental studies or the ROBINS‐I tool for quasi‐experimental studies. Due to the heterogeneity of study characteristics and reported outcome measures between studies, we applied the GRADE (Grading of Recommendation, Assessment, Development and Evaluation) approach adapted for situations where a meta‐analysis is not possible to synthesize the research.

**Results:**

Interventions rated as effective exist for three gender‐related barriers: inability to afford tuition and fees, lack of adequate food, and insufficient academic support. Promising interventions exist for three gender‐related barriers: inadequate school access, inability to afford school materials, and lack of water and sanitation. More research is needed for the remaining 12 gender‐related barriers: lack of support for girls' education, child marriage and adolescent pregnancy, lack of information on returns to education/alternative roles for women, school‐related gender‐based violence (SRGBV), lack of safe spaces and social connections, inadequate sports programs for girls, inadequate health and childcare services, inadequate life skills, inadequate menstrual hygiene management (MHM), poor policy/legal environment, lack of teaching materials and supplies, and gender‐insensitive school environment. We find substantial gaps in the evidence. Several gender‐related barriers to girls' schooling are under‐examined. For nine of these barriers we found fewer than 10 relevant evaluations, and for five of the barriers—child marriage and adolescent pregnancy, SRGBV, inadequate sports programs for girls, inadequate health and childcare services, and inadequate MHM—we found fewer than five relevant evaluations; thus, more research is needed to understand the most effective interventions to address many of those barriers. Also, nearly half of programs evaluated in the included studies were multi‐component, and most evaluations were not designed to tease out the effects of individual components. As a result, even when interventions were effective overall, it is often difficult to identify how much, if any, of the impact is attributable to a given program component. The combination of components varies between studies, with few comparable interventions, further limiting our ability to identify packages of interventions that work well. Finally, the context‐specific nature of these barriers—whether a barrier exists in a setting and how it manifests and operates—means that a program that is effective in one setting may not be effective in another.

**Authors' Conclusions:**

While some effective and promising approaches exist to address gender‐related barriers to education for girls, evidence gaps exist on more than half of our hypothesized gender‐related barriers to education, including lack of support for girls' education, SRGBV, lack of safe spaces and social connections, inadequate life skills, and inadequate MHM, among others. In some cases, despite numerous studies examining interventions addressing a specific barrier, studies either did not disaggregate results by sex, or they were not designed to isolate the effects of each intervention component. Differences in context and in implementation, such as the number of program components, curricula content, and duration of interventions, also make it difficult to compare interventions to one another. Finally, few studies looked at pathways between interventions and education outcomes, so the reasons for differences in outcomes largely remain unclear.

## 1 PLAIN LANGUAGE SUMMARY

### 1.1 Some interventions may improve girls' education performance, but there is insufficient evidence on others

Gender disparities in education persist in many countries. Interventions that address financial barriers to school, such as inability to afford tuition and fees and lack of adequate food, as well as those that address insufficient academic support, may be effective at improving girls' education outcomes. Interventions to increase access to schools, to provide school materials, and to improve water and sanitation in schools, especially toilets, are promising approaches for girls as well. However, for many gender‐related barriers to education, the dearth of evaluations and lack of clarity about pathways through which they operate makes it difficult to determine whether many common interventions are effective.

### 1.2 What is this review about?

Gender disparities in education persist in many low‐ and middle‐income countries. A clear understanding of the most effective approaches to improving education outcomes for girls, and to narrowing gender gaps, is largely missing from literature and practice. This review looks at whether interventions that address gender‐related barriers to girls' education help improve education outcomes for girls, specifically attainment, enrolment, absenteeism and academic performance.

**What is the aim of this review?**

This systematic review summarises evidence from 82 experimental and quasi‐experimental studies from low‐ and middle‐income countries to assess what programmes may help to improve girls' education outcomes and narrow gender disparities.

### 1.3 What studies are included?

This review includes 82 experimental and quasi‐experimental studies of interventions that address at least one gender‐related barrier to schooling and measure impact on girls' education outcomes. Study locations spanned all regions of the developing world. Both peer‐reviewed journal articles and grey literature were included, with publication dates from 2004 to 2020.

### 1.4 What are the main findings of this review?

We identified no evaluations of the effects of school‐related gender‐based violence interventions on girls' education. Also, too few studies examined sports programmes for girls, school‐based health and childcare, child marriage and adolescent pregnancy, and menstrual hygiene management to draw strong conclusions.

Interventions that address financial barriers to school (such as inability to afford tuition and fees and lack of adequate food) as well as those that address insufficient academic support, may be effective at improving girls' education outcomes. Interventions that aim to improve girls' access to schools and materials, and improve water and sanitation in schools, especially toilets, are also promising approaches.

For interventions addressing the remaining gender‐related barriers to school, existing evidence was inconclusive, though some programmes were effective in some settings. In many cases, interventions included multiple components, and studies were often not designed to test the effects of each individual component.

### 1.5 What do the findings of this review mean?

Although some clear findings emerged in terms of promising practices, evidence gaps exist for the majority of gender‐related barriers to education for girls, particularly for school‐related gender‐based violence, lack of sports programmes, and lack of health and childcare services.

The majority of included studies evaluate complex multi‐component programmes, rather than narrowly defined single component programmes, and few employ a factorial design, often making it difficult to determine which components are most important for driving improvements in education outcomes for girls.

Differences in implementation also make it difficult to compare interventions. Because the importance of each gender‐related barrier varies between settings, findings from existing research may not be relevant to all settings. Too few studies disaggregate results by sex, representing a missed opportunity to close gaps in evidence.

Finally, the dearth of studies looking at pathways linking the interventions to education outcomes makes it difficult to determine why certain interventions “worked” in some settings and not in others.

### 1.6 How up to date is this review?

The review authors searched for studies up to March 2020.

## 2 BACKGROUND

### 2.1 The problem: Gender inequality in education and poor outcomes for girls

While enormous progress has been made in recent years in increasing girls' primary school enrolment and attainment, gender disparities remain in many low‐ and middle‐ income countries (LMICs) (Evans et al., 2021; Psaki et al., 2018; Wodon et al., 2020). An examination of trends between 1997–2007 and 2008–2014 revealed that girls' educational attainment has stagnated; of the 43 countries in the sample, only three made substantial progress in both female attainment and reducing the gender gap in attainment (Psaki et al., 2018). A more recent analysis of data from 126 countries found that a gender gap remains in 90 countries (Evans et al., 2021). Moreover, higher grade attainment does not necessarily translate into improved learning; in an analysis of 23 countries with data on literacy, in nine countries less than 50% of girls were literate after completing primary school, and in seventeen countries boys were more likely than girls to have acquired basic literacy skills after completing primary school (Psaki et al., 2018).

While an extensive literature exists on the topic of gender‐related barriers to schooling, gaps in knowledge exist regarding the degree to which interventions to reduce gender‐related barriers to schooling in LMICs are effective in improving education outcomes for girls. This systematic review explores this question, adding an important perspective to complement reviews of education generally (Snilstveit et al., 2015), girls' education (Unterhalter et al., 2014), and specific approaches, such as water and sanitation (Jasper et al., 2012). We begin with a summary of the existing literature on gender‐related barriers to schooling. The set of perceived gender‐related barriers included below emerged from that literature review, as well as consultations with our advisory board. Through the process of conducting this review we adjusted the list of barriers (adding two) based on important distinctions between barriers identified through included studies. Such barriers include (a) factors that only or overwhelmingly affect girls—such as child marriage and gender norms which hold that girls' education is less valuable than boys'; (b) barriers that affect both girls and boys but because of intersections with inequitable gender norms and inequality, often affect girls more—such as lack of access to school and inability to pay tuition; as well as (c) barriers that are shared by both girls and boys but may differ in terms of import and the pathways through which they undermine education outcomes—such as pedagogy and lack of teaching materials and supplies. Underlying issues such as gender norms, and policy and legal environments, cut across these barriers. While the following barriers are not exhaustive, they are among the most frequently described. Some are prevalent across LMIC, but because these barriers are inherently shaped by social and cultural context, their influence—both the pervasiveness and how they manifest—varies from one setting to another. Note that, in the brief literature summaries below, we exclude results from studies included in our systematic review.

#### 2.1.1 Lack of support for girls' education (Barrier 1)

Community norms and parental attitudes about innate abilities of and appropriate roles for girls may undermine schooling. Norms may discourage girls themselves from learning, affect academic performance and lead to premature school dropout (Eble & Hu, 2019; Warrington & Kiragu, 2012). At the same time, when financial resources are limited, parents with inequitable gender role attitudes may prefer to keep sons in school rather than daughters (Lloyd & Young, 2009). Normative beliefs may thus affect both parents' decisions about whether, and how long, to send their daughters to school, as well as girls' own academic aspirations, school performance and behaviours (Global Education Monitoring Report Team, 2018). Indeed, where inequitable gender roles are entrenched, the incentives for girls to attend, and perform well in, school may be lower than those for boys (Colclough et al., 2000). This may be the case particularly where overall levels of education are low, with the result that in settings that perform poorly on other dimensions of development, gender gaps in attainment are largest (Evans et al., 2021; Global Education Monitoring Report Team, 2015; Psaki et al., 2018). While numerous studies have established that girls in some LMICs perform more household chores than their brothers (e.g., Amin & Chandrasekhar, 2012; Singh & Mukherjee, 2017) the effect of gender differences in time use on educational outcomes, which has been examined with observational data, is less clear (Hedges et al., 2018; Rees, 2017).

#### 2.1.2 Child marriage and adolescent pregnancy (Barrier 2)

Norms around age at marriage also intersect with education. One widely discussed potential consequence of child marriage is school dropout. A recent analysis that modelled the effect of successful interventions to reduce early marriage on education outcomes estimated “substantial” increases in secondary school completion (Rasmussen et al., 2019). It has been asserted that “the timing of early marriage almost always disrupts girls' education” (Mathur et al., 2003). Cross sectional analyses and qualitative studies have documented strong associations between early marriage/marital aspirations and school dropout in settings where marriage before age 18 is common (Prakash et al., 2017; Raj et al., 2019). Social norms frame sexual activity and schooling as incompatible for girls in many low‐ and middle‐income settings (Clark & Mathur, 2012; Eloundou‐Enyegue, 2004; Frye, 2017; Lloyd & Mensch, 2008). Yet, as a recent report assessing the economic effects of child marriage indicates, few studies have adequately measured the impact of child marriage on education outcomes, in part, because decisions about marriage timing and schooling are jointly determined (Wodon et al., 2017). Likewise, while adolescent childbearing clearly disrupts schooling, few studies in LMICs have determined whether the effect is causal. Child marriage and adolescent pregnancy are selective. The social and economic factors that predispose girls to marry early and bear children are also critical factors in premature school leaving including poverty, gender norms, perceived value of schooling and academic performance (Bajracharya et al., 2019; Lloyd & Mensch, 2008; Psaki, 2014). Studies have shown that girls who are behind in school may be more likely to engage in sexual activity, become pregnant and search for a marital partner (Clark & Mathur, 2012; Grant & Hallman, 2008). While observational data provide useful insights, randomized controlled trials (RCTs) that successfully reduce child marriage and adolescent pregnancy and also measure education outcomes may help identify their unique effects on school participation and learning among girls.

#### 2.1.3 Lack of information on returns to education/alternative roles for women (Barrier 3)

One reason given for families' reluctance to invest in girls' schooling relative to boys' is that less economic benefit is expected from educating daughters. This appears to be the case particularly in settings where adolescent marriage is the norm, where opportunities for paid employment among women are lower than for men and where earnings for women even for the same jobs are lower (Colclough et al., 2000). Research has shown that perceived returns to education affect the demand for schooling; furthermore, where perceived returns are underestimated, interventions that provide accurate information may raise educational attainment (Jensen, 2010). In addition, expanding economic opportunities for women and providing new information on alternative roles for women may increase girls' educational aspirations and human capital investment. However, a recent systematic review identified only four studies meeting their inclusion criteria that provided information to families on the returns to education and because the four studies assessed different outcomes, the benefits of providing that information for school participation and learning were not clear (Snilstveit et al., 2015).

#### 2.1.4 School‐related gender‐based violence (SRGBV) (Barrier 4)

Gender‐based violence in and around schools is believed to have consequences for school attendance, learning and attainment for all children, but particularly for girls (Global Education Monitoring Report Team and UNGEI, 2015). There is an extensive literature documenting SRGBV, perpetrated by both students and teachers, including sexual, physical and psychological abuse of students at, and in transit to, school (DevTech, 2004; Global Education Monitoring Report Team, 2015; Global Education Monitoring Report Team and UNGEI, 2015; Leach et al., 2003, 2014). While considerable attention has focused on sexual violence by boys or male teachers against girls, SRGBV takes many forms (UNGEI & UNESCO, 2013). SRGBV is said to reflect local attitudes and practices regarding the acceptability of corporal punishment and other forms of violence (Barasa et al., 2013; Crooks et al., 2007; Leach & Humphreys, 2007) as well as underlying social conditions and unequal gender relations (Parkes et al., 2013). A review for the 2003/4 UNESCO EFA Global Monitoring Report categorized gender‐based violence into behaviors and acts considered “explicit,” such as sexual harassment, rape and intimidation, and those considered “implicit,” including corporal punishment, bullying, verbal and psychological abuse, and teachers' use of “free labour.” The report asserted that because educational authorities had not taken sufficient action to combat SRGBV in many settings, especially sub‐Saharan Africa, it had flourished “unchecked” and become “institutionalised” (Dunne et al., 2003). SRGBV is thought to be most widespread in settings where other forms of inequality or disability are common (Parkes et al., 2016). However, despite assertions about the detrimental effects of SRGBV on education outcomes for girls in these settings, a 2013 report from UNGEI and UNESCO concludes that “we know little about how this violence impacts retention and achievement. The link is still tenuous…” (UNGEI & UNESCO, 2013).

#### 2.1.5 Gender insensitive school environment (Barrier 5)

While there are a number of studies documenting girls' treatment in the classroom relative to boys in high‐income countries (e.g., Sadker & Sadker, 1994) systematic assessment of classroom dynamics in LMICs is less common. To the extent that classroom observations have been conducted, studies indicate that teachers interact more often with boys, which is said to lead to “passivity” among girls (Global Education Monitoring Report Team, 2015). In addition, it has been argued that girls may perform better with more collaborative learner‐centred pedagogies in contrast to traditional teacher‐dominated “chalk and talk” teaching practices that prevail in many low‐ and middle‐income settings (Lloyd & Young, 2009; Mensch & Lloyd, 1998; Murphy‐Graham, 2009).

In addition to pedagogical practices that may be detrimental to girls, if teachers have traditional views about gender roles, prefer teaching boys or have lower expectations of girls, particularly with regard to science, technology, engineering and mathematics (STEM) subjects, this may have an adverse effect on girls' education outcomes (Lloyd & Mensch, 1999). While it has been argued that gender sensitivity of teachers can help transform gender norms (Global Education Monitoring Report Team and UNGEI, 2015; Sperling & Winthrop, 2015), few studies have examined teacher attitudes and their effect on students (Lloyd et al., 2000; Mensch & Lloyd, 1998).

More so than other dimensions of the school and classroom environment, the role of female teachers in improving educational outcomes for girls has been examined. While female teachers are not necessarily more supportive of girls' education than their male counterparts (Mensch & Lloyd, 1998), it may be that parents feel more comfortable sending their daughters to schools with female teachers and/or that female teachers act as role models for their students (Global Education Monitoring Report Team and UNGEI, 2015). Rigorous studies have examined the effect of teacher gender on the gap in test scores between boys and girls. A prior review indicated that exposing girls in LMICs to female teachers may be beneficial for education outcomes (Lloyd & Young, 2009). On the other hand, an analysis of the gender gap in mathematics using administrative data from Chile, which included measures of the school and classroom environment, did not find the gender of the teacher explained the gap in performance in grades 4 and 8 which doubled during that period (Bharadwaj et al., 2012).

#### 2.1.6 Lack of safe spaces and social connections (Barrier 6)

One potential barrier to adolescent girls' academic success is a lack of safe spaces to spend time and connect with their peers and trusted adults. Although they take different forms in practice, the goal of safe space groups, often led by female mentors, is to address social isolation, and often—through the delivery of life skills education—to build critical thinking and negotiation skills, address harmful gender norms, reinforce girls' commitment to staying in school, and/or strengthen support networks (Austrian et al., 2016; Temin et al., 2018). Proponents of these programs argue that the delivery of gender transformative content within an after school safe space platform could improve education outcomes for girls, especially in settings where girls' academic performance is weaker than boys. A recent narrative review of 30 “community‐based girls' groups” another label for safe spaces, identified nine such programs in Africa and South Asia, all multicomponent, that investigated the effect on education outcomes. To be considered for the review, the program had to include adolescent girls, a trained female mentor, a community venue where regular meetings could take place, employ an experimental or quasi‐experimental study design and be published between 2000 and 2017. Of the nine programs, six found significant effects on school participation or learning, which included numeracy, literacy, school enrolment, school retention and grade attainment. The nine programs measured a total of 18 education outcomes, of which 11 were significant (Temin & Heck, 2020). However, an important remaining question is whether safe spaces programs, on their own, are essential in driving improvements in education, or whether the content or structure of those meetings (e.g., the female mentor, the curriculum) or other components (e.g., cash or in‐kind transfers) lead to improvements in education outcomes (Population Council, 2018).

#### 2.1.7 Lack of teaching materials and supplies (Barrier 7)

Theoretically, lack of access to textbooks and educational materials is an obstacle to learning and may discourage enrolment and increase absenteeism. Girls may have differential access to teaching materials and supplies—for example, boys are more likely to own any type of phone and 80% more likely to own a smartphone compared to girls (Girl Effect and Vodafone Foundation, 2018)—this affects remote school participation and learning. There is also a potential gender issue in the form of textbook content. A comprehensive analysis of gender bias in textbooks found that it is a “hidden obstacle” to achieving gender equity in education in the global south (Blumberg, 2007). A review of studies documenting gender bias in textbooks for the UNESCO 2008 “Education for All Global Monitoring Report” described the following findings as being “near‐universal” (Blumberg, 2007, 2008):

- Women were found to be underrepresented relative to men;
- To the extent that women were depicted as engaged in work, they were primarily shown in domestic roles whereas men were shown in professional positions or in traditional male occupations;
- Positive images were highly gendered and included few overlapping characteristics with males portrayed as brave, strong, adventurous, hardworking, etc. and women as beautiful, loving, motherly, compassionate and dependent.

A 2015 update noted minimal progress since the earlier period (Blumberg, 2015). While many education practitioners concerned with gender inequity assume bias in textbooks undermines education outcomes for girls—and research in high income countries suggests it may do so (e.g., Good et al., 2010)—initiatives to modify textbooks in low income countries are rare due to the time and expense of revision and replacement (Blumberg, 2007). Given this constraint, it has been suggested that teachers develop classroom exercises to engage students in identifying textbook bias (Blumberg, 2008). A qualitative analysis of an innovative curriculum in Honduras demonstrated that when gender is incorporated into the curriculum such that teachers and students are encouraged to challenge traditional views of gender roles and relations, more equitable constructions of gender can emerge (Murphy‐Graham, 2009).

#### 2.1.8 Insufficient academic support (Barrier 8)

Insufficient academic support for girls may be reflected both in difficulties with school performance (e.g., repetition, poor exam scores), as well as lack of confidence in one's ability to stay in school and navigate an academic environment. Some empirical evidence has shown that poor school performance predicts subsequent dropout for girls and boys, which may reflect failure to meet academic standards (Hartley & Swanson, 1986; Soler‐Hampejsek et al., 2018), as well as girls becoming less invested in school and more likely to get married or become pregnant (Grant & Hallman, 2008; Marteleto et al., 2008). In settings where parents and community members already question the value of girls' education, poor performance may have more serious consequences for girls than boys. Previous reviews have examined the effectiveness of interventions designed to provide academic support to students but have largely lacked a gender lens. As part of a comprehensive review of the effect of school and teacher characteristics on education outcomes in developing countries, the effects of tutoring on education outcomes were positive in all four studies and significant in two (Glewwe et al., 2011). After school remedial education in core skills that target disadvantaged students and those lagging behind also appear promising, although results have not been systematically disaggregated by sex (Glewwe & Muralidharan, 2016; Snilstveit et al., 2015). To our knowledge, efforts to increase girls' academic self‐confidence have not been reviewed previously, and are often incorporated into other program components, such as safe spaces or life skills education.

#### 2.1.9 Inadequate sports programs for girls (Barrier 9)

Advocates of expanding girls' participation in sports in LMICs argue that sports challenge traditional gender scripts and transform the way girls view themselves and the ways their families and communities regard them. If true, supporters of these programs argue that expanding girls' access to sports facilities or equipment at school, as well as opportunities to practice leadership, engage in teamwork, develop physical skills, and/or increase girls' presence in outside areas where sports are played, may not only have positive effects on academic outcomes, but may also lead to increased empowerment (Brady, 2011, 2016; Sperling & Winthrop, 2015). Research on a natural experiment in the United States, taking advantage of a policy change (Title IX) that mandated equal opportunities for girls and boys to participate in sports, provides support for this relationship in high‐income countries. One study found a significant positive effect of high school sports participation on college attendance among young women as well as a significant effect on labor force participation and employment in male‐dominated occupations (Stevenson, 2010).

#### 2.1.10 Inadequate health and childcare services^1^ (Barrier 10)

While there is reason to believe that the provision of health and childcare services at school would improve educational outcomes, particularly absenteeism, a review of seven studies published between 1993 and 2003 that investigated the association between school‐based health clinics providing such services as vision and hearing screening, immunization, treatment for chronic and acute illnesses and mental health counseling and a variety of education outcomes including attendance, promotion, suspension, test scores and grade point average in the US concluded that there is “insufficient evidence to demonstrate a link” (Geierstanger et al., 2004). In addition to documenting considerable variability in findings across these studies, the authors noted numerous methodological limitations including the absence of study designs that control for confounding and selectivity. However, two more recent studies in the United States that used propensity score analysis to control for selection bias found significant effects of school‐based health center use on attendance, school dropout and grade point average (Kerns et al., 2011; Walker et al., 2010). As far as the provision of childcare services is concerned, even in countries that have liberalized policies, returning to school as a mother is rare, at least in sub‐Saharan Africa (Lloyd & Mensch, 2008). Thus, even if the provision of childcare services has an effect on education outcomes, it may not be large unless other systems are put in place to support adolescent mothers.

#### 2.1.11 Inadequate life skills (Barrier 11)

Life skills education aims to foster social and emotional skills such as communication, empathy, resilience, perseverance, agency, and critical thinking, build knowledge about sexual and reproductive health and one's body, and/or foster more equitable gender attitudes, respect for all people's rights, and equal power in relationships. These skills and mindsets are believed to contribute to better schooling outcomes, success in the work world, and better health and wellbeing. Indeed, economists and psychologists have shown that the predictive power of traits such as self‐control and perseverance may be as or more important for schooling, wages, and other socioeconomic outcomes than cognitive indicators such as IQ (Borghans et al., 2008; Heckman et al., 2013). Multiple reviews, primarily of studies from high income countries, have found significant associations between social and emotional skills and education outcomes, including meta‐analyses of school‐based interventions aiming to enhance students' social and emotional learning (Corcoran et al., 2018; Durlak et al., 2011; Taylor et al., 2017), as well as of after school programs (Durlak et al., 2010) and early childhood education (Blewitt et al., 2018). Sexuality and human immunodeficiency virus (HIV) education may contribute to improved schooling outcomes through multiple pathways. To the extent that sexuality and HIV education decreases pregnancy, teaches important social and emotional skills such as assertive communication and critical thinking, or fosters more equitable views on gender norms, it may contribute to improved education outcomes. An evaluation of the Teen Outreach Program in the United States, a curriculum‐based program that addressed many of these issues, found reduced adolescent pregnancy and reduced school failure (Allen & Philliber, 2001).

#### 2.1.12 Inadequate menstrual hygiene management (MHM) (Barrier 12)

Over the past 15 years, increasing attention has been directed towards inadequate MHM as a possible barrier to girls' education (Phillips‐Howard et al., 2016; Sommer et al., 2016; Sommer, Hirsch, et al., 2015; Sommer, Sutherland, et al., 2015). Much of this attention has been driven by qualitative research in LMICs. In a systematic review and meta‐synthesis of the qualitative literature on women and girls' menstrual experiences, Hennegan et al. (2019) produced an integrated model of menstrual experience. The model posits that socio‐cultural factors—including social support, behavioral expectations and knowledge—and resource limitations that dictate girls' physical and economic environments, influence girls' social participation, education, psychological and physical health through six dimensions of menstrual experience: shame and distress; confidence; menstrual practices; perceptions of environment and menstrual practices; containment of blood, odours, and materials; and individual menstrual factors, including experiences of symptoms related to menstruation. Interventions and policies focused on enhancing MHM have largely focused on the provision of menstrual management materials, improvement in school water, sanitation and hygiene infrastructure, and delivery of puberty education (Bobel, 2019; House et al., 2012; Sommer, Sutherland, et al., 2015; UNICEF, 2019). An increasing number of observational studies, many using a cross‐sectional design, have attempted to quantify the relationship between girls' menstrual experiences and their education outcomes. While some studies find that girls report that menstruation is negatively associated with school attendance or girls' ability to participate in school activities (Esen et al., 2016; Sivakami et al., 2019; Vashisht et al., 2018), others find little evidence for these relationships (Grant et al., 2013). Weak study designs, and varying study populations and challenges in accurately measuring absenteeism make drawing inferences about the level of absenteeism attributable to menstruation difficult (Grant et al., 2013).

#### 2.1.13 Lack of water and sanitation (Barrier 13)

School‐level factors dealing with water, sanitation, and hygiene (WASH) infrastructure have been cited as potential barriers to schooling for boys and girls given the importance of proper sanitation to avoid illnesses that cause absenteeism, to ensure children are properly hydrated during the school day, as well as potentially for gender‐related reasons including safety and the need for privacy and running water during menses (Alam et al., 2017; Davis et al., 2018; Miiro et al., 2018; Sperling & Winthrop, 2015; Vashisht et al., 2018). Sustainable Development Goal 6 focuses on clean water and sanitation, with a target of achieving access to adequate and equitable sanitation and hygiene for all by 2030 (UN General Assembly, 2015). UNICEF recommendations for menstrual health and hygiene‐responsive toilets in LMICs include: having sex‐separate toilet facilities, water access at a facility, presence of a door, having locks on doors, and including a pit or bin for disposal within a facility (Morgan et al., 2017; UNICEF, 2019). In a survey of 227 schools across six countries—Ethiopia, Kenya, Mozambique, Rwanda, Uganda, and Zambia—Morgan et al. (2017) found that less than 20% of schools in each country had at least four of the recommended services. While separate‐sex toilet facilities were most commonly reported, ranging from 44% in Ethiopia to 98% in Uganda, a median of 13% of schools had locks for latrine doors and a median of 9% reported having a water supply at the latrine facility (Morgan et al., 2017). Previous reviews focused on the effects of WASH interventions on education outcomes were unable to identify sufficient evidence of this relationship (Birdthistle et al., 2011; Jasper et al., 2012).

#### 2.1.14 Inadequate school access (Barrier 14)

Children's access to school is hampered by lack of schools or schools that are too far away, overcrowded and poor quality, and schools that lack flexible hours. School access is thought to particularly impact girls due to intersections with gender biases such as parental concerns about girls' safety, the disproportionate burden girls carry in terms of caring for younger siblings and household chores, and beliefs that educating girls is not worthwhile. Interventions or policies designed to improve school access may aim to make it easier for students to get to school, or to increase the hours they spend in school (Glewwe & Muralidharan, 2016). Distance to school may act as a barrier to enrolment and attendance, especially for girls in settings where they are unable to walk or travel to school on their own. Even when schools exist in close proximity, there may not be enough space for all students who want to attend, and parents may not be inclined to send their children if school infrastructure is perceived to be low quality (Li & Liu, 2014; Lloyd et al., 2005; Sharma & Levinson, 2019). Further, the timing of classes may not work with students' schedules—for example, if girls need to take care of siblings or their own children (Sperling & Winthrop, 2015). Previous reviews have found evidence that addressing access to school may increase enrolment and attainment, and possibly learning, for students overall (Glewwe & Muralidharan, 2016; Snilstveit et al., 2015), but have focused less attention on the question of gender differences in the effects of these interventions, unless they specifically targeted girls.

#### 2.1.15 Poor policy/legal environment (Barrier 15)

Policy and legal environments have the potential to address gender‐related barriers to girls' schooling in two main ways: by guarding against the negative effects of gender norms and practices, such as forcing pregnant girls to leave school; and by directly removing barriers, such as school fees. In practice, such policies often take one of three forms: enacting free and/or compulsory schooling, allowing for automatic promotion (AP) between primary and secondary schooling, or allowing re‐entry of pregnant girls and mothers into formal education. In areas with higher levels of poverty, without compulsory schooling policies in place, families may opt not to send girls to school and instead have them work for wages or in the home (Herz et al., 1991). A lack of AP policies may lead to high dropout between primary and secondary schools, especially among the most vulnerable (Ahmed et al., 2019). Without policies to formally allow for school re‐entry, girls who experience early pregnancy may be shut out of the education system no matter their past school performance. Supplemental programs that provide information to policymakers and local stakeholders to improve attitudes and information about the school re‐entry policy may help reduce remaining barriers to girls' re‐entry into school (Sperling & Winthrop, 2015; Walgwe et al., 2016).^2^ Finally, many policies exist, such as increased national oversight of teacher attendance or community‐based monitoring, that have been reviewed previously, but are not clearly designed to address gender‐related barriers (Glewwe & Muralidharan, 2016).

#### 2.1.16 Inability to afford tuition and fees (Barrier 16)

Families that have limited financial resources must choose how they allocate those resources, and for many the immediate benefits of girls' education may not outweigh the costs of schooling (Herz et al., 1991; Sperling & Winthrop, 2015). Tuition and school fees may be prohibitively high for poor families, and as mentioned above, parents may opt to keep boys in school rather than girls when there are greater financial constraints (Lloyd & Young, 2009). This is particularly the case during income shocks, which can exacerbate already unequal access to schooling between boys and girls (Björkman‐Nyqvist, 2013). Keeping girls out of school for these reasons can feed into a cycle of lower expected earnings for girls, which puts the next generation in a further disadvantaged position schooling‐ and earnings‐wise (Moore, 2005). Many countries adopted free primary education policies as a strategy to address this continuing issue and as part of their Millennium Development Goals commitments (Inoue & Oketch, 2008; Oketch & Rolleston, 2007), while NGOs and international institutions such as the World Bank have implemented or funded projects to increase school participation among girls by providing tuition and fee waivers (Rose, 2009; World Bank, 2016).^3^

#### 2.1.17 Inability to afford school materials (Barrier 17)

In a similar vein as tuition and fees, girls in the poorest households may not be able to attend school due to the costs of buying exercise books, clothes for school, and writing implements (Kadzamira & Rose, 2003). The cumulative cost of school materials can reinforce gender inequalities in school attendance and completion (Chapman & Mushlin, 2008). This may be an issue even when tuition and fees are waived, as households are typically expected to bear the entire cost of purchasing these supplementary materials for school, and choice overload may disincentivize adults from purchasing each additional tool for their children (Blocker et al., 2013; Jackson, 2011; Williams et al., 2015). Parents and guardians may allocate more school materials such as books to boys than to girls, which reflects and reinforces perceptions of lower importance in education for girls (Chimombo et al., 2000). In addition, the prices for uniforms for girls may be higher in communities where cultural concerns about safety and privacy translate into particular styles of dress (Herz et al., 1991). Indeed, the cost of school materials is recognized as a barrier to schooling by governments such as India and Zambia, which have implemented policies and programs that make uniforms noncompulsory, or distribute materials such as book bags and uniforms for free to individuals and households (Department of Public Instruction Bengaluru, 2020; International Monetary Fund, 2005). Indeed, interventions that provide material incentives for schooling are among those that have been noted in other reviews to have the potential to improve education outcomes for children (Snilstveit et al., 2015).

#### 2.1.18 Lack of adequate food (Barrier 18)

School feeding programs in the form of in‐school lunches and take‐home rations can be considered yet another way to reduce the cost of education and, at the same time, incentivize parents to send girls to school. These types of interventions also have the added potential benefit of reducing malnutrition among school‐aged children, which is commonplace in low‐ and middle‐income settings—30.3% of children in Africa and 23.2% of children in Asia are stunted (Global Nutrition Report, 2018). Although some studies disaggregate findings by gender, few have assessed the causal relationship between malnutrition and schooling outcomes such as academic performance focusing specifically on girls, despite the fact that girls in resource‐constrained settings are less likely to get access to food and thus might benefit disproportionately from school feeding programs (World Food Programme, 2020). However, we might infer how malnutrition may affect girls through observational studies on anaemia and iron deficiency, which are known to be detrimental to cognitive health and are a common occurrences among girls in LMICs (Bahrami et al., 2019; Balarajan et al., 2011). The immediate reductions in school performance due to reduced cognitive capacity may have negative downstream effects in terms of educational and labour market outcomes, putting adolescent girls at a further disadvantage in the long run (Akramipour et al., 2008; Halterman et al., 2001; More et al., 2013). This may in turn have detrimental intergenerational impacts as well—experiences of childhood anaemia by parents has been associated with increased odds of anaemia in their children, which may further exacerbate already existing gaps in education (Onyeneho et al., 2018).

### 2.2 The interventions

**Figure 1:**
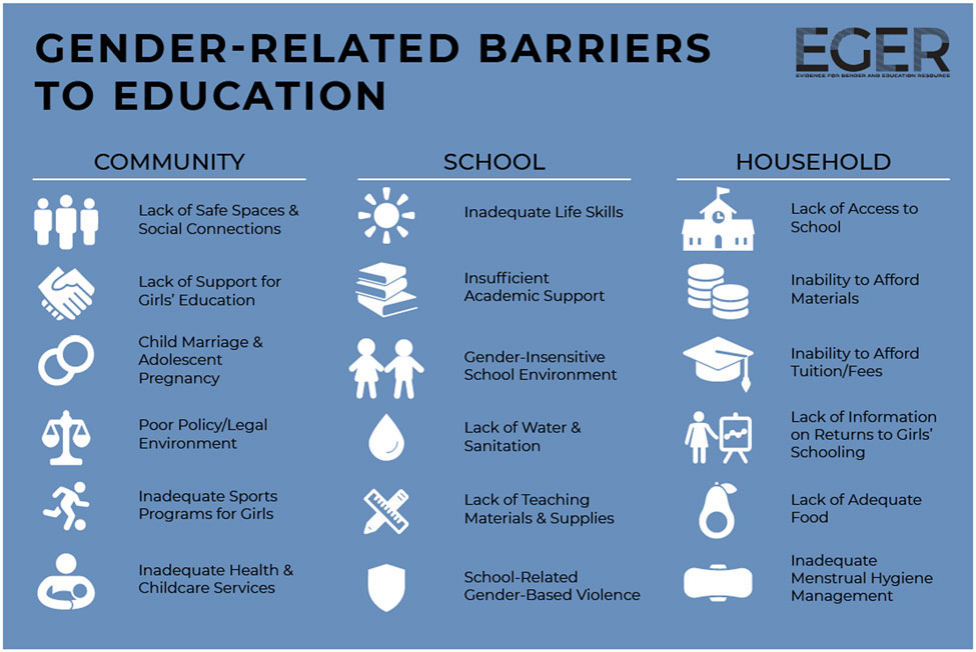
Hypothesized gender‐related barriers to education for girls

One of the goals of this review is to identify the variety of interventions aiming to address gender‐related barriers to girls' schooling that have been evaluated. We described above commonly perceived barriers and the ways those barriers may potentially affect girls' schooling. The extent to which these barriers exist varies between settings—both between and within countries—and to our knowledge has not been studied comprehensively. While a comprehensive list of possible interventions also does not exist, we provide examples of interventions that potentially address each perceived gender‐related barrier to education (see Figure 1).^4^

For this paper we divided gender‐related aspects of the community and school‐environments into the 18 barriers listed both in the background section and below. Our descriptions of the gender‐related barriers are rather expansive both because the barriers have not been precisely defined in the literature and because we wanted to be as inclusive as possible in assessing interventions that address potential obstacles to girls' schooling. Possible interventions targeting each of the barriers are provided below. These lists provide examples and are by no means exhaustive of possible approaches, especially given diversity of contexts and as the field continues to evolve.

1. Interventions that challenge the *lack of support for girls' education* may:
  1. Change community knowledge and norms about the value of girls' education, for example through community‐wide information campaigns on the benefits of girls' schooling;
  2. Change community and parents' attitudes about domestic responsibilities for girls that affect school participation;
  3. Change teachers' and school administrators' attitudes related to girls' education through information and training programs.
2. Interventions that address *child marriage and adolescent pregnancy* may:
  1. Change community norms, and parental and girls' attitudes and behaviours to reduce child marriage;
  2. Provide information about the legal age at marriage;
  3. Provide information about employment opportunities as an alternative to early marriage and childbearing;
  4. Provide a financial incentive to delay marriage;
  5. Provide information about family planning.
3. Interventions that target the *lack of information on returns to education/alternative roles for women* may:
  1. Provide information on paid employment for educated girls;
  2. Assist educated girls in obtaining paid employment;
  3. Challenge traditional gender role norms through examples of women's professional, scientific, leadership, etc., success and achievements.
4. Interventions that have a goal to reduce SRGBV may:
  1. Modify school policies and practices to create a safer environment, for example through the development and implementation of codes of conduct and safety policies;
  2. Change students' knowledge and attitudes about violence, for example by developing and implementing antiviolence curricula/activities for students;
  3. Change teacher and school administrator behaviour, for example by training school personnel on prevention and reporting of violence.
5. Interventions that address a *gender insensitive school environment* may:
  1. Train teachers in gender responsive pedagogy;
  2. Recruit, train and retain female teachers;
  3. Create book, math and science clubs for girls;
  4. Establish clinics or tutoring sessions for girls, for example in STEM subjects;
  5. Change teachers' and school administrators' attitudes related to the importance of girls' schooling, for example through information and training programs.
6. Interventions that address the *lack of safe spaces and social connections* may;
  1. Create female mentored girls' groups after school;
  2. Implement social asset building programs for girls.
7. Interventions that address the *lack of teaching materials and supplies at school* may:
  1. Ensure girls have access to classroom‐specific materials such as textbooks;
  2. Modify textbooks to ensure that gender stereotyping is eliminated.
8. Interventions that address *insufficient academic support* (in settings where girls are falling behind academically) may:
  1. Provide after school group remedial education in core skills;
  2. Provide individual tutoring;
  3. Assist in addressing problems related to school attendance.
9. Interventions that deal with *inadequate sports programs for girls* may:
  1. Institute school policies to ensure that girls get equal access to sports facilities;
  2. Provide sports equipment for girls at school;
  3. Offer new sports programs for girls, or extend existing programs to include girls, at school.
10. Interventions that address *inadequate health and childcare services* at school may:
  1. Provide onsite preventative and therapeutic health care services;
  2. Provide onsite childcare to girl students who are mothers or who are responsible for younger siblings.
11. Interventions that address *inadequate life skills* at school may:
  1. Improve girls' sexual and reproductive health knowledge, including knowledge about prevention and treatment of HIV/AIDS;
  2. Build empowerment and psycho‐social skills (social and emotional skills such as resilience, or communication).
12. Interventions that address *inadequate MHM* may:
  1. Provide free or subsidized sanitary products (sanitary pads and/or underwear);
  2. Provide free or subsidized analgesics for physical discomforts (cramps and headaches);
  3. Educate girls and others about MHM.
13. Interventions that address the *lack of water and sanitation* may:
  1. Provide new sources of water at schools by, for example, drilling more boreholes;
  2. Construct hand‐washing stations at schools;
  3. Construct/improve school toilets;
  4. Provide single sex toilets.
14. Interventions that address *inadequate school access* may:
  1. Increase the number of schools available to girls, for example, through building of community schools;
  2. Increase the availability of school transport for girls, for example, through provision of bicycles or school buses or “walking bus” programs (where school children are chaperoned by parents and/or community volunteers with the adults acting as a “driver” and “conductor” along a set route);
  3. Increase boarding opportunities for girls at school;
  4. Provide flexible school schedules.
15. Interventions that address a *poor policy/legal environment* may:
  1. Raise awareness about existing laws/policies among students, teachers and parents, for example those allowing pregnant girls to remain in school and return to school after childbirth;
  2. Develop or promote new laws/policies, such as increasing the number of compulsory years of schooling.
16. Interventions that address an *inability to afford tuition and fees* may:
  1. Provide stipends directly to the school to reduce or eliminate tuition and/or other school fees.
17. Interventions that address an *inability to afford school materials* may:
  1. Provide school materials such as textbooks and uniforms to the household or to the school, which are then distributed to households or students.
18. Interventions that address the *lack of adequate food* may:
  1. Provide school lunches, take‐home rations or other food within schools to students;
  2. Provide food to students or households on the condition that students attend school or achieve some minimum school‐related performance goal.

We initially provided an “other” category to include papers analysing interventions that address a gender‐related barrier not listed in our protocol. Such interventions would address situations in which the authors assert that there are assets, activities or facilities for which a gender difference in access exists. Based on our search, we either identified barriers that were not covered in our initial list or separated out barriers that were initially grouped with other barriers based on the prevalence of such studies among those identified for inclusion. These added barriers are now included as separate barriers, and have been listed above as well:

- Lack of teaching materials and supplies
- Lack of safe spaces and social connections
- Lack of information on returns to education/alternative roles for women
- Child marriage and adolescent pregnancy
- Inability to afford tuition and fees
- Inability to afford school materials
- Lack of adequate food.

### 2.3 How the intervention might work

**Figure 2:**
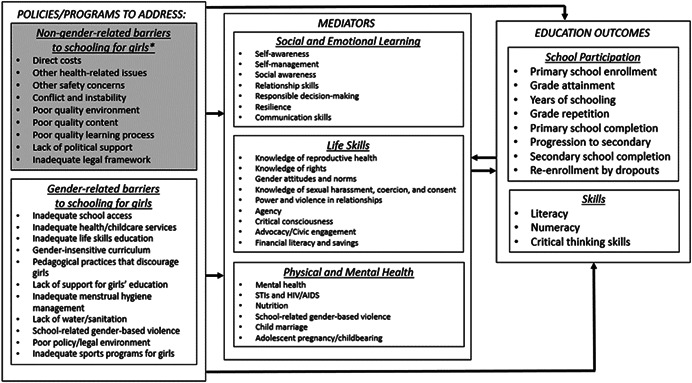
Conceptual framework linking barriers to education for girls to outcomes

The conceptual model (Figure 2) maps our hypothesized gender and non‐gender‐related barriers to schooling for girls that we expected included interventions to address, the antecedents that underlie these barriers, as well as the mediators and education outcomes that are potentially affected by those barriers. We note that whether and how an intervention may work to eliminate a barrier is context dependent, but given the wide range of barriers and interventions examined, it is beyond the scope of this review to give full consideration to all these possible theories of change. Note that we added additional gender‐related barriers to education based on the findings of the review, and the full list is included in Figure 1.

While the gender‐related barriers to schooling vary between settings, as do the appropriate interventions to address those barriers, many of those interventions share a similar underlying logic. For example, interventions may target parents' attitudes about the value of girls' education using different approaches, including sharing information about employment opportunities for women, or shifting norms by making a rights‐based argument through community meetings. Given parents' roles in deciding whether their children will attend school, and providing financial resources to support schooling, such interventions might have a direct effect on school enrolment and retention. More indirectly, teacher training programs that equip teachers with the skills to understand and address the learning needs of both girls and boys may lead to more active participation of girls in the classroom, leading to stronger communication skills and more ambitious educational aspirations for girls, and subsequently leading to improved grade attainment and literacy for girls.

The primary outcomes of interest in this review are focused on educational attainment and skills. Although listed as mediators, understanding the effects of policies and interventions on child marriage and adolescent pregnancy, social and emotional learning, life skills, employment, and physical and mental health are important questions in their own right. To maintain a reasonable scope for this review, however, our inclusion criteria were focused on studies measuring school enrolment, educational attainment, and academic skills outcomes. Therefore, our results provide only a partial picture of the evidence on the effects of policies and interventions on child marriage and adolescent pregnancy, social and emotional learning, life skills, employment, and physical and mental health.

### 2.4 Why it is important to do the review

A number of literature reviews, some systematic, have assessed the evidence regarding the effectiveness of interventions to improve education outcomes in LMICs. The most comprehensive review to date, conducted by the International Initiative for Impact Evaluation (3ie) did not explicitly focus on gender‐related barriers except insofar as some of the interventions targeted girls, for example, conditional cash transfers and sex segregated toilets, in part reflecting the fact that many education evaluations do not examine heterogeneity in effects by sex (Baird et al., 2013; Snilstveit et al., 2015). However, the findings from the 3ie review are worth noting; programs were found to either improve school participation or learning, but rarely affected both. The strongest and most consistent evidence was observed for cash transfer programs in affecting participation, and structured pedagogy programs in affecting learning.

Several reviews have focused explicitly on girls' education, most notably those by Unterhalter et al. (2014) and Sperling and Winthrop (2015). But these reviews did not use rigorous and/or transparent criteria for inclusion or rating study quality, attributes required of systematic reviews, and did not provide quantitative syntheses. To the extent that systematic reviews have examined gender‐related barriers to girls' schooling, they have focused on specific barriers such as water and sanitation or MHM for example (Birdthistle et al., 2011; Hennegan & Montgomery, 2016; Jasper et al., 2012). Another study sought to identify the programs most effective for girls' access and learning, comparing interventions aimed at girls with general interventions. It concluded that interventions that included boys as well as girls were as effective in improving access and learning as girl‐targeted interventions (Evans & Yuan, 2021). The authors note that eliminating gender‐related barriers may improve girls' schooling experiences and may have additional long‐term benefits, including benefits for boys.

This systematic review departs from prior reviews in that we examine a broad set of barriers to assess which policies and interventions that include elements directly addressing gender‐related barriers to girls' schooling are effective in improving school participation and learning. That is, rather than focusing on all possible interventions that might improve outcomes for girls, we examine interventions designed—either explicitly or implicitly—to address gender‐related barriers to education for girls.

The protocol for this systematic review was peer reviewed and registered with the Campbell Collaboration and can be accessed here: https://onlinelibrary.wiley.com/doi/abs/10.1002/cl2.1003.

## 3 OBJECTIVES

### 3.1 What are the effects of interventions that address gender‐related barriers to schooling on girls' education?

The objective of this systematic review is to summarize and assess evaluations of policies and interventions designed to address gender‐related barriers that are believed to undermine girls' school participation and learning. That is, rather than a review defined by the outcomes that interventions try to achieve, or the specific intervention approaches, we define our inclusion criteria by the barriers interventions are designed (implicitly or explicitly) to address. We also limit our review to gender‐related barriers that are perceived to disadvantage girls, although we acknowledge that barriers also exist that may disadvantage boys disproportionately. Therefore, we define gender‐related barriers as factors that prevent girls from enrolling, attending, fully participating and/or learning in school. Barriers may exist at the individual, household, community, school or policy levels. We build on the work of UNICEF, UNESCO, the Global Partnership for Education and the United Nations Girls' Education Initiative on barriers to girls' education to collate the list of perceived gender‐related barriers (Albright, 2016; Antoninis et al., 2018; GPE Secretariat, 2016; UNICEF, 2002).

The primary research question we address is: what is the effect of interventions to eliminate gender‐related barriers to girls' education on girls' primary and secondary school participation and learning outcomes in LMICs? Specifically, what is the effect of these interventions on:

- Enrolment and grade attainment for girls? (Grade attainment includes years of schooling, highest grade completed, completion of primary school, transitioning from primary to secondary school, completion of secondary school and re‐entry for girls who have dropped out).
- School attendance for girls?
- Grade repetition for girls?
- Learning for girls? Including academic skills, particularly literacy and numeracy, as well as non‐verbal reasoning.

Given the lack of a shared framework defining gender‐related barriers to education, and associated interventions, part of the work of this review is also to describe the types of interventions that may address these barriers.

While we identified a series of secondary outcomes in our review protocol, including attitudes/abilities, knowledge, physical and mental health, and resource access, we found that these were not included in identified papers frequently or consistently enough to offer any insights on mechanisms linking interventions to schooling outcomes. Conversely, as only studies that assessed education outcomes were included in our review, the papers in this review that also report secondary outcomes provide only a narrow slice of the literature on these outcomes. We therefore do not include secondary outcomes in this report, however the extracted data are available in Table S1.

## 4 METHODS

The following section outlines our methodological approach to the review. These methods are largely based on the Cochrane Handbook, *Practical Meta‐Analysis* by Mark Lipsey and David B. Wilson, *Research Synthesis and Meta‐Analysis: A Step‐by‐Step Approach* by Harris Cooper (Cooper, 2015; Higgins & Green, 2011; Lipsey & Wilson, 2001), and “Rating the certainty in evidence in the absence of a single estimate of effect” (Murad et al., 2017).

### 4.1 Criteria for considering studies for this review

To assess the current evidence on interventions to address gender‐related barriers to schooling for girls, studies published before 2000 were excluded. We did not exclude studies based on language, nor did we exclude grey literature from our search.

#### 4.1.1 Types of studies

We limit our review to studies that use sufficiently rigorous methods to provide valid causal estimates of the effects of interventions on the outcomes of interest (i.e., that attempt to control for endogeneity^5^). Those methods included^6^:

- RCTs (longitudinal data, or post‐intervention data for studies with large sample sizes^7^)
- Regression discontinuity (longitudinal or cross‐sectional data)
- Instrumental variables analysis (longitudinal or cross‐sectional data)
- Difference‐in‐differences (longitudinal or cross‐sectional data)
- Other quasi‐experimental studies with either:
  - A matched comparison group where the matching procedure is described (e.g., nearest‐neighbour matching, propensity score matching) OR
  - A comparison group where quasi‐treatment and quasi‐control groups are either stratified or tested for balance, based on more than one sociodemographic characteristic justified by background literature
- Interrupted time series (longitudinal data)

Studies reporting on both RCTs and quasi‐experiments were eligible for inclusion. Articles that utilize study designs outside of those listed above were excluded, notably:

- RCTs that only report post‐intervention data from a small‐scale intervention
- Quasi‐experimental studies without a pre‐post or quasi‐control group comparison.
- For studies that employ matching, no formal matching method was stated.

#### 4.1.2 Types of participants

We included studies that report education outcomes for girls, and/or interact results by sex. Studies that only reported results for boys, or combined girls and boys without reporting an interaction by sex, were excluded.

#### 4.1.3 Types of interventions

Below is the list of gender‐related barriers identified from the literature, or subsequently added based on the findings in our review. Descriptions of the types of interventions that are hypothesized to address these barriers are listed in the background section. Studies flagged for inclusion in the search present the results of interventions/exposures that attempted to remove or minimize one or more of the following barriers—otherwise they were excluded:

1. Lack of support for girls' education
2. Child marriage and adolescent pregnancy
3. Lack of information about returns to education/alternative roles for women
4. School‐related gender‐based violence
5. Gender insensitive school environment
6. Lack of safe spaces and social connections
7. Lack of teaching materials and supplies
8. Insufficient academic support
9. Inadequate sports programs for girls
10. Inadequate health and childcare services
11. Inadequate life skills
12. Inadequate menstrual hygiene management
13. Lack of water and sanitation
14. Inadequate school access
15. Poor policy/legal environment
16. Inability to afford tuition and fees
17. Inability to afford school materials
18. Lack of adequate food.

Although a common intervention approach, cash transfer programs targeting individuals and households were not included in this review because there have been several recent systematic reviews that have analyzed these interventions (e.g., Baird et al., 2013; Snilstveit et al., 2015).

Multi‐component interventions in which one of the components is addressing gender‐related barriers were included. Interventions and policies may take place at the primary or secondary levels or may be nonformal for school‐aged young people. We identified a handful of multi‐component programs that fit into one or more of the above‐listed barriers, but also included a cash transfer component at the individual or household level. These studies have been flagged in Table 1 due to the inability to separate out the effects of the cash transfers from the effects of other components of the interventions.

**Table 1:** Study descriptions

Author (year)	Country	Study description
Aber et al. (2017)	Democratic Republic of the Congo	Provided teacher resource materials for a primary school reading curriculum with a social‐emotional learning focus; included collaborative school‐based teacher learning circles
Adelman et al. (2017)	Haiti	Annual per‐student payment directly to schools in exchange for not charging tuition to students
Adukia (2016)	India	National school latrine construction program, assessing the effects of unisex versus single sex latrines based on the age group of school‐aged girls
Agüero and Bharadwaj (2014)	Zimbabwe	1980 education policy reform (1) made primary education free and compulsory, (2) removed age restrictions for older children to enter school, (3) community support for education, and (4) automatic grade progression.
Akresh et al. (2018)	Indonesia	National school construction program
Andalon et al. (2014)	Mexico	The Mexico's National Agreement for the Modernization of Basic Education in 1992 had two main elements: (1) compulsory education was extended from 6th grade to 9th grade, (2) 6188 public lower secondary schools were built between 1993‐1998.
Argaw (2013)	Ethiopia	There were two major parts of the 1994 education reform: (1) introduction of mother tongue instruction in primary education; and (2) abolition of the central primary school exit exam. The latter was applied in all regions, while the former varied across regions.
Asadullah and Chaudhury (2013)	Bangladesh	Established NGO (Bangladesh Rural Advancement Committee—BRAC) primary schools which target out of school children from poor families particularly girls; 97% of teachers reported to be female
Ashraf et al. (2018)	Zambia	Trained 8th grade girls in negotiation skills; provided safe space with female mentors; offered girls information about returns to education; multiarm
Aurino et al. (2018)	Ghana	Per‐student payments to local caterers to supply school meals to students
Austrian et al. (2020)	Zambia	Provided safe space to unmarried girls with female mentors leading sessions on life skills, sexual and reproductive health, HIV and financial literacy; other arms included these components as well as health vouchers and savings accounts; multiarm
Avitabile and de Hoyos (2018)	Mexico	Provided 10th grade students with information about earnings associated with different levels of (a) education, and (b) life expectancy; also provided information about funding opportunities for higher education
Bagby et al. (2017)	Niger	Constructed girl‐friendly primary schools, promoted gender equitable classrooms, constructed and maintained boreholes, supported school management committees, developed a student mentoring program, tried to motivate parents to keep children in school, promoted a culture of reading by building community support for reading and establishing adult literacy, trained and supported teachers in early grade reading, provided reading materials in local languages; multiarm
Bandiera et al. (2014)	Uganda	Provided vocational training and life skills training to adolescent girls including information on STIs, family planning, negotiation, conflict resolution and leadership as well as legal knowledge of child marriage, brideprice and violence against women; activities conducted within a “protective” space with female mentors
Bandiera et al. (2019)	Sierra Leone	Provided girls aged 12–25 with a “protective” space within the context of the Ebola crisis; female mentors facilitated meetings where information and support was provided on health and reproductive issues; NGO (BRAC) professionals provided vocational training
Barrera‐Osorio et al. (2017)	Pakistan	Schools receive either a “gender‐uniform” subsidy (350 rupees per student) or a “gender‐differentiated” subsidy (350 rupees for each male student, 450 rupees for each female student); multiarm
Beg et al. (2018)	Pakistan	Delivered math and science content to grade 8 students via short multimedia video presentations and trained teachers in pedagogical techniques (blending standard face‐to‐face teaching with new technology)
Benshaul‐Tolonen et al. (2019); Phillips‐Howard et al. (2016)	Kenya	Three‐arm pilot cluster RCT in 30 primary schools; arms included: (1) one insertable menstrual cup, (2) 16 sanitary pads monthly, and (3) control (usual practice); multiarm
Blimpo et al. (2016)	The Gambia	Per‐student payment directly to schools based on the number of girls enrolled in the school
Buchmann et al. (2016)	Bangladesh	Implemented a 6‐month empowerment program for adolescent girls that included education support and a social competency component; also provided a financial incentive to delay marriage; community mobilization was conducted before implementation to inform parents, teachers and community leaders about the program and its potential benefits and to gain assistance in identifying safe spaces; multi‐arm
Burde and Linden (2009)	Afghanistan	Constructed community‐based schools, provided teacher and community training, administrative support, and materials to both teachers and students
Buttenheim et al. (2011)	Lao PDR	Daily snack and take‐home rations
Carney et al. (2019)	Uganda	The Educate! Experience program teaches secondary school students soft skills (e.g., communication, critical thinking, grit) and hard skills (e.g., budgeting, savings, etc). It consists of three components: Social entrepreneurship and leadership course taught in school (socially responsible leadership skills, business/entrepreneurship skills, community engagement, ‘personal projects', and group mentorship), one‐on‐one and group mentoring sessions, and student business club (guided by mentor, students design, start, and manage a business)
Caruso et al. (2014)	Kenya	Three‐arm intervention including: handwashing alone, handwashing plus latrine cleaning, and a control group; multiarm
Chatterjee (2017)	India	School construction targeting communities with households where literacy levels were low
Chicoine (2016)	Ethiopia	Proclamation No. 41, passed in 1993, increased the number of primary and secondary schools to nine newly formed regional authorities and two independent administrations in the country's two largest cities. The Education and Training policy, passed in 1994, required public education to be free for grades 1–10
Chin (2005)	India	Provision of additional teachers to all one‐teacher schools
Cho et al. (2019)	Kenya	Provided Grades 7 and 8 orphans (both single and double) with school uniforms and payment of secondary school fees; research staff monitored study participants' school attendance and assisted them with resolving absenteeism problems
Chyi and Zhou (2010)	China	A policy that capped rural tuition (i.e., tuition control), a tuition waiver policy, as well as a combined tuition waiver, free textbooks, and living stipend policy; multiarm
Datta Gupta et al. (2018)	India	Improvements school infrastructure and construction, hiring new teachers, textbook development, provision of textbooks, teacher training, mid‐day school meals
De Neve and Subramanian (2017)	Zimbabwe	A 1980 policy reform reduced academic and structural restrictions limiting advancement toward secondary school. (e.g., automatic grade progression to secondary school, large secondary school construction focused on rural areas)
Delavallade et al. (2014)	India	Trained village volunteers committed to girls' education; targeted those never enrolled and dropouts; provided instruction in core subjects for girls and boys in primary school
Duflo et al. (2014)	Kenya	Two programs conducted stand‐alone and jointly: Education Subsidy program which subsidized the cost of education by providing 2 free school uniforms over last 3 years of primary school; HIV Education program which provided training to 3 teachers in each primary school to help them deliver the national HIV/AIDS curriculum which emphasizes abstinence until marriage as the way to prevent infection; multiarm
Duflo et al. (2019)	Ghana	Full tuition and fee waivers paid directly to the school in exchange for not charging eligible students
Eble and Hu (2019)	China	Exposed middle school students to female teachers
Edmonds et al. (2016)	India	Provided life skills curriculum to girls in grades 6 and 7 via young women from area trained as “social mobilizers” (both mentor and act as role models); curriculum included problem solving and critical thinking, as well as such social and emotional competencies as relationship building and self‐control; mentors received training in providing girls with support services
Erten and Keskin (2018)	Turkey	Extended compulsory schooling to 8 years, school construction and improvement, large‐scale teacher hiring and training, distribution of computers to rural schools
Evans and Ngatia (2018)	Kenya	Uniforms provision
Freeman et al. (2012); Garn et al. (2013)	Kenya	Water treatment and hygiene promotion, additional sanitation improvement, or control group; multiarm
Giordono and Pugatch (2017)	The Gambia	Textbooks, notebooks, bed nets, uniforms, shoes and bags provision as well as mentoring
Grant (2015)	Malawi	In September 1994, the Government of Malawi eliminated all primary school fees across the country. This followed the slow elimination of school fees since 1991 in the form of fee waives for grades one and two in the first 2 years
Grépin and Bharadwaj (2015)	Zimbabwe	1980 policy reform that reduced academic and structural restrictions limiting advancement toward secondary school and included automatic grade progression from primary school to secondary school, and a large secondary school construction program, focused on rural areas
Grogan (2009)	Uganda	Uganda's universal primary education policy eliminated primary school fees beginning in 1997
Güneş (2016)	Turkey	The compulsory schooling law (CSL) increased the mandatory years of completed primary education from 5 to 8 years in 1997. They also created new schools, added new classes to existing schools, recruited new primary school teachers, provided transportation to children who lived far from schools, and provided free textbooks and uniforms to low‐income students
Hahn et al. (2016)	Bangladesh	Created study groups with friends or peers for primary students; multi‐arm
Hallfors et al. (2011); Iritani et al. (2016)	Zimbabwe	Provided the following school support: fees, exercise books, uniforms, and other school supplies, including sanitary napkins, underpants, pens, and soap. Female teachers were trained as helpers in each intervention school to monitor attendance and help reduce absenteeism
Heath and Mobarak (2014)	Bangladesh	Exposed girls to export oriented garment sector that provides employment opportunities to women in large scale
Hermida (2014)	Ecuador	Elimination of school enrolment fee
Hidalgo et al. (2010)	Ecuador	Free school uniforms
Hungi and Ngware (2017)	Kenya	Offered incentivized subsidy for girls to enroll in secondary school based on primary school leaving exam score; provided after‐school homework support in math, life skill mentoring and parental counselling to sensitize parents about the importance of girls' schooling; multiarm
Jacoby and Mansuri (2011)	Pakistan	Distance to nearby school
Jensen and Oster (2007)	India	Introduced cable television with modern lifestyle programming
Jensen (2012)	India	Provided recruiting services to help young women obtain jobs in the business process outsourcing industry
Johnston and Ksoll (2017)	Ghana	Remote learning via solar‐powered satellite and interactive software, lessons in English and math were interactive and delivered in real time. Classrooms also had in‐person facilitators to manage classrooms, etc. Also included daily satellite‐transmitted after‐school lessons for girls, some of whom had previously dropped out. The after‐school lessons focused on empowerment and health
Kaur (2017)	India	In‐school feeding
Kazianga et al. (2012); Kazianga et al., (2019)	Burkina Faso	Constructed girl‐friendly primary schools and provided additional amenities, including separate latrines for boys and girls, canteens, take‐home rations and textbooks, and “soft” components such as a mobilization campaign, literacy training, and capacity building among local partners
Kazianga et al. (2009)	Burkina Faso	In‐school feeding and take‐home rations; multiarm
Keats (2018)	Uganda	Uganda's universal primary education policy eliminated primary school fees beginning in 1997
Lakshminarayana et al. (2013)	India	Provided supplementary, remedial teaching and learning materials in classes two, three and four in public primary schools and additional materials for girls including uniforms, shoes, socks, undergarments and a school bag; also outreach programme to promote education; multiarm
Lehrer (2010)	Uganda	In‐school feeding and take‐home rations; multiarm
Lu and Anderson (2015)	China	Introduced seat assignment whereby girls sit near other girls in middle school
Lucas and Mbiti (2010)	Kenya	Free primary education
Makate (2016)	Uganda	Uganda's universal primary education policy eliminated primary school fees beginning in 1997
Mbiti et al. (2019)	Tanzania	Per‐student payments directly to the school, cash bonuses to teachers based on student performance; multiarm
McCadden (2015)	Zambia	School re‐entry program for pregnant girls to return to school after giving birth
Meller and Litschig (2015)	India	Two government schemes targeted to upper‐primary school‐age girls in rural and educationally “backward” areas: (a) funds for girl‐focused service (could be used for day care centers for younger siblings, flexible timing of classes, remedial classes, bridge courses for dropouts, vocational training) and (b) infrastructure projects including teaching and library materials and sports equipment; funding could be used for setting up separate classrooms for girls, installation of girls' toilets, and electrification; organized community workshops and requested parents to identify girls who had dropped out; also received funds to set up an additional girls' boarding school
Mensch et al. (2019)	Zambia	Provided an e‐reader literacy program for 7th grade girls after school embedded within a safe space empowerment program using female mentors; provided community engagement to support girls' schooling; multiarm
Morrell et al. (2014)	Malawi	Trained female teachers to run participatory, girl‐friendly, extracurricular activities focused on improving girls' self‐confidence, sexual and reproductive health and academic skills
Muralidharan and Prakash (2013)	India	Free bicycle provision to girls currently enrolled in secondary school (grade 9)
Muralidharan and Sheth (2013)	India	Exposed primary students to female teachers
Muralidharan et al. (2016)	India	Exposed middle school students to technology‐led after school instructional program customized to level and rate of progress of the individual student
Okurut (2015); Okurut (2018)	Uganda	Implemented automatic promotion from primary to secondary school
Osili and Long (2008)	Nigeria	The Universal Primary Education (UPE) program in Nigeria eliminated school fees for primary education, increased primary school construction and provided teacher training institutions across the country beginning in September 1976
Oster and Thornton (2009)	Nepal	Girls in the intervention group were given menstrual cups for use during their periods
Özler et al. (2020)	Liberia	Delivered a life skills curriculum to girls aged 13–14 weekly via a safe spaces platform with female mentors; provided a cash incentive payment to caregivers for girls' participation in the program; multiarm
Stark et al. (2018)	Ethiopia	Provided a weekly social empowerment program for adolescent refugee girls via a safe spaces platform with female mentors; provided discussion sessions for caregivers of adolescent girls
Sukontamarn (2005)	Bangladesh	Provision of NGO schools
Sukontamarn (2013)	Bangladesh	Free monthly food (grains) conditional on having at least one primary school‐age child attending school that month and being poor
Tequame and Tirivayi (2015)	Ethiopia	The 1994 Education and Training Policy (EETP) included two elements: (1) increasing the number of public higher education institutions, and (2) deregulation of private provision of higher education
Wilson et al. (2012)	Kenya	Girls in the intervention group were invited to join a training session on how to make reusable sanitary pads and provided with equipment to make three pads. They were given an instruction booklet to help them make the pads at home, as well as instructions on washing and drying the pads
Yamauchi and Liu (2011a, 2011b)	Philippines	School construction and improvements, textbooks provision, teacher training, school‐based management improvements, and other facility and equipment support
Yang et al. (2013)	China	Provided computer‐assisted learning program and laptop computers to primary school students

*Note*: A full description of study characteristics can be found online at https://dataverse.harvard.edu/dataverse/popcouncil

#### 4.1.4 Types of outcome measures

Only studies that measured the primary outcomes listed below were included. Outcomes of interest had to be measured consistently in all comparison groups in order for the study to be included.

##### 4.1.4.1 Primary outcomes

Below were the primary outcomes prespecified in the protocol.

1. Enrolment (limited to primary and secondary school)
  - Grade attainment
  - Years of schooling
  - Enrolment in primary school
  - Enrolment in secondary school
2. Retention
  - Grade repetition
  - Primary school completion
  - Progression to secondary school
  - Secondary school completion
  - Re‐enrolment in school among dropouts
  - Absenteeism
3. Learning and Cognitive Skills
  - Academic skills (literacy and numeracy) during and after leaving school
  - Critical thinking skills (e.g., Test of Science Critical Thinking (Mapeala & Siew, 2015))
  - Nonverbal reasoning (e.g., Raven's Progressive Matrix) (Raven, 2000).

##### 4.1.4.2 Secondary outcomes

Though we originally specified a list of secondary outcomes we were interested in, we did not identify enough effect sizes for each of our secondary outcomes of interest to be able to include the results in a meaningful way in our review. Further, since our search for secondary outcomes was not systematic, but rather opportunistic based on which studies met our inclusion criteria for primary outcomes, presenting those results may provide a biased perspective on the existing evidence, and conclusions, with regard to secondary outcomes. The original list of secondary outcomes can be found in the study protocol (Chuang et al., 2019) and we present the extracted data in Table S1. These areas would be better explored through a systematic review with a primary focus on these outcomes.

#### 4.1.5 Duration of follow‐up

We did not exclude studies based on duration of follow‐up but note duration in the data extraction table.

#### 4.1.6 Types of settings

Studies that reported on the primary outcomes listed above using data from LMICs at the time of the intervention/exposure, as defined by the World Bank (2018), were included. Studies that only reported on outcomes from high‐income countries, as defined by the World Bank, were excluded.

### 4.2 Search methods for identification of studies

#### 4.2.1 Electronic searches

The following databases were searched electronically:

**Table:** 

Database name	Platform	Web address
AEA RCT Registry	AEA	https://www.socialscienceregistry.org/trials/search?
Africa Bibliography	Cambridge Univ Press	https://africabibliography.cambridge.org/
African Education Research Database	REAL Center, ESSA	https://essa‐africa.org/node/501?action=searchadvanced
African Journals Online	AJOL	https://www.ajol.info/index.php/index/search
DEC USAID	USAID	https://dec.usaid.gov/dec/content/AdvancedSearch.aspx?
Dissertation Abstracts	ProQuest	https://search.proquest.com/…genre=dissertations+%26+thesesandsid=ProQ:ProQuest+Dissertations+%26+Theses+Global
EconLit	ProQuest	https://search.proquest.com/…genre=articleandsid=ProQ:ProQ%3Aeconlit
ELDIS	IDS	https://www.eldis.org/search
Epistemonikos	Epistemonikos	https://www.epistemonikos.org/en/advanced_search
ERIC	EBSCOhost	https://search.ebscohost.com/login.aspx?direct=trueanddb=eric
Evidence Hub	3ie	http://www.3ieimpact.org/evidence‐hub
Global Index Medicus	WHO Global Health Library	http://www.globalhealthlibrary.net
IDEAS‐Repec	IDEAS‐Repec	https://ideas.repec.org/search.html
Intl Clinical Trials Registry	WHO ICTRP	http://apps.who.int/trialsearch/
NBER	NBER	https://www.nber.org/papers.html
OpenGrey	INIST‐CNRS	http://www.opengrey.eu/search/
Open Knowledge Repository	WB	https://openknowledge.worldbank.org/
POPLINE	JHUCCP	https://www.popline.org/advancedsearch
PsychINFO	EBSCOhost	https://search.ebscohost.com/login.aspx?direct=trueanddb=psyh
PubMed	NLM. NIH	https://www.ncbi.nlm.nih.gov/pubmed/advanced
Research for Development Outputs	DFID R4D	https://www.gov.uk/dfid‐research‐outputs
ScienceDirect	Elsevier	https://www.sciencedirect.com/search?
Sociological Abstracts	ProQuest	https://search.proquest.com/…genre=articleandsid=ProQ:ProQ%3Asocabs
Web of Science	EBSCO/Reuters	https://search.ebscohost.com/login.aspx?direct=trueanddb=web

Grey literature was identified using the databases listed above from DEC, ELDIS, OpenGrey, and DFID R4D. Studies published in all languages were included. Additional unpublished/ongoing studies were identified through searches of websites of specific organizations identified in the search to be key resources. These organization websites include: the Center for Global Development, CARE, CEDPA, High‐Quality Technical Assistance for Results (HEART), International Center for Research on Women (ICRW), J‐PAL (Poverty Action Lab), the Population Council, UNESCO, UNGEI, and UNICEF. References from these websites were reported in a general category (World Wide Web, i.e., WWW). The search strategy documented in Supporting Information Appendix Section A was used to conduct searches through ERIC and was adapted to conform to the search functions of the other databases. Searches were conducted in March and April of 2019.

#### 4.2.2 Searching other resources

Reference lists and bibliographies in relevant review articles and reports of systematic reviews found in the search were also checked to identify additional articles eligible for inclusion. Reviewers contacted the authors of included studies as well as other relevant researchers and organizations to locate additional articles eligible for inclusion.

We concluded extra searches through databases, websites, reference lists, and so forth, in March of 2020.

### 4.3 Data collection and analysis

#### 4.3.1 Selection of studies

Articles were identified through searching the databases listed in the search strategy shown in Supporting Information Appendix Section A. Depending on the structure of each database, we used the inclusion criteria to further filter results when possible, excluding studies that were out of geographic scope, topical scope (e.g., included only animals), or describing girls' education without examining an intervention, as well as removing duplicates. Each title and abstract was screened by a set of two reviewers based on the inclusion criteria documented above through Covidence. Due to the large number of articles identified in the search, we employed a team of Masters and PhD‐level reviewers in related disciplines to assist with selection of studies (Fiona Gambanga, Nura Anwar, Rachel Passmore, Lili Warren, Isabel Odean, Anne Smith, Grace Sheehy, Aditi Patrikar, Jeanette Shekelle). Before any review activities, all reviewers were trained on a random set of 10 articles to gain familiarity with voting procedures as well as address any gaps in understanding about the inclusion criteria. Randomized selection of articles was performed in Stata 16.

Group 1 (F. G., N. A., R. P., L. W., I. O.) were tasked with assessing which titles and abstracts to pull full text for. Members of Group 1 were split into pairs and the titles and abstracts were divided into equal parts for each pair to review. Once the final set of abstracts was agreed upon, full text was linked to each article. Ahead of the full text review, a sample of 10 randomly selected articles was jointly reviewed by authors (S. P., B. M., E. K. C.) and any disagreements were discussed to establish that interpretation of the content of each article was consistent. Subsequently, Group 2 (A. S., G. S., A. P., and J. S.) were similarly split into pairs and allocated portions of the full text articles to determine our final list of included studies. Where possible, online appendices, addendums and errata were identified and reviewed to determine inclusion. At both stages of screening, a third team reviewer (E. K. C.) settled disagreements regarding study inclusion.

#### 4.3.2 Data extraction and management

Data extraction, along with intervention grouping, was completed by three reviewers (A. S., G. S., and E. K.C.) through an online tool (Google Forms). The complete tool can be found in Appendix Sections D–F. The data extraction form was designed in consultation with the Cochrane Handbook (Higgins & Green, 2011) and adapted from Psaki et al. (2019) and Mensch et al. (2019). Three reviewers (A. S., G. S., E. K. C.) divided the included papers into three groups, each taking one group for full data extraction. After full data extraction was completed for each paper by one reviewer, they rotated groups, and a second reviewer (A. S., G. S., E. K. C.) checked each paper's extracted data for errors. When errors arose, the two reviewers handling each paper (i.e., the reviewer who extracted the data and the reviewer who checked it) met to agree on how to resolve the error.

#### 4.3.3 Assessment of risk of bias in included studies

While RCTs are generally considered the gold standard for identifying causal impact, even they may be subject to threats to validity (e.g., differential loss to follow‐up and selective reporting of outcomes). Therefore, we performed an assessment of risk of bias in both experimental and quasi‐experimental studies adapted from RoB 2 (Higgins et al., 2016) for randomized studies and ROBINS‐I (Sterne et al., 2016) for non‐randomized studies. In addition to using the full ROBINS‐I tool for non‐randomized studies, we also added questions on methods‐specific criteria from Psaki et al. (2019), which were adapted from Baird et al. (2013). Risk of bias was assessed by two reviewers (A. S. and G. S.). A third reviewer (E. K. C.) assisted in resolving any disputes. The full assessment of risk of bias tool is presented in Supporting Information Appendix Section B. The relevant section of the risk of bias assessment tool was applied to each included study at the time of data extraction.

#### 4.3.4 Measures of treatment effect

We extracted information from quantitative models that reported on our primary outcomes of interest. This included measures such as unstandardized regression coefficients, beta coefficients, odds ratios, and *t* statistics, among others. Typically, most systematic reviews that contrast groups compute standardized mean differences if the outcome is continuous and odds ratios if the outcome is dichotomous (Lipsey & Wilson, 2001). However, given the complex estimation methods used in included studies, we instead chose to convert effect sizes to partial correlation coefficients. In some cases, for example, linear models with a continuous or dichotomous education exposure, conversion was straightforward. But other conversions were more complex. See Supporting Information Appendix Section D for a more detailed description of the computation of partial correlations.

Partial correlations represent the relationship between two variables, controlling for (i.e., “partialling out”) covariates, and range in value from −1 to 1 (Aloe, 2014; Aloe & Thompson, 2013). Due to the similarities in the estimating equations underlying partial correlations and standardized regression coefficients (*β*), we can interpret the size of the effect as the standard deviation change in the dependent variable. Drawing on Cohen's (1988) conventions for interpreting effect sizes, correlation effect sizes ≤0.10 are considered small, values of 0.25 are considered medium, and values ≥0.40 are considered large. In the case of this study, which reports partial correlation coefficients that are adjusted for a set of key covariates, we might expect the magnitude to be somewhat smaller than the guidelines for bivariate correlations. Corresponding 90% confidence intervals (CIs) were calculated for all outcomes as many authors, given the conventions in the economics literature, use *α* = .10.

#### 4.3.5 Criteria for determination of independent findings

In cases where a singular study provided results on more than one of our outcomes of interest, we presented each result separately. Similarly, if a singular study presented several measures for the same outcome, all results are presented for completeness, but are grouped by comparability of the effect size to other effect sizes, as categorized above. In addition, where multiple results for the same outcome of interest measured in the same way were presented within the same paper but were the result of different models/subgroup analyses, one effect size was reported based on the authors' indication that the effect was one of the primary results of the study. If multiple articles were identified that reported on the results from the same intervention, drew from the same study population and reported on the same or very similar outcomes, we reported the effects from the earliest published article or the longest term follow‐up (Willson, 2018). In cases where the publication date of the earliest published article and the longest‐term follow‐up were in conflict, we included the longest‐term follow‐up among our reported results.

#### 4.3.6 Unit of analysis issues

We followed the Cochrane Handbook recommendations for studies that do not correct for one or more types of unit of analysis errors. The following is a summary of the strategies we employed to treat the results subject to various manifestations of unit of analysis errors.

- Repeated observations on participants
  o If data from multiple follow‐up rounds were reported, we chose the follow‐up results that were furthest in time from the time of implementation.
- Multiple intervention groups
  o For studies with multiple intervention groups that are comparable, we reported the results of each arm separately, as long as any different components between each arm address a gender‐related barrier to girls' education.

Studies with multiple intervention groups are noted in Table 1, along with notes if an arm was excluded and the rationale. Only two studies (Bagby et al., 2017; Kazianga et al., 2012; Kazianga et al., 2019) reported on repeated observations on participants. In both cases, we reported the longest‐term follow‐up available for any primary outcomes. Other strategies that we outlined in our protocol are not listed as we did not identify studies with those unit of analysis issues. The full list of strategies can be found in the protocol (Chuang et al., 2019).

#### 4.3.7 Assessment of reporting biases

We did not find a sufficient number of studies to run quantitative analyses on reporting bias (e.g., funnel plots, trim‐and‐fill plots, tests for meta bias) for any given barrier and outcome based on our criteria for meta‐analyses. Further details about the criteria to conduct meta‐analyses can be found below.

#### 4.3.8 Data synthesis

##### Analysis of primary outcomes

We did not identify enough studies to run meta‐analyses on any given primary outcome. Interventions/exposures must have adhered to the following criteria to be grouped together in meta‐analyses:

- The intervention targets at least 1 common barrier to girls' schooling,
- The level of implementation of the intervention was the same (individual, household, school, hospital/clinic, other community‐level, other), and
- Studies could only be either experimental or quasi‐experimental, that is, an experimental study could not be grouped with a quasi‐experimental study, and
- Measurement of the reported outcome was the same across all studies in the group.

Given the issues with meta‐analyses of interventions in the social sciences (e.g., the interventions were not exact replications of each other, models and model specifications differed, inclusion criteria for studies were not the same), we opted for our cutoff of the number of studies included in each meta‐analysis be three instead of the usually suggested two to improve the inferential interpretation of any overall effects (Valentine et al., 2010). Based on this cutoff, as well as our other criteria for inclusion of studies in meta‐analysis, we did not find results from enough studies to include in meta‐analyses.

However, we constructed forest plots to compare effect sizes visually. We also developed a descriptive summary of results based on the frequency of different results within each barrier group. The main characteristics of the reported effects we focus on are whether the effects are in the expected direction (e.g., the expected direction is negative if the outcome equals to 1 if the student dropped out) and significance at *p* < .10. We also consider the relative size of each effect, using benchmarks for education interventions suggested by Kraft to categorize effects of less that 0.05 SD as small; 0.05 to <0.20 as medium; and 0.20 or greater as large (Kraft, 2019).

For the purpose of summarizing quantitative results by barrier, we used the GRADE approach adapted for narrative syntheses (Murad et al., 2017). This approach simultaneously considers several factors, including methodological limitations of the studies, how directly the study measured each type of intervention, imprecision, how consistent the findings were across studies, publication bias, and size and direction of effect. For each barrier and outcome combination (e.g., interventions designed to address Barrier 1, effects on grade attainment), we include a rating about the certainty of the evidence in the GRADE table, reflecting these considerations. Therefore, the certainty of the evidence reflects the number and quality of studies, directness of the evidence, and consistency of results rather than the direction of the effects.

To detect effects at the most granular level, we examined the extent to which a group of interventions (e.g., those designed to address inadequate water and sanitation) affected each outcome (e.g., completion of primary school), and ranked these pairings using the GRADE assessment. This resulted in an important granular perspective, but a very small number of studies for every barrier‐outcome pairing. To synthesize these in a more meaningful way, we combined the outcomes into two groups—those related to enrolment/attainment or academic skills—to capture overall patterns. Based on these aggregated GRADE rankings, we assigned the following categories with regard to the effectiveness of interventions designed to address each barrier:

- *Effective*: Multiple studies (four or more) directly measured the intervention approach and found consistently that this intervention improves education outcomes (i.e., enrolment/attainment, academic skills, or both) for girls.
- *Promising*: A few studies (two or more) directly measured the intervention approach and found that this approach improves education outcomes for girls, although there might be variation in findings.
- *More Research Needed*: Existing evidence either comes from multicomponent studies that are unable to isolate the effects of this intervention (findings are indirect), from direct studies with widely varying results (findings are inconsistent), or too few studies have been conducted.
- *Ineffective*: Multiple studies (four or more) directly measured the intervention approach and found consistently that this intervention does not improve education outcomes for girls (i.e., the intervention has no effect on education outcomes).
- *Unknown*: No rigorous studies to address the barrier have been conducted.

Note that these are meant to represent categories of relative effectiveness, rather than clear cut‐offs, to provide a metric for comparisons between intervention types. We supplement this quantitative approach with a more in‐depth narrative analysis of the studies' findings, including differences across study arms, and a focus on the evidence from studies that most directly answered our research questions.

#### 4.3.9 Subgroup analysis and investigation of heterogeneity

As we did not identify enough studies to run meta‐analyses, we in turn could not conduct moderator analyses using meta‐regressions. We attempted to narratively describe the potential sources of variability of results based on various characteristics of the studies, such as whether interventions were single or multicomponent, the types of components employed, and methodology (e.g., experimental vs. quasi‐experimental).

#### 4.3.10 Dealing with missing data

Where data were missing that could determine the inclusion eligibility of a study, such as interventions that may fall in the “Other” category, or effect size conversion, reviewers contacted the study authors to request the relevant information; three attempts to contact the authors were made within 1 month. If the authors did not respond or did not provide the relevant information within 1 month of the first date of contact, then the study would have been excluded from quantitative synthesis but included in narrative synthesis. Fortunately, all authors of included studies responded to requests for information.

## 5 RESULTS

### 5.1 Description of studies

#### 5.1.1 Results of the search

**Figure 3:**
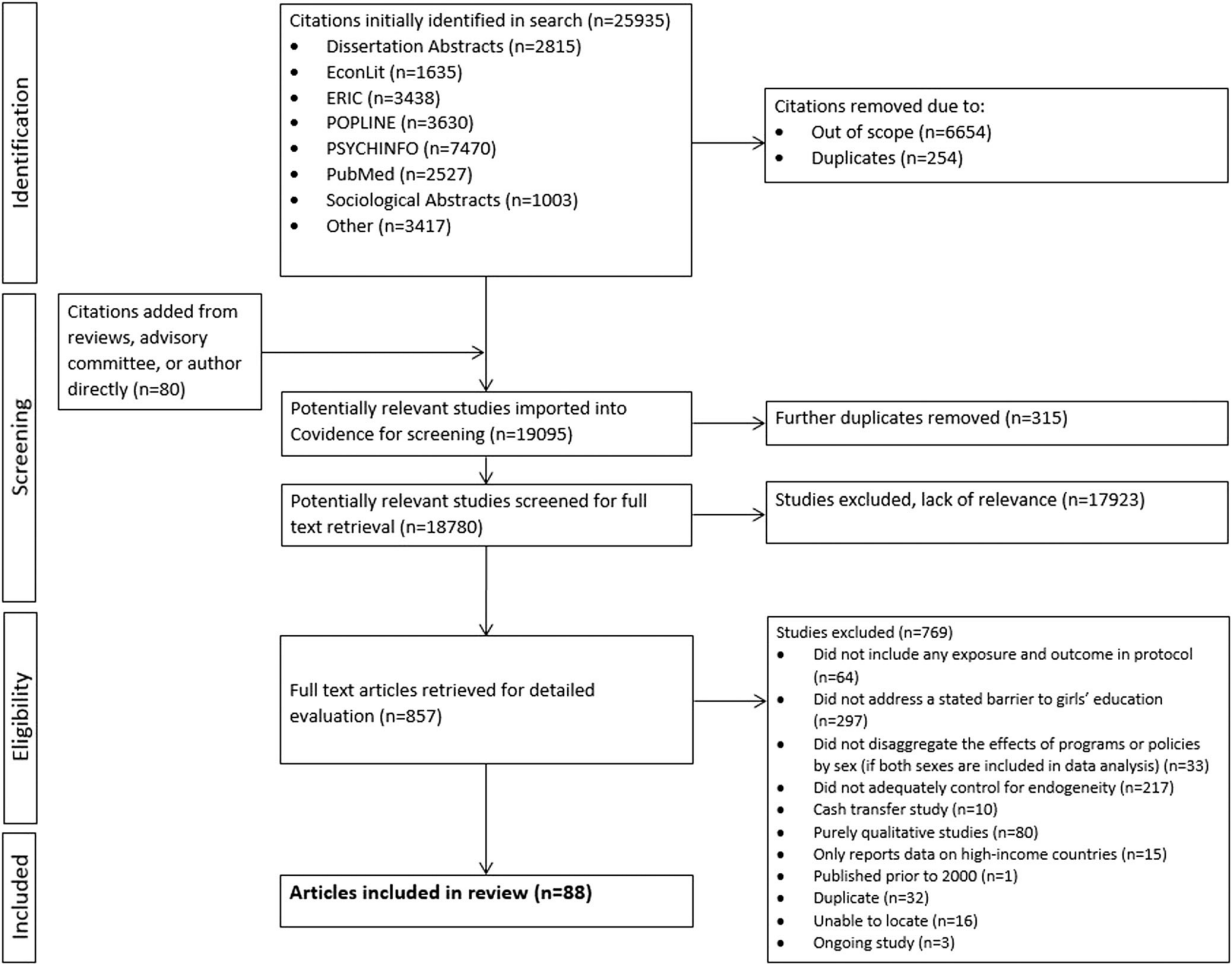
PRISMA diagram

The PRISMA flow diagram (Figure 3) indicates the number of citations initially identified (*N* = 25,935) for the full systematic review though our database search, detailed in the Methodology section. There were a number of studies (*n* = 80) that were later suggested by our advisory group, authors of included papers, or through literature review searches. Of the articles initially identified either through our search or through the independent identification of additional papers, 18,780 were screened for relevance based on titles and abstracts, and 857 went through full text review. Ultimately, results from 82 studies were included in the review from a total of 88 papers (a few studies presented results in more than one paper).

#### 5.1.2 Included studies

Of the 82 included studies (see Table 1), 41 employed an experimental design, and 41 utilized quasi‐experimental methods. For one study (Benshaul‐Tolonen et al., 2019; Phillips‐Howard et al., 2016) we draw on the findings of one journal article and one working paper. Among the rest of the studies, about half of the publications were working papers (*n* = 42), 40% were journal articles (*n* = 35), 7% were reports (*n *= 6) and 3% were dissertations (*n* = 3). Almost all (80/82) studies provided girl‐specific effects, 29% (*n *= 24) reported gender‐differential effects of interventions and exposures, with 28% (23/82) reporting both girl‐specific and gender‐differential effects for girls relative to boys. Just over half of the studies were conducted in Sub‐Saharan Africa (*n *= 43), followed by South Asia (*n* = 24). A total of 15 countries in Sub‐Saharan Africa were represented, of which the most frequently represented were Kenya (*n* = 9), Uganda (*n *= 7), Ethiopia (*n *= 4), Zambia (*n* = 4), Zimbabwe (*n *= 4), and Ghana (*n* = 3). The majority of studies in South Asia were based in India (14), followed by Bangladesh (6), Pakistan (3) and Nepal (1). The rest of the studies were conducted in Latin America and the Caribbean (*n* = 5), Central Asia (*n* = 1), South East Asia (*n* = 3), East Asia (*n* = 4), and the Middle East and North Africa regions (*n* = 2). All East Asia studies were set in China. The earliest included study was from 2004, with the most recent study was published in 2020.

One characteristic of included studies that we expected to find challenging analytically is the issue of multicomponent programs. Indeed, many studies evaluate programs that include multiple components (see descriptions in Table 1). For example, an initiative may include school construction as well as teacher training. Thus, many of the studies are included under more than one barrier (see Tables  5.1 through 5.18). We explore the issues with synthesizing the results of multicomponent interventions in Sections Data Collection and Analysis, 5 and Results, 6.

**Table 5.1:** Included studies by barrier(s), barrier 1: lack of support for girls' education

Author (year)	1	2	3	4	5	6	7	8	9	10	11	12	13	14	15	16	17	18	Total # barriers addressed
Bagby et al. (2017)	*●*				*●*		*●*						*●*	*●*			*●*		*6*
Buchmann et al. (2016)	*●*	*●*	*●*								*●*							*●*	*5*
Delavallade et al. (2014)	*●*							*●*											*2*
Hungi and Ngware (2017)	*●*							*●*			*●*					*●*			*4*
Kazianga et al. (2012); Kazianga et al. (2019)	*●*		*●*		*●*								*●*	*●*			*●*	*●*	*7*
McCadden (2015)	*●*														*●*				*2*
Meller and Litschig (2015)	*●*		*●*		*●*		*●*	*●*	*●*	*●*	*●*		*●*	*●*					*10*
Mensch et al. (2019)	*●*		*●*			*●*	*●*	*●*			*●*								*6*
Özler et al. (2020)	*●*					*●*					*●*								*3*
Total	9	*1*	*4*	*0*	*3*	*2*	*3*	*4*	*1*	*1*	*5*	*0*	*3*	*3*	*1*	*1*	*2*	*2*	

**Table 5.2:** Included studies by barrier(s), barrier 2: child marriage and adolescent pregnancy

Author (year)	1	2	3	4	5	6	7	8	9	10	11	12	13	14	15	16	17	18	Total # barriers addressed
Bandiera et al. (2014)		●	●			●					●								4
Bandiera et al. (2019)		●	●			●					●								4
Buchmann et al. (2016)	●	●	●								●							●	5
Edmonds et al. (2016)		●						●			●								3
Total	1	4	3	0	0	2	0	1	0	0	4	0	0	0	0	0	0	1	

**Table 5.3:** Included studies by barrier(s), barrier 3: lack of information on returns to education/alternative roles for women

Author (year)	1	2	3	4	5	6	7	8	9	10	11	12	13	14	15	16	17	18	Total # barriers addressed
Ashraf et al. (2018)			*●*			*●*					*●*						*●*	*●*	*5*
Austrian et al. (2020)			*●*			*●*					*●*								*3*
Avitabile and de Hoyos (2018)			*●*																*1*
Bandiera et al. (2014)		*●*	*●*			*●*					*●*								*4*
Bandiera et al. (2019)		*●*	*●*			*●*					*●*								*4*
Buchmann et al. (2016)	*●*	*●*	*●*								*●*							*●*	*5*
Heath and Mobarak (2014)			*●*																*1*
Jensen and Oster (2007)			*●*																*1*
Jensen (2012)			*●*																*1*
Kazianga et al. (2012); Kazianga et al. (2019)	*●*		*●*		*●*								*●*	*●*			*●*	*●*	*7*
Meller and Litschig (2015)	*●*		*●*		*●*		*●*	*●*	*●*	*●*	*●*		*●*	*●*					*10*
Mensch et al. (2019)	*●*		*●*			*●*	*●*	*●*			*●*								*6*
Stark et al. (2018)			*●*			*●*					*●*								*3*
Total	*4*	*3*	*13*	0	*2*	*6*	*2*	*2*	*1*	*1*	*8*	0	*2*	*2*	0	0	*2*	*3*	

**Table 5.5:** Included studies by barrier(s), barrier 5: gender insensitive school environment

Author (year)	1	2	3	4	5	6	7	8	9	10	11	12	13	14	15	16	17	18	Total # barriers addressed
Aber et al. (2017)					*●*														*1*
Asadullah and Chaudhury (2013)					*●*									*●*					*2*
Bagby et al. (2017)	*●*				*●*		*●*						*●*	*●*			*●*		*6*
Eble and Hu (2019)					*●*														*1*
Kazianga et al. (2012); Kazianga et al. (2019)	*●*		*●*		*●*								*●*	*●*			*●*	*●*	*7*
Meller and Litschig (2015)	*●*		*●*		*●*		*●*	*●*	*●*	*●*	*●*		*●*	*●*					*10*
Morrell et al. (2014)					*●*	*●*		*●*			*●*								*4*
Muralidharan and Sheth (2013)					*●*														*1*
Sukontamarn (2005)					●									●					2
Total	*3*	0	*2*	0	*9*	*1*	*2*	*2*	*1*	*1*	*2*	0	*3*	*5*	0	0	*2*	*1*	

**Table 5.6:** Included studies by barrier(s), barrier 6: lack of safe spaces and social connections

Author (year)	1	2	3	4	5	6	7	8	9	10	11	12	13	14	15	16	17	18	Total # barriers addressed
Ashraf et al. (2018)			*●*			*●*					*●*						*●*	*●*	*5*
Austrian et al. (2020)			*●*			*●*					*●*								*3*
Bandiera et al. (2014)		*●*	*●*			*●*					*●*								*4*
Bandiera et al. (2019)		*●*	*●*			*●*					*●*								*4*
Hahn et al. (2016)						*●*													*1*
Lu and Anderson (2015)						*●*													*1*
Mensch et al. (2019)	*●*		*●*			*●*	*●*	*●*			*●*								*6*
Morrell et al. (2014)					*●*	*●*		*●*			*●*								*4*
Özler et al. (2020)	*●*					*●*					*●*								*3*
Stark et al. (2018)			*●*			*●*					*●*								*3*
Total	*2*	*2*	*6*	0	*1*	*10*	*1*	*2*	0	0	*8*	0	0	0	0	0	*1*	*1*	

**Table 5.7:** Included studies by barrier(s), barrier 7: lack of teaching materials and supplies

Author (year)	1	2	3	4	5	6	7	8	9	10	11	12	13	14	15	16	17	18	Total # barriers addressed
Bagby et al. (2017)	*●*				*●*		*●*						*●*	*●*			*●*		*6*
Burde and Linden (2009)							*●*							*●*			*●*		*3*
Lakshminarayana et al. (2013)							*●*	*●*									*●*		*3*
Meller and Litschig (2015)	*●*		*●*		*●*		*●*	*●*	*●*	*●*	*●*		*●*	*●*					*10*
Mensch et al. (2019)	*●*		*●*			*●*	*●*	*●*			*●*								*6*
Total	3	0	2	0	2	1	5	3	1	1	2	0	2	3	0	0	0	0	

**Table 5.8:** Included studies by barrier(s), barrier 8: insufficient academic support

Author (year)	1	2	3	4	5	6	7	8	9	10	11	12	13	14	15	16	17	18	Total # barriers addressed
Beg et al. (2018)								*●*											*1*
Cho et al. (2019)								*●*								*●*	*●*		*3*
Delavallade et al. (2014)	*●*							*●*											*2*
Edmonds et al. (2016)		*●*						*●*			*●*								*3*
Hallfors et al. (2011); Iritani et al. (2016)								*●*				*●*	*●*			*●*	*●*		*5*
Hungi and Ngware (2017)	*●*							*●*			*●*					*●*			*4*
Lakshminarayana et al. (2013)							*●*	*●*									*●*		*3*
Meller and Litschig (2015)	*●*		*●*		*●*		*●*	*●*	*●*	*●*	*●*		*●*	*●*					*10*
Mensch et al. (2019)	*●*		*●*			*●*	*●*	*●*			*●*								*6*
Morrell et al. (2014)					*●*	*●*		*●*			*●*								*4*
Muralidharan et al. (2016)								*●*											*1*
Okurut (2015); Okurut (2018)								*●*							*●*				*2*
Yang et al. (2013)								*●*											*1*
Total	4	1	2	0	2	2	3	13	1	1	5	1	2	1	1	3	3	0	

**Table 5.9:** Included studies by barrier(s), barrier 9: inadequate sports programs for girls

Author (year)	1	2	3	4	5	6	7	8	9	10	11	12	13	14	15	16	17	18	Total # barriers addressed
Meller and Litschig (2015)	*1*		*1*		*1*		*1*	*1*	*1*	*1*	*1*		*1*	*1*					*10*
*Total*	*1*	*0*	*1*	*0*	*1*	*0*	*1*	*1*	*1*	*1*	*1*	*0*	*1*	*1*	*0*	*0*	*0*	*0*	

**Table 5.10:** Included studies by barrier(s), barrier 10: inadequate health and childcare services

Author (year)	1	2	3	4	5	6	7	8	9	10	11	12	13	14	15	16	17	18	Total # barriers addressed
Meller and Litschig (2015)	*1*		*1*		*1*		*1*	*1*	*1*	*1*	*1*		*1*	*1*					*10*
Total	1	0	1	0	1	0	1	1	1	1	1	0	1	1	0	0	0	0	

**Table 5.11:** Included studies by barrier(s), barrier 11: inadequate life skills

Author (year)	1	2	3	4	5	6	7	8	9	10	11	12	13	14	15	16	17	18	Total # barriers addressed
Ashraf et al. (2018)			*●*			*●*					*●*						*●*	*●*	5
Austrian et al. (2020)			*●*			*●*					*●*								3
Bandiera et al. (2014)		*●*	*●*			*●*					*●*								4
Bandiera et al. (2019)		*●*	*●*			*●*					*●*								4
Buchmann et al. (2016)	*●*	*●*	*●*								*●*							*●*	5
Carney et al. (2019)											*●*								1
Duflo et al. (2014)											*●*						*●*		2
Edmonds et al. (2016)		*●*						*●*			*●*								3
Hungi and Ngware (2017)	*●*							*●*			*●*					*●*			4
Johnston and Ksoll (2017)											*●*			*●*					2
Meller and Litschig (2015)	*●*		*●*		*●*		*●*	*●*	*●*	*●*	*●*		*●*	*●*					10
Mensch et al. (2019)	*●*		*●*			*●*	*●*	*●*			*●*								6
Morrell et al. (2014)					*●*	*●*		*●*			*●*								4
Özler et al. (2020)	*●*					*●*					*●*								3
Stark et al. (2018)			*●*			*●*					*●*								3
**Total**	**5**	**4**	**8**	**0**	**2**	**8**	**2**	**5**	**1**	**1**	**15**	**0**	**1**	**2**	**0**	**1**	**2**	**2**	

**Table 5.12:** Included studies by barrier(s), barrier 12: inadequate menstrual hygiene management

Author (year)	1	2	3	4	5	6	7	8	9	10	11	12	13	14	15	16	17	18	Total # barriers addressed
Benshaul‐Tolonen et al. (2019); Phillips‐Howard et al. (2016)												*●*							1
Hallfors et al. (2011); Iritani et al. (2016)								*●*				*●*	*●*			*●*	*●*		5
Oster and Thornton (2009)												*●*							1
Wilson et al. (2012)												*●*							1
Total	0	0	0	0	0	0	0	1	0	0	0	4	1	0	0	1	1	0	

**Table 5.13:** Included studies by barrier(s), barrier 13: lack of water and sanitation

Author (year)	1	2	3	4	5	6	7	8	9	10	11	12	13	14	15	16	17	18	Total # barriers addressed
Adukia (2016)													*●*						1
Bagby et al. (2017)	*●*				*●*		*●*						*●*	*●*			*●*		6
Caruso et al. (2014)													*●*						1
Freeman et al. (2012); Garn et al. (2013)													*●*						1
Hallfors et al. (2011); Iritani et al. (2016)								*●*				*●*	*●*			*●*	*●*		5
Kazianga et al. (2012); Kazianga et al. (2019)	*●*		*●*		*●*								*●*	*●*			*●*	*●*	7
Meller and Litschig (2015)	*●*		*●*		*●*		*●*	*●*	*●*	*●*	*●*		*●*	*●*					10
Total	3	0	2	0	3	0	2	2	1	1	1	1	7	3	0	1	3	1	

**Table 5.14:** Included studies by barrier(s), barrier 14: inadequate school access

Author (year)	1	2	3	4	5	6	7	8	9	10	11	12	13	14	15	16	17	18	Total # barriers addressed
Agüero and Bharadwaj (2014)														*●*	*●*	*●*			3
Akresh et al. (2018)														*●*					1
Andalón et al. (2014)														*●*	*●*				2
Asadullah and Chaudhury (2013)					*●*									*●*					2
Bagby et al. (2017)	*●*				*●*		*●*						*●*	*●*			*●*		6
Burde and Linden (2009)							*●*							*●*			*●*		3
Chatterjee (2017)														*●*					1
Chicoine (2016)														*●*	*●*	*●*			3
Chin (2005)														*●*					1
Datta Gupta et al. (2018)														*●*			*●*	*●*	3
De Neve and Subramanian (2017)														*●*	*●*	*●*			3
Erten and Keskin (2018)														*●*	*●*				2
Grépin and Bharadwaj (2015)														*●*	*●*	*●*			3
Güneş (2016)														*●*	*●*	*●*			3
Jacoby and Mansuri (2011)														*●*					1
Johnston and Ksoll (2017)											*●*			*●*					2
Kazianga et al. (2012); Kazianga et al. ( 2019)	*●*		*●*		*●*								*●*	*●*			*●*	*●*	7
Meller and Litschig (2015)	*●*		*●*		*●*		*●*	*●*	*●*	*●*	*●*		*●*	*●*					10
Muralidharan and Prakash (2013)														*●*					1
Osili and Long (2008)														*●*		*●*			2
Sukontamarn (2005)					*●*									*●*					2
Tequame and Tirivayi (2015)														*●*	*●*				2
Yamauchi and Liu (2011a, 2011b)														*●*			*●*		2
Total	3	0	2	0	5	0	3	1	1	1	2	0	3	23	8	6	5	2	

**Table 5.15:** Included studies by barrier(s), barrier 15: poor policy/legal environment

Author (year)	1	2	3	4	5	6	7	8	9	10	11	12	13	14	15	16	17	18	Total # barriers addressed
Agüero and Bharadwaj (2014)														*●*	*●*	*●*			3
Andalón et al. (2014)														*●*	*●*				2
Argaw (2013)															*●*				1
Barrera‐Osorio et al. (2017)															*●*	*●*			2
Chicoine (2016)														*●*	*●*	*●*			3
De Neve and Subramanian (2017)														*●*	*●*	*●*			3
Erten and Keskin (2018)														*●*	*●*				2
Grépin and Bharadwaj (2015)														*●*	*●*	*●*			3
Güneş (2016)														*●*	*●*	*●*			3
McCadden (2015)	*●*														*●*				2
Okurut (2015); Okurut (2018)								*●*							*●*				2
Tequame and Tirivayi (2015)														*●*	*●*				2
Total	1	0	0	0	0	0	0	1	0	0	0	0	0	8	12	6	0	0	

**Table 5.16:** Included studies by barrier(s), barrier 16: inability to afford tuition and fees

Author (year)	1	2	3	4	5	6	7	8	9	10	11	12	13	14	15	16	17	18	Total # barriers addressed
Adelman et al. (2017)																*●*			*1*
Agüero and Bharadwaj (2014)														*●*	*●*	*●*			*3*
Barrera‐Osorio et al. (2017)															*●*	*●*			*2*
Blimpo et al. (2016)																*●*			*1*
Chicoine (2016)														*●*	*●*	*●*			*3*
Cho et al. (2019)								*●*								*●*	*●*		*3*
Chyi and Zhou (2010)																*●*	*●*		*2*
De Neve and Subramanian (2017)														*●*	*●*	*●*			*3*
Duflo et al. (2019)																*●*			*1*
Grant (2015)																*●*			*1*
Grépin and Bharadwaj (2015)														*●*	*●*	*●*			*3*
Grogan (2009)																*●*			*1*
Güneş (2016)														*●*	*●*	*●*			*3*
Hallfors et al. (2011); Iritani et al. (2016)								*●*				*●*	*●*			*●*	*●*		*5*
Hermida (2014)																*●*			*1*
Hungi and Ngware (2017)	*●*							*●*			*●*					*●*			*4*
Keats (2018)																*●*			*1*
Lucas and Mbiti (2010)																*●*			*1*
Makate (2016)																*●*			*1*
Mbiti et al. (2019)																*●*			*1*
Osili and Long (2008)														*●*		*●*			*2*
*Totals*	*1*	*0*	*0*	*0*	*0*	*0*	*0*	*3*	*0*	*0*	*1*	*1*	*1*	*6*	*6*	*21*	*3*	*0*	

**Table 5.17:** Included studies by barrier(s), barrier 17: inability to afford school materials

Author (year)	1	2	3	4	5	6	7	8	9	10	11	12	13	14	15	16	17	18	Total # barriers addressed
Ashraf et al. (2018)			*●*			*●*					*●*						*●*	*●*	5
Bagby et al. (2017)	*●*				*●*		*●*						*●*	*●*			*●*		6
Burde and Linden (2009)							*●*							*●*			*●*		3
Cho et al. (2019)								*●*								*●*	*●*		3
Chyi and Zhou (2010)																*●*	*●*		2
Datta Gupta et al. (2018)														*●*			*●*	*●*	3
Duflo et al. (2014)											*●*						*●*		2
Evans and Ngatia (2018)																	*●*		1
Giordono and Pugatch (2017)																	*●*		1
Hallfors et al. (2011); Iritani et al. (2016)								*●*				*●*	*●*			*●*	*●*		5
Hidalgo et al. (2010)																	*●*		1
Kazianga et al. (2012); Kazianga et al. (2019)	*●*		*●*		*●*								*●*	*●*			*●*	*●*	7
Lakshminarayana et al. (2013)							*●*	*●*									*●*		3
Yamauchi and Liu (2011a, 2011b)														*●*			*●*		2
Total	2	0	2	0	2	1	3	3	0	0	2	1	3	5	0	3	14	3	

**Table 5.18:** Included studies by barrier(s), barrier 18: lack of adequate food

Author (year)	1	2	3	4	5	6	7	8	9	10	11	12	13	14	15	16	17	18	Total # barriers addressed
Ashraf et al. (2018)			*●*			*●*					*●*						*●*	*●*	5
Aurino et al. (2018)																		*●*	1
Buchmann et al. (2016)	*●*	*●*	*●*								*●*							*●*	5
Buttenheim et al. (2011)																		*●*	1
Datta Gupta et al. (2018)														*●*			*●*	*●*	3
Kaur (2017)																		*●*	1
Kazianga et al. (2012); Kazianga et al. (2019)	*●*		*●*		*●*								*●*	*●*			*●*	*●*	7
Kazianga et al. (2009)																		*●*	1
Lehrer (2010)																		*●*	1
Sukontamarn (2013)																		*●*	1
Total	2	1	3	0	1	1	0	0	0	0	2	0	1	2	0	0	3	10	

#### 5.1.3 Excluded studies

During the initial database search, 6654 studies were excluded because they were out of scope based on our inclusion criteria, and 254 studies were removed as duplicates. A further 315 duplicate studies were removed when the results of the database search were imported into Covidence. At the Title and Abstract Screening phase, we excluded 17,923 studies due to lack of relevance to the review, based on our inclusion criteria. Seven hundred sixty nine studies were excluded at the Full Text Review stage, of which the majority did not address a barrier to girls' education (*n* = 297) or did not use a quantitative method that adequately controlled for endogeneity (*n* = 217). 80 studies, while gender‐informed, were purely qualitative, 64 studies did not include an exposure or outcome that was outlined in the protocol, 33 studies did not disaggregate the effects of the programs or policies by sex, 15 studies were conducted in high‐income countries and 10 were purely cash transfer studies. We found and excluded 32 further duplicates, one study published before 2000 and three ongoing studies. We were unable to locate the full text for 16 studies.

### 5.2 Risk of bias in included studies

#### 5.2.1 Experimental studies

Of the 41 experimental studies, all but five were judged to have low risk of bias (Table 3). Four studies (Aber et al., 2017, Austrian et al., 2020, Cho et al., 2019, Freeman et al., 2012; Garn et al., 2013) were determined to have some concerns, and one (Wilson et al., 2012) was judged to have high risk of bias based on the tool we utilized. The most common potential sources of bias among these included studies were from issues with randomization, deviations in assignment and selection of the reported result. The main cause for concern related to randomization and assignment was the lack of blinding and concealment of intervention assignment among the participants and implementors throughout the programs. This is typical of the field at large, as any non‐laboratory‐based experimental social science studies are not blinded at either the participant or investigator level, if they report on blinding at all (Deaton & Cartwright, 2018). As for bias due to selection of reported results, the primary reason for the relatively higher numbers of papers with “some concerns” with bias is due to the lack of prespecified plans for analysis of the trial(s). This is again typical of social science studies—the publication of pre‐analysis plans or study protocols before trial implementation, even for RCTs, is uncommon, though there has been a recent push among researchers to change this norm (Asendorpf et al., 2016; Lupia & Elman, 2014; Miguel et al., 2014).

**Table 3:** Risk of bias, experimental studies

Author (year)	RoB 2: bias from randomization process	RoB 2: bias from deviations in assignment from intended interventions	RoB 2: bias from missing outcome data	RoB 2: bias due to measurement of outcome	RoB 2: bias due to selection of reported result	Overall risk of bias
Aber et al. (2017)	Some concerns	Low	Low	Low	Some concerns	Some concerns
Adelman et al. (2017)	Low	Low	Low	Low	Some concerns	Low
Ashraf et al. (2018)	Low	Low	Low	Low	Low	Low
Aurino et al. (2018)	Low	Low	Low	Low	Low	Low
Austrian et al. (2020)	Some concerns	Some concerns	Some concerns	Low	Low	Some concerns
Avitabile and de Hoyos (2018)	Low	Low	Low	Low	Low	Low
Bagby et al. (2017)	Low	Low	Low	Low	Low	Low
Bandiera et al. (2014)	Low	Low	Low	Low	Low	Low
Bandiera et al. (2019)	Low	Low	Low	Low	Low	Low
Barrera‐Osorio et al. (2017)	Low	Low	Low	Low	Some concerns	Low
Beg et al. (2018)	Low	Some concerns	Low	Low	Low	Low
Benshaul‐Tolonen et al. (2019); Phillips‐Howard et al. (2016)	Low	Low	Low	Low	Low	Low
Buchmann et al. (2016)	Low	Low	Low	Low	Low	Low
Burde and Linden (2009)	Low	Low	Low	Low	Low	Low
Carney et al. (2019)	Low	Low	Low	Low	Low	Low
Caruso et al. (2014)	Low	Low	Low	Low	Low	Low
Cho et al. (2019)	Some concerns	Some concerns	Low	Low	Some concerns	Some concerns
Delavallade et al. (2014)	Low	Low	Low	Low	Some concerns	Low
Duflo et al. (2014)	Low	Low	Low	Low	Some concerns	Low
Duflo et al. (2019)	Low	Low	Low	Some concerns	Low	Low
Eble and Hu (2019)	Some concerns	Low	Low	Low	Low	Low
Edmonds et al. (2016)	Low	Low	Low	Low	Low	Low
Evans and Ngatia (2018)	Low	Low	Low	Low	Low	Low
Freeman et al. (2012); Garn et al. (2013)	Low	Low	Some concerns	Some concerns	Low	Some concerns
Hahn et al. (2016)	Low	Low	Some concerns	Low	Low	Low
Hallfors et al. (2011); Iritani et al. (2016)	Low	Low	Some concerns	Low	Low	Low
Hidalgo et al. (2010)	Low	Some concerns	Low	Low	Low	Low
Jensen (2012)	Low	Some concerns	Low	Low	Low	Low
Johnston and Ksoll (2017)	Low	Low	Low	Low	Low	Low
Kazianga et al. (2009)	Low	Low	Some concerns	Low	Low	Low
Lakshminarayana et al. (2013)	Low	Low	Low	Low	Low	Low
Lehrer (2010)	Low	Low	Some concerns	Low	Low	Low
Lu and Anderson (2015)	Some concerns	Low	Low	Low	Low	Low
Mbiti et al. (2019)	Low	Low	Low	Low	Low	Low
Mensch et al. (2019)	Some concerns	Low	Low	Low	Low	Low
Muralidharan et al. (2016)	Low	Low	Low	Low	Low	Low
Oster and Thornton (2009)	Low	Some concerns	Low	Low	Low	Low
Özler et al. (2020)	Low	Low	Low	Low	Low	Low
Stark et al. (2018)	Low	Low	Low	Low	Some concerns	Low
Wilson et al. (2012)	High	Low	Low	Some concerns	Low	High
Yang et al. (2013)	Low	Low	Low	Low	Low	Low

#### 5.2.2 Quasi‐experimental studies

Among the 41 quasi‐experimental studies included in the review, the overall risk of bias results were more mixed than those of the experimental studies (Table 4). Twenty two (22) were determined to have low risk of bias, while 16 had some concerns and three had high risk of bias based on our criteria. The most common sources of potential bias were due to confounding, missing data and methods‐specific criteria. It is noteworthy that the majority of studies had either some concerns or high risk of bias due to missing data (*n* = 35 and *n* = 2, respectively). This is not altogether surprising given that most quasi‐experiments tended to draw on secondary data, so most of our included studies did not report on how observations missing either from data collection or within their variable(s) of interest may have affected their analyses. Focusing on bias due to confounding, 27% (*n* = 11) were found to have some concerns and 5% (*n* = 2) had high risk of bias. This was primarily due to the phrasing of the items in ROBINS‐I tool. For example, the item “Is there potential for confounding of the effect of the intervention in this study?” was far too open‐ended for our team to confidently answer “No” or “Probably No.” Because the ROBINS‐I tool was originally geared towards nonrandomized smaller‐scale interventions, the phrasing of the items was perhaps not optimized for secondary analysis of non‐randomized exposures such as policy changes. Finally, 10 studies were determined to have “some concerns” with bias due to methods‐specific criteria, with two studies having a “high” risk of bias in this area. These studies used a variety of methods including difference‐in‐differences, two stage least squares, and regression discontinuity. Concerns included lack of Hausman test and insufficient information on specifications for propensity score matching.

**Table 4:** Risk of bias, quasi‐experimental studies

Author (year)	ROBINS‐I: Bias due to confounding	ROBINS‐I: Bias in selection of participants into the study	ROBINS‐I: Bias in classification of interventions	ROBINS‐I: Bias due to deviations from intended interventions	ROBINS‐I: Bias due to missing data	ROBINS‐I: Bias in measurement of outcomes	ROBINS‐I: Bias in selection of the reported result	Methods‐specific criteria	Overall Risk of Bias
Adukia (2016)	Low	Low	Low	Low	Low	Low	Low	High	Some concerns
Agüero and Bharadwaj (2014)	Low	Low	Low	Low	Some concerns	Low	Low	Low	Low
Akresh et al. (2018)	Low	Low	Low	Low	Some concerns	Low	Low	Low	Low
Andalon (2014)	Low	Low	Low	Low	Some concerns	Low	Low	Low	Low
Argaw (2013)	Low	Low	Low	Low	Some concerns	Low	Low	Some concerns	Some concerns
Asadullah and Chaudhury (2013)	Some concerns	Low	Low	Low	Some concerns	Low	Low	Some concerns	Some concerns
Blimpo et al. (2016)	Low	Low	Low	Low	Some concerns	Low	Low	Some concerns	Some concerns
Buttenheim et al. (2011)	Low	Low	Low	Low	Some concerns	Low	Low	Low	Low
Chatterjee (2017)	Low	Low	Low	Low	Some concerns	Low	Some concerns	Low	Low
Chicoine (2016)	Low	Low	Low	Low	Some concerns	Low	Low	Low	Low
Chin (2005)	Some concerns	Low	Low	Low	Some concerns	Low	Low	Some concerns	Some concerns
Chyi and Zhou (2010)	Low	Low	Low	Some concerns	Some concerns	Low	Some concerns	Low	Some concerns
Datta Gupta et al. (2018)	Low	Some concerns	Low	Low	Some concerns	Low	Some concerns	Low	Some concerns
De Neve and Subramanian (2017)	Low	Low	Low	Low	Some concerns	Low	Low	Low	Low
Erten and Keskin (2018)	Low	Low	Low	Low	Some concerns	Low	Low	Some concerns	Some concerns
Giordono and Pugatch (2017)	Some concerns	Low	Low	Low	Some concerns	Low	Low	Some concerns	Some concerns
Grant (2015)	Low	Low	Low	Low	Some concerns	Low	Low	Low	Low
Grépin and Bharadwaj (2015)	Low	Low	Low	Low	Some concerns	Low	Low	Low	Low
Grogan (2009)	Some concerns	Low	Low	Low	Some concerns	Low	Low	Low	Low
Güneş (2016)	Low	Low	Low	Low	Some concerns	Low	Low	Low	Low
Heath and Mobarak (2014)	Low	Low	Low	Low	Some concerns	Low	Low	Low	Low
Hermida (2014)	Low	Low	Low	Low	Some concerns	Low	Low	Low	Low
Hungi and Ngware (2017)	High	Low	Low	Low	High	Low	Low	Low	High
Jacoby and Mansuri (2011)	Some concerns	Some concerns	Low	Low	Some concerns	Low	Low	Low	Some concerns
Jensen and Oster (2007)	Some concerns	Low	Some concerns	Low	Some concerns	Low	Low	Some concerns	Some concerns
Kaur (2017)	Low	Low	Low	Some concerns	Some concerns	Low	Low	Some concerns	Some concerns
Kazianga et al. (2012); Kazianga et al. (2019)	Low	Low	Low	Low	Some concerns	Low	Low	Low	Some concerns
Keats (2018)	Some concerns	Low	Low	Low	Some concerns	Low	Low	Low	Low
Lucas and Mbiti (2010)	Some concerns	Low	Low	Low	High	Low	Low	High	High
Makate (2016)	Low	Low	Low	Low	Some concerns	Low	Low	Low	Low
McCadden (2015)	Some concerns	Low	Low	Low	Some concerns	Low	Low	Low	Low
Meller and Litschig (2015)	Some concerns	Low	Low	Low	Some concerns	Some concerns	Low	Low	Some concerns
Morrell et al. (2014)	High	Some concerns	Low	Some concerns	Some concerns	Low	Low	Low	High
Muralidharan and Prakash (2013)	Low	Low	Low	Low	Some concerns	Low	Low	Low	Low
Muralidharan and Sheth (2013)	Some concerns	Low	Low	Low	Low	Low	Low	Low	Low
Okurut (2015); Okurut (2018)	Low	Low	Low	Low	Some concerns	Low	Low	Low	Low
Osili and Long (2008)	Low	Low	Low	Low	Some concerns	Low	Low	Low	Low
Sukontamarn (2005)	Low	Low	Low	Low	Some concerns	Low	Low	Low	Low
Sukontamarn (2013)	Low	Low	Low	Low	Low	Low	Low	Low	Low
Tequame and Tirivayi (2015)	Low	Low	Low	Low	Some concerns	Low	Low	Some concerns	Some concerns
Yamauchi and Liu (2011a, 2011b)	Low	Low	Low	Low	Low	Low	Low	Some concerns	Some concerns

### 5.3 Synthesis of results

Categorizing the studies according to the gender‐related barrier(s) the intervention was designed to address was not straightforward. In describing potential disadvantages faced by girls, many authors did not specify a barrier that their interventions or exposures were addressing. In addition, the language used to explain girls' potential disadvantage often differed somewhat from the terms we employed to describe barriers. Based on the study descriptions, of the 82 studies, we determined that 44 (54%) appeared to address more than one barrier. We first provide an overview of results for all barriers and then present details for each barrier individually.

Table 2 summarizes the number of studies identified by gender‐related barrier to schooling for girls. We found at least one study for all barriers except SRGBV. For two barriers (inadequate sports programs for girls and inadequate health and childcare services) we found only one study, and for two others (child marriage and adolescent pregnancy and inadequate MHM) we found fewer than five studies. The barriers for which we found the most evidence were inadequate life skills (15 studies), inability to afford tuition and fees (21 studies) and inadequate school access (23 studies).

**Table 2:** Number of included studies by barrier

Barrier description	Total
1. Lack of support for girls' education	9
2. Child marriage and adolescent pregnancy	4
3. Lack of information on returns to education/alternative roles for women	13
4. School‐related gender‐based violence	0
5. Gender insensitive school environment	9
6. Lack of safe spaces and social connections	10
7. Lack of teaching materials and supplies	5
8. Insufficient academic support	13
9. Inadequate sports programs for girls	1
10. Inadequate health and childcare services	1
11. Inadequate life skills	15
12. Inadequate menstrual hygiene management	4
13. Lack of water and sanitation	7
14. Inadequate school access	23
15. Poor policy/legal environment	12
16. Inability to afford tuition and fees	21
17. Inability to afford school materials	14
18. Lack of adequate food	10
Total barriers addressed	171

GRADE Summary 1 contains a summary of primary results, which focuses on studies that report the effects of interventions on girls, addressing our primary research question (see Supplement Table S2 for details regarding the findings on each barrier's outcomes). The GRADE Summary 1 table presents, by each outcome measured (e.g., grade attainment, enrolment in primary school, literacy, etc.) for each barrier:

- Effect direction and size summary (the number of studies with a significant effect (*p* < .10), the direction of the effect, and its relative size (small: < 0.05; medium: 0.05 to <0.20; large: 0.20 or greater), as well as a narrative assessment of the proportion of estimated effects that were significant in the expected direction.
- Number of studies and participants (the number of experimental and quasi‐experimental studies that examined this barrier‐outcome pairing; and the total number of participants).
- Certainty in the evidence (ranking of very low, low, moderate, or high).
- GRADE ranking based on (consideration of number of studies, directness of evidence, consistency of results that led to the ranking).
- As well as a consolidated summary assessment looking across outcomes for each barrier (ranking of unknown, more research needed (either because not enough directly relevant research or because of heterogenous effects across studies), promising, or effective based on consideration of number of studies, directness of evidence, size of effects and consistency of results).

Given the heterogeneity of the interventions as well as the outcome measurements reported, this was the technique that was best suited to summarizing the information available.

GRADE Summary 2 reports on studies that included a female‐treatment interaction in addition to reporting the overall effects (boys and girls combined) of interventions or exposures on our outcomes of interest^8^ (see Table S3 for details regarding the findings on each barrier's outcomes). Both the sex variables and treatment variables were dichotomous in all cases. The inclusion of these studies, while not initially the aim of this review, provides insight not only into the effects of these interventions on girls and boys, but also the differential results of each intervention for girls relative to boys. As with GRADE Summary 1, GRADE Summary 2 reports the results across the above noted dimensions—effect direction and size; number of studies and participants; certainty in the evidence; rationale for the certainty ranking; and consolidated summary. These results help supplement the findings in GRADE Summary 1 by indicating whether the interventions were effective for children overall (girls and boys combined) and which interventions led to larger improvements for girls than boys.

In GRADE Summary 2, fewer studies reported both overall and differential effects for girls relative to boys. For three barriers—gender insensitive school environment, lack of access to school, and inability to afford school materials—four or more studies met our inclusion criteria and reported this information. Most studies and papers report significant overall effects of the interventions and programs on schooling outcomes in the expected direction. However, there are too few studies and effects in most cases to come to any conclusions about which interventions might be most effective at narrowing gender gaps, and how this varies by setting.

#### 5.3.1 Lack of support for girls' education (Barrier 1)

Overall, we find mixed results for programs that aim, in part, to address lack of support for girls' education, and very low certainty in the evidence. More research is needed to tease out the effects of this component from those of broader programs, and to understand the pathways through which increased support for girls' education might affect education outcomes.

**Figure 4.1.1:**
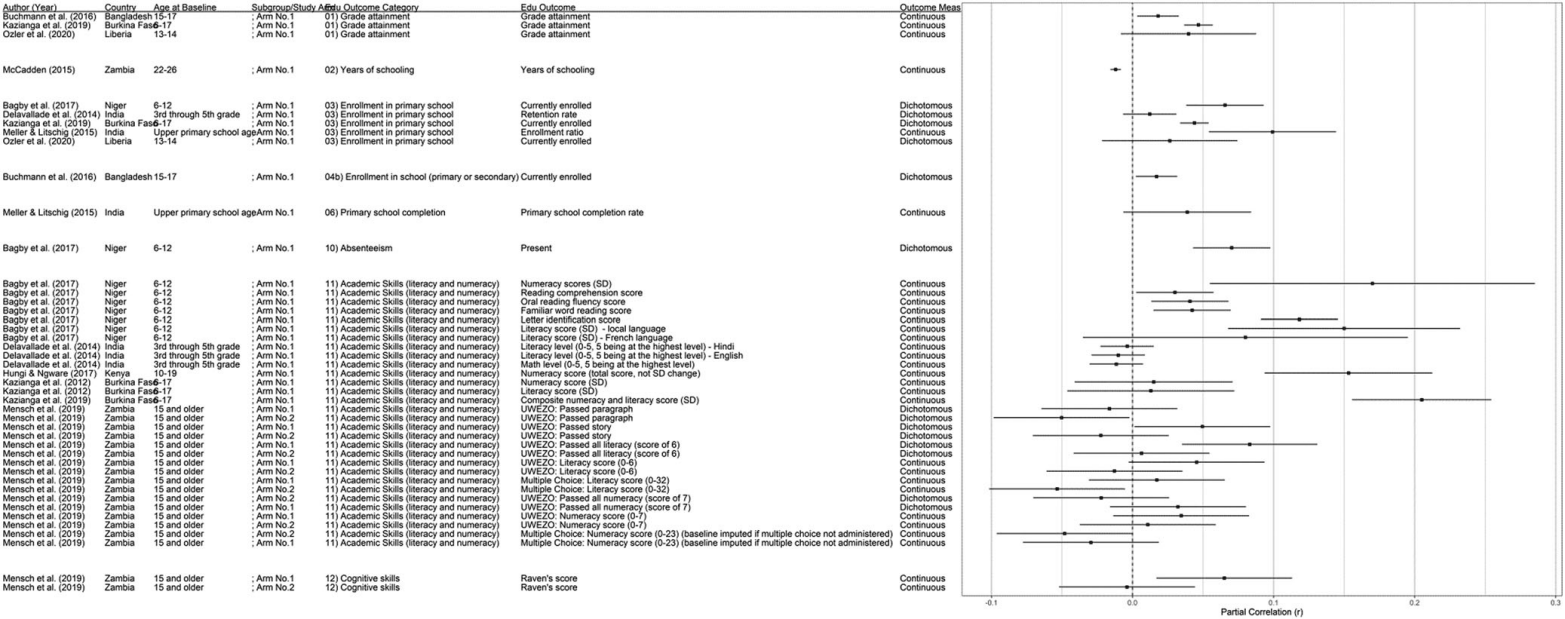
Forest plots showing partial correlation coefficients and 90% confident intervals for direct effects for girls, Barrier 1

**Figure 4.1.2:**
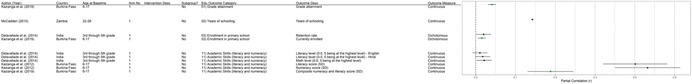
Forest plots showing and 90% confident intervals for direct effects for overall effects for girls and boys combined, Barrier 1* *Green markers indicate that interventions were significantly more effective for girls than boys

We identified nine studies (10 papers)—five experiments and four quasi‐experiments—that investigate the effect of interventions that address the lack of support for girls' schooling on education outcomes (see Table 5.1). Eight of the studied interventions are multi‐component (exception is McCadden, 2015). All nine studies (10 papers) provided estimates of effects on girls (GRADE Summary 1, Figure 4.1.1), and three studies (four papers) estimated the overall effect for girls and boys, and interactions by sex (GRADE Summary 2, Figure 4.1.2). Risk of bias was low for all five of the experimental studies but for quasi‐experimental studies it ranged from low (McCadden, 2015) to some concerns (Kazianga et al., 2012; Kazianga et al., 2019; Meller & Litschig, 2015) to high risk of bias (Hungi & Ngware, 2017). Concerns were largely related to how the authors handled missing data, and whether they were able to address sources of confounding effectively (see Tables 3 and 4).

One of the challenges in understanding the impacts of interventions designed to address this barrier is that they are rarely implemented in isolation. Studies that are grouped under this barrier include interventions or exposures that aimed in part to mobilize community support for girls' schooling and/or motivate parents to keep girls in school. As shown in Table 5.1 the interventions also included a range of other activities, such as safe spaces groups (Mensch et al., 2019; Özler et al., 2020), academic support (Delavallade et al., 2014; Hungi & Ngware, 2017; Meller & Litschig, 2015; Mensch et al., 2019), financial incentives in the form of cash transfers, school materials, or food (Bagby et al., 2017; Buchmann et al., 2016; Hungi & Ngware, 2017; Kazianga et al., 2012; Kazianga et al., 2019; Özler et al., 2020) and school re‐entry policies for adolescent mothers (McCadden, 2015). In many cases it is difficult to tease out the effects of the community or parent engagement activities from the effects of other components of these interventions (Buchmann et al., 2016; Kazianga et al., 2012; Kazianga et al., 2019; McCadden, 2015; Meller & Litschig, 2015). However, two studies provide somewhat clearer evidence (Mensch et al., 2019; Özler et al., 2020), and two provide the most direct evidence (Delavallade et al., 2014; Hungi & Ngware, 2017) of the effects of intervention components focused on increasing support for girls' education on education outcomes.

Özler and colleagues (2020) report on the results of the Girl Empower program in Liberia, which aimed to equip adolescent girls (ages 13–14 at baseline) with the skills and experiences necessary to make healthy, strategic life choices and stay safe from sexual abuse. The core Girl Empower program included: (1) a life skills curriculum, facilitated by local female mentors; (2) caregiver discussion groups; (3) individual savings start‐up for the girls; and (4) capacity building for local health and psychosocial service providers. The caregiver discussion groups, which were one of the more intensive interventions to address this barrier identified in our review, comprised eight monthly sessions focused on familiarizing caregivers with the curriculum content, supporting them in reinforcing the skills girls learned, and encouraging them to support and protect girls in their community. Yet, despite the inclusion of ongoing caregiver/parent engagement sessions, Girl Empower had no significant effect on schooling outcomes.

Similarly, Mensch et al. (2019) report on the results of an RCT evaluating the effects of an after‐school e‐reader literacy program for 7th grade girls in Zambia embedded within a safe space empowerment program using female mentors. Schools were randomized to one of three arms: (1) safe space groups plus community engagement activities; (2) safe space, community engagement, and distribution/use of e‐readers in a facilitated book group; and (3) a control arm. Although both intervention arms included community engagement activities, the e‐reader arm had significant effects on literacy while the safe space groups arm did not. Therefore, it seems unlikely that the community engagement component of that intervention drove improvements in skills on its own. However, it is possible that the effects of skills training operated, in part, through increasing support for girls' education—if parents began to see that their daughters' literacy skills were improving, their attitudes toward the value of girls' education may have shifted.

Work by Hungi and Ngware (2017) provides even more direct evidence of the effects of interventions designed to increase support for girls' education. They report on an evaluation of an incentivized subsidy for 12‐ to 19‐year‐old girls from low‐income households living in Nairobi slums to enroll in secondary school where two treatment packages were implemented: the “T1” group received a subsidy, after school homework support/life skills mentoring and parental counselling, while the “T2” group received only the homework support and life skills components. Parental counselling was carried out by trained mentors and focused on sensitizing the parents about the importance of girls' education. Both interventions led to significant improvement in mathematics achievement for girls, and there was no significant difference between the interventions, indicating that parental counselling on the importance of girls' education had no significant direct benefit. However, as was the case with Mensch et al. (2019), it is also possible that the other components of the program—the subsidy, homework support and life skills mentoring—operated in part through increased support for girls' education at the household or community levels.

Delavallade et al. (2014) also provides more direct evidence of the effects of efforts to increase support for girls' education, based on results of the Educate Girls program in rural Rajasthan, India, which aimed to increase girls' retention, enrolment, and learning. Enrolment and community sensitization activities were specifically aimed at promoting girls' education. Before each school year, a program volunteer engaged in a house‐to‐house enrolment drive, targeting girls in the village who had never been enrolled or who had dropped out, to encourage their parents and the girl to go to school. School Management Committees (SMCs) at each school were also supported to build village capacity and increase local participation in schooling decisions, as well as to sensitize communities to issues related to girls' education and formulate annual School Improvement Plans. The authors report that the learning‐focused activities had no gender component. They found moderate gains in retention and enrolment after 1 year, primarily among disadvantaged girls, and large gains in learning in Hindi, English and math after the 2nd year, with no significant difference by sex. That is, the program was able to increase enrolment, reduce dropout, and increase academic skills for girls, but it was unable to close gender gaps in performance. Despite these mixed results, the findings from this study provide support for the effects of efforts designed to improve support for girls' education among community members, including parents.

Looking across different types of education outcomes, we find very low certainty in the evidence for the effects of interventions aiming to increase support for girls' education, in large part because this approach was not measured in isolation (see GRADE Summary 1). Significant effect sizes were mostly small or medium in size. When limiting our analyses to the programs that provided more direct evidence of the effects of these components, in three out of four cases we find that the community engagement component did not appear to be sufficient to improve education outcomes. However, it is possible that the operation of other components of these programs, such as after school homework support, financial incentives, or school construction, may have been aided in part through increased support for girls' education.

#### 5.3.2 Child marriage and adolescent pregnancy (Barrier 2)

The small number of studies identified for this barrier, as well as lack of direct evidence, lead us to conclude that there is very low certainty in the evidence and more research is needed. We identified only four studies, all multi‐component experiments, that included an explicit focus on child marriage and assessed the effect on education outcomes (Table 5.2). Risk of bias was low for all four studies. No identified studies included adolescent pregnancy prevention as an explicit part of the intervention.

The interventions/exposures included information on the legal age at marriage as part of the life skills component of empowerment programs in Uganda and Sierra Leone (Bandiera et al., 2014; Bandiera et al., 2019, respectively), a school‐based life skills curriculum in India that addressed child marriage, among other topics (Edmonds et al., 2016), and a financial incentive to delay marriage in Bangladesh (Buchmann et al., 2016). The interventions' other components included vocational training (Bandiera et al., 2014, 2019); community mobilization and safe space groups that followed a life skills curriculum which included education support (Buchmann et al., 2016); and mentoring (Edmonds et al., 2016). These other components were often also designed with the aim of contributing to delaying marriage, for example, by increasing girls' agency or through mentors who served as role models and nonfamilial social support.

**Figure 4.2.1:**
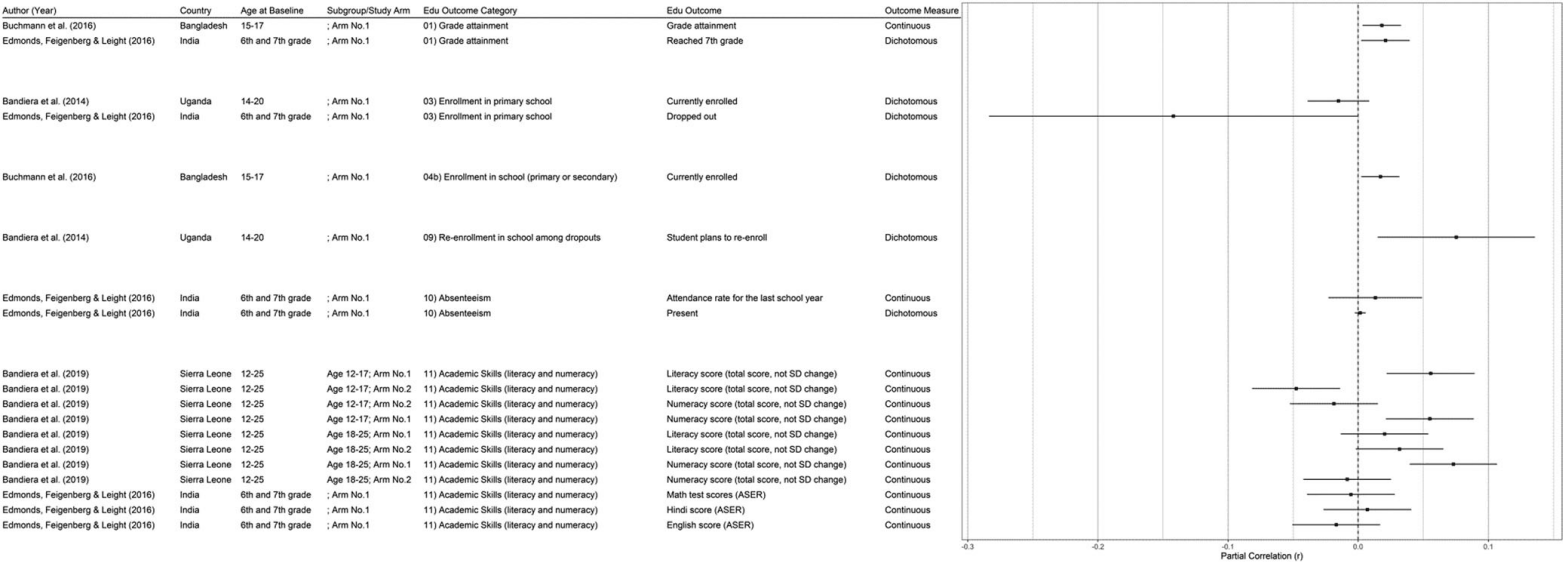
Forest plots showing partial correlation coefficients and 90% confident intervals for direct effects for girls, Barrier 2

Program effects on girls' education outcomes are shown in GRADE Summary 1; Figure 4.2.1. For educational attainment and enrolment outcomes, effects were found across all three studies that measured attainment and/or enrolment (Bandiera et al., 2014; Buchmann et al., 2016; Edmonds et al., 2016). Five out of 6 effect sizes were significant in the expected direction, but effect sizes were small, had wide CIs, or had CIs bordering zero. Only three of 11 academic skills effects were in the expected direction and significant (medium effect size). Academic skills were only reported as outcomes in the Sierra Leone and India evaluations (Bandiera et al., 2019; Edmonds et al., 2016, respectively). Despite the fact that the school‐based program in India included educational support, none of the academic skills effects were significant (Edmonds et al., 2016). All significant academic skills effects were found in the villages that were highly disrupted by Ebola within the empowerment program (safe spaces, life skills, and vocational training) in Sierra Leone (Bandiera et al., 2019). Absenteeism was not affected (only Edmonds examined this indicator).

Further, whether these interventions' impact on attainment/enrolment operated through reductions in child marriage is unclear. We note that only the empowerment program (life skills plus vocational training) in Uganda (Bandiera et al., 2014) and the study arm with the financial incentive to delay marriage in Bangladesh (Buchmann et al., 2016), reduced marriage rates. Between them, they accounted for three of the four significant effect sizes for enrolment and attainment. However, since it was not the aim of their studies, the authors did not attempt to parse out what portion of the effect was direct (intervention improved education outcomes), compared to indirect via reduced child marriage (intervention reduces child marriage and thereby improves education outcomes). Furthermore, the studies/arms that measured but did not have an impact on child marriage—that is, the school‐based life skills program (Edmonds et al., 2016), and the empowerment‐only arm (Buchmann et al., 2016), did not reduce child marriage, but still improved enrolment and attainment outcomes.

Notably, the Bangladesh study, which included three study arms—empowerment program; financial incentive to delay marriage; and empowerment and financial incentive combined—found the arm that combined the financial incentive and life skills did not demonstrate any additional or separate effects on marriage or on schooling (Buchmann et al., 2016).^9^ Overall we find very low certainty of evidence due to the small number of studies, and the lack of direct evidence. More research is needed to determine if efforts to delay child marriage—whether through information, incentives, or empowerment programs, alone or in combination, or through some other means—are a promising path to improvement of education outcomes.

#### 5.3.3 Lack of information on returns to education/alternative roles for women (Barrier 3)

Overall, the mixed results within studies, as well as the small number of studies that measure interventions focused on this barrier in particular, lead us to conclude that while results from some settings are encouraging, certainty of evidence is low or very low and more research is needed. We also find that addressing this barrier tended to improve girls' education outcomes more so than boys' outcomes, but the certainty of evidence was low due to the small number of studies and limited direct evidence.

**Figure 4.3.1:**
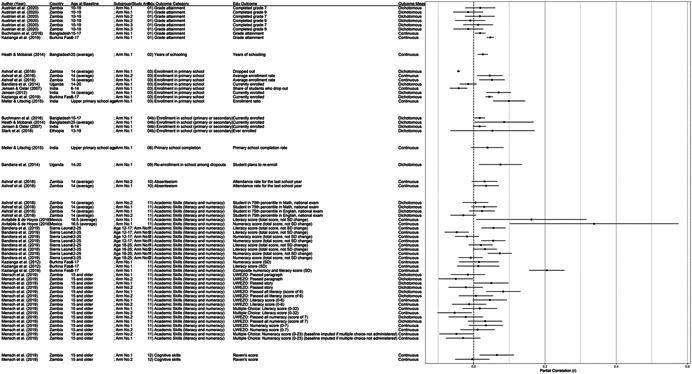
Forest plots showing partial correlation coefficients and 90% confident intervals for direct effects for girls, Barrier 3

**Figure 4.3.2:**
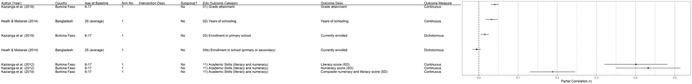
Forest plots showing and 90% confident intervals for direct effects for overall effects for girls and boys combined, Barrier 3* *Green markers indicate that interventions were significantly more effective for girls than boys

We identified 13 studies (14 papers), nine experimental and four quasi‐experimental, that included content on alternative roles for women, provided job recruitment services to young women or provided information about returns to education in the form of labor market opportunities or earnings, and assessed the effect on education outcomes (Table 5.3). Study quality was high with risk of bias low for the majority of studies and four had some concerns (Austrian et al., 2020; Jensen & Oster, 2007; Kazianga et al., 2012; Kazianga et al., 2019; Meller & Litschig, 2015). All 13 of these studies provided estimates of effects on girls (GRADE Summary 1, Figure 4.3.1), and two studies (three papers) estimated the overall effect for girls and boys combined, and interactions by sex (GRADE Summary 2, Figure 4.3.2).

Of the two studies that examined combined effects for girls and boys (GRADE Summary 2, Figure 4.3.2), about half of the effects showed significantly greater improvement for girls than for boys although effect sizes were mostly small. Only one of these two studies focused solely on this barrier, finding a small significant effect on years of schooling for girls and boys combined, with a greater effect for girls, but no significant effect on enrolment (Heath & Mobarak, 2014).

In studies that examined outcomes for girls specifically, all but three evaluations (Heath & Mobarak, 2014; Jensen & Oster, 2007; Jensen, 2012) assessed multi‐component programs. The other activities in the multicomponent programs included information on a higher education scholarship program (Avitabile & de Hoyos, 2018), safe spaces (Ashraf et al., 2018; Austrian et al., 2020; Bandiera et al., 2014; Bandiera et al., 2019; Buchmann et al., 2016; Mensch et al., 2019; Stark et al., 2018), life‐skills or empowerment curricula (Austrian et al., 2020; Bandiera et al., 2014, 2019; Buchmann et al., 2016; Mensch et al., 2019; Stark et al., 2018), negotiation skills (Ashraf et al., 2018), girl‐friendly schools (Kazianga et al., 2012; Kazianga et al., 2019; Meller & Litschig, 2015), school feeding programs (Kazianga et al., 2012; Kazianga et al., 2019), literacy skills (Buchmann et al., 2016; Kazianga et al., 2012; Kazianga et al., 2019; Mensch et al., 2019), supplies and/or books (Kazianga et al., 2012; Kazianga et al.,  2019; Mensch et al., 2019), health vouchers (Austrian et al., 2020); savings accounts (Austrian et al., 2020), and incentives (Buchmann et al., 2016).

The studies that, by design, looked explicitly at the effect of treatments or intervention components more narrowly focused on addressing lack of information on returns to education/alternative role models for women, included two natural experiments (Heath & Mobarak, 2014; Jensen & Oster, 2007) and three RCTs (Ashraf et al., 2018; Avitabile & de Hoyos, 2018; Jensen, 2012). These include the presence of economic opportunities (Heath & Mobarak, 2014; Jensen, 2012), information provided to participants about returns to education (Ashraf et al., 2018; Avitabile & de Hoyos, 2018), and expansion of access to cable television (Jensen & Oster, 2007).

Heath and Mobarak (2014) document the effects of the growth of the garment industry in Bangladesh using retrospective data from 1,395 households in 60 Bangladeshi villages that varied in terms of exposure to garment factories—both distance to garment factories and when the first factories opened. They found that for households that became exposed to garment factories—which improved the returns to education—younger girls were significantly more likely to stay enrolled in school, and older girls were more likely to work for pay, compared to girls in villages that were not commuting distance from factories. These changes led to decreased child marriage and early childbearing, and, the authors argue, contributed substantially to Bangladesh achieving gender parity in school enrolment.

Similarly, Jensen (2012) explored the effect of labor market opportunities in India through an RCT that increased awareness of jobs in India's rapidly growing business process outsourcing (BPO) industry. The intervention used experienced BPO recruiters, assigned to randomly selected rural villages, to increase awareness of these jobs and how to access them. In treatment villages, compared to control villages, young women ages 18–24 were more likely to be enrolled in computer or English‐language courses, girls ages 6–17 were more likely to be enrolled in school, and marriage and childbearing were delayed. As economic opportunities became accessible, returns to education became more salient and human capital investment, including school enrolment, increased.

In a different approach to this barrier, Jensen and Oster (2007) document the impact of the introduction of cable television on gender attitudes in rural India. They use a 3‐year (2001–2003) panel data set covering women in five Indian states to compare changes across villages based on whether and when cable television was introduced. After cable is introduced, they find significantly more gender egalitarian attitudes, and significant reductions in dropout for girls, but not for boys. The authors hypothesize and find some evidence that the mechanism behind these changes is increased exposure to life outside of rural villages but conclude that more research is needed to determine whether this is the pathway of change.

Ashraf et al. (2018) implement an RCT in Lusaka, Zambia among 8th grade girls that tests the effects of a negotiation skills curriculum on enrolment, absenteeism, and academic skills. They find a significant effect of the negotiation curriculum on girls' enrolment—reduced dropout and average enrolment—but not absenteeism or academic skills (English or math). To understand whether it is the negotiation skills themselves, the mentored safe spaces in which the curriculum is provided, or changed perceptions regarding returns to education, the authors “unbundle” the treatment effect with one study arm providing safe spaces only, and cross‐randomize the negotiation treatment with a short information intervention regarding the returns to education. It is the latter that is of interest in this barrier section: they found no effect of the information intervention on enrolment, absenteeism, or academic skills (see Barrier 6 for discussion of the safe spaces only arm).

An informational intervention was also tested in Mexico among 10th grade students (Avitabile & de Hoyos, 2018). In a randomized trial, a short (approximately 12 minute) information package was provided via an interactive computer program. Students in the treatment arm received gender‐specific information on average earnings for different levels of educational attainment, information on life‐expectancy, and information on a higher education scholarship program. The intervention had no effect on on‐time high school completion, but significantly improved literacy and math scores for girls (boys improved in math only). However, the effect is not significant for students from low‐income households, thus exacerbating inequalities.

In sum, looking at the five studies that provided more direct evidence regarding interventions that aim to shift perceptions of returns to education or provide alternative role models for girls and women, we see encouraging results. However, given our low confidence in the results due to the disparate interventions and inconsistent effects, we conclude that more research is needed. Interventions that more directly addressed this barrier varied substantially, with two studies assessing the effects of economic opportunities, two that included treatment arms evaluating the provision of information to participants about returns to education, and one measuring the effects of expansion of cable television access. In some settings and time points these interventions were somewhat encouraging for improving enrolment for girls (Heath & Mobarak, 2014—medium effect with wide CI bordering zero; Jensen, 2012—medium effect; Jensen & Oster, 2007—small effect and no effect), but not in others (Ashraf et al., 2018). The two interventions that measured academic skills also had inconsistent results—Ashraf and colleagues' information arm had no effect on girls' academic skills, whereas Avitabile and de Hoyos' short information intervention did (2/2 effects: 1 medium, 1 small with CI bordering zero), although there were no significant effects among low‐income students.

#### 5.3.4 School‐related gender‐based violence (SRGBV) (Barrier 4)

We did not identify any evaluations of SRGBV programs that assessed education outcomes. Research is needed to determine whether such interventions improve education outcomes, and, if so, through what pathways for different outcomes—enrolment/attainment and academic skills.

#### 5.3.5 Gender insensitive school environment (Barrier 5)

While results of interventions fostering gender sensitive school environments are encouraging for their effects on academic skills, the small number of studies providing direct estimates of effects on girls' enrolment and attainment outcomes leads us to conclude that more research is needed.

**Figure 4.5.1:**
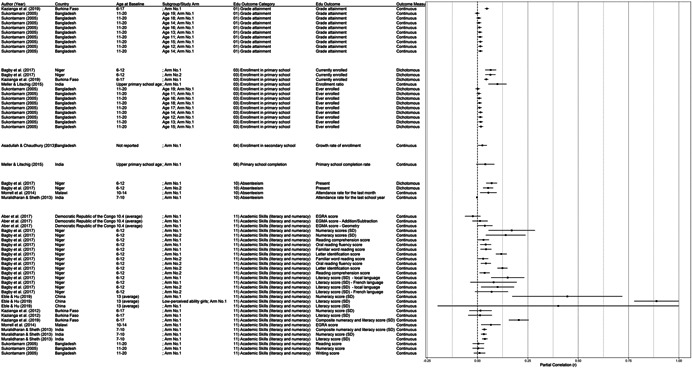
Forest plots showing partial correlation coefficients and 90% confident intervals for direct effects for girls, Barrier 5

**Figure 4.5.2:**
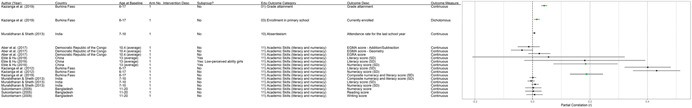
Forest plots showing and 90% confident intervals for direct effects for overall effects for girls and boys combined, Barrier 5* *Green markers indicate that interventions were significantly more effective for girls than boys

We identified nine studies (10 papers), seven multicomponent, three experimental, that addressed a gender insensitive school environment defined very broadly (Table 5.5). Interventions under this barrier fell into roughly three categories: those that provided “girl‐friendly schools” and often a number of other amenities or program components (Bagby et al., 2017; Kazianga et al., 2012; Kazianga et al., 2019; Meller & Litschig, 2015); those that provided teacher training in participatory, learner‐centered pedagogies, and in one instance also included teacher support groups (Aber et al., 2017; Morrell et al., 2014); and those that assessed the effects of having female teachers (Asadullah & Chaudhury, 2013; Eble & Hu, 2019; Muralidharan & Sheth, 2013; Sukontamarn, 2005). Study quality was mixed: four studies had low risk of bias (Bagby et al., 2017; Eble & Hu, 2019; Muralidharan & Sheth, 2013; Sukontamarn, 2005), four had some concerns (Aber et al., 2017; Asadullah & Chaudhury, 2013; Kazianga et al., 2012; Kazianga et al., 2019; Meller & Litschig, 2015) and one had high risk of bias due primarily to the risk of confounding (Morrell et al., 2014). Nine studies (10 papers) provided estimates of effects on girls (GRADE Summary 1, Figure 4.5.1), and five studies (six papers) estimated the overall effect for girls and boys combined, and interactions by sex (GRADE Summary 2, Figure 4.5.2).

Evidence was largely indirect—either programs also included other substantial components, especially access interventions such as school construction, that may explain beneficial outcomes—or they were focused narrowly on provision of female teachers or on training in learner‐centered pedagogies, which are both elements of girl‐friendly schools, but, arguably, not sufficient by themselves to make a school girl‐friendly.

Among the nine studies that estimated effects for girls (Aber et al., 2017; Asadullah & Chaudhury, 2013; Bagby et al., 2017; Eble & Hu, 2019; Kazianga et al., 2019; Meller & Litschig, 2015; Morrell et al., 2014; Muralidharan & Sheth, 2013; Sukontamarn, 2005) evidence is largely indirect, as the types of programs identified under this barrier range from single component interventions that assess the impact of providing female teachers and do not fully embody girl‐friendly schools, to multicomponent programs that seek to create girl‐friendly schools but also include components such as school construction that are not separated out in the study design. Nonetheless, studies on both ends of this spectrum offer insight. Three studies assessed the effects on learning outcomes of having female, versus male, teachers. Two studies did so using representative panel data on education from China and the Indian state of Andhra Pradesh, respectively (Eble & Hu, 2019; Muralidharan & Sheth, 2013). While the study in China looked at middle school, and the study in India examined primary school, both find that girls perform significantly better with female teachers than male teachers, particularly in regard to math scores.^10^ There are similar trends in both studies for language scores, but they are less marked than for math. Muralidharan and Sheth (2013) suggest that having a teacher of the same sex matters more when there are negative stereotypes such as girls being worse at math than boys. In their analysis, Eble and Hu (2019) make the same point—that societal beliefs about ability by gender contribute to the power of having a teacher of the same sex, and that this interacts with a child's beliefs about their own ability. In a third study evaluating the Bangladesh Rural Advancement Committee's (BRAC's) nonformal primary schools, Sukontamarn (2005) found that a high percentage of female teachers was one of the factors that explained girls' higher enrolment.

Three studies assessed (very) broad efforts to provide girl‐friendly schools in Niger (Bagby et al., 2017), India (Meller & Litschig, 2015), and Burkina Faso (Kazianga et al., 2012; Kazianga et al., 2019). All focused on primary school, included construction of classrooms or schools and an array of complementary activities. In Niger these included housing for female teachers, a preschool, separate latrines for girls and boys, new boreholes, community mobilization in support of girls' education, provision of textbooks and school materials, local language reading materials, promotion of gender‐equitable classrooms, mentoring, SMCs, deworming, among others (Bagby et al., 2017). In India, additions were optional and included day care centers for younger siblings, flexible timing of classes, gender sensitization for teachers, remedial classes, bridge courses to re‐enroll drop‐outs, vocational training, and girls' toilets (Meller & Litschig, 2015). Complementary components in Burkina Faso included daily meals for all, take home rations, textbooks, school supplies, mobilization campaigns to address barriers to girls' education, adult literacy, mentoring, and training of local officials, teachers, and so forth (Kazianga et al., 2012; Kazianga et al., 2019). Many of these elements are approaches described under other barriers in this review (see, e.g., Lack of water and sanitation (Barrier 13) or Inadequate school access (Barrier 14)). While none of the evaluations was designed to unpack the effects of specific components, Bagby and colleagues note that because there was no difference in the availability of schools across villages at the end of the evaluation, the effects of the program in Niger are a result of improved quality of education and educational environment rather than school construction.

The girl‐friendly school packages of interventions led to significant improvements in all three settings, closing the gender gap in program settings in Burkina and India. In the study in India, because the gender gap in enrolment in comparison communities had closed, the program led to a gender gap in favor of girls. In Niger, the evaluation found a 10.3 percentage point increase in primary school enrolment, 13.6 percentage point increase in attendance, and a 0.21 standard deviation effect on local language test scores, though no effect on French language test scores (Bagby et al., 2017). In India, the program led to an increase in enrolment for girls of 6–7 percentage points, and nonsignificant increases in school completion (Meller & Litschig, 2015). Program effects were largest in Burkina Faso, with an increase in enrolment of 15.5 percentage points, and improvements of 0.29 standard deviations on standardized achievement tests (Kazianga et al., 2012; Kazianga et al., 2019).

Notably, in two evaluations of girl‐friendly schools that included girls and boys and disaggregated results by sex, authors found that while girls benefited significantly, boys also experienced gains (Bagby et al., 2017; Meller & Litschig, 2015). However, both these programs included multiple components including school construction that could explain these effects. Five studies (six papers) assessed the combined effects for girls and boys (Aber et al., 2017; Eble & Hu, 2019; Kazianga et al., 2012; Kazianga et al., 2019; Muralidharan & Sheth, 2013; Sukontamarn, 2005). About one‐third (7/18) of overall effect estimates were significant in the expected direction (3 small, 2 medium, 2 large). Less than one quarter (4/18; 2 small and 2 medium) showed significantly greater improvement for girls than for boys.

In sum, breaking findings out by outcome type, we find that there are encouraging patterns, but low confidence in the evidence, with many of the effect sizes small and indirectness a concern. For studies measuring improvement of academic skills, we have moderate confidence in the evidence, though we caution that the complexity of assessing this barrier makes interpretation more difficult. For the five single‐component interventions, results were inconsistent and most significant effects were small. In the two multi‐component programs effects ranged from null to medium size effects, with the majority significant. While the evidence from these studies suggests that interventions to improve the gender sensitivity of school environments are encouraging, and led to beneficial effects in some settings, more research is needed. Furthermore, because the studies varied widely in the number and type of programmatic elements investigated, and many did not tease out the direct effects of individual components, we are unable to draw specific conclusions about which component(s) are most likely to promote a gender sensitive school environment, and, if so, whether that is what leads to improved education outcomes.

#### 5.3.6 Lack of safe spaces and social connections (Barrier 6)

We find mixed results for programs that address a lack of safe spaces and social connections, resulting in very low to low confidence in the evidence. We conclude that more research is needed. However, existing evidence indicates that safe/protected spaces alone may be insufficient to lead to improvements in education outcomes, especially skills, without provision of additional training or economic empowerment components.

**Figure 4.6.1:**
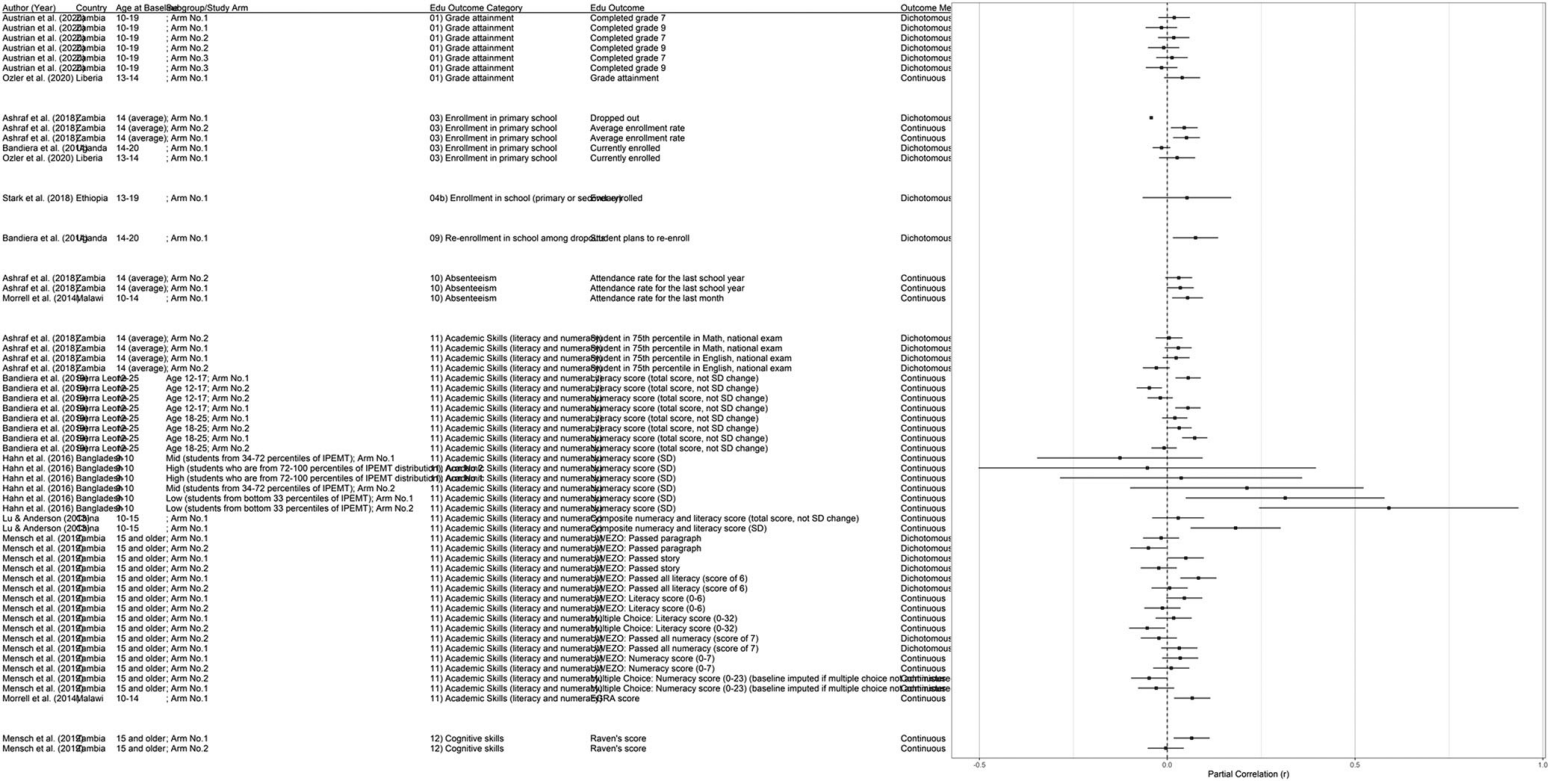
Forest plots showing partial correlation coefficients and 90% confident intervals for direct effects for girls, Barrier 6

We identified 10 papers (each representing a different study), nine experimental and one quasi‐experimental, that addressed the lack of safe spaces and social connections (see Table 5.6). Eight of the nine experimental studies had a low risk of bias; the remaining study (Austrian et al., 2020) had some concerns due to high attrition and unbalanced arms at baseline following randomization. The one quasi‐experimental study (Morrell et al., 2014) had a high risk of bias due to a lack of reported information about the methods (see Tables 3 and 4). All 10 of the studies reported effects of the interventions for girls, rather than pooled effects for boys and girls (see GRADE Summary 1, Figure 4.6.1).

Table 5.6 shows the included studies by the barriers they address. Seven of the interventions were conducted via a platform described as a “safe” or “protected” space; all seven were multicomponent and conducted meetings that were facilitated by female mentors. In most cases, the safe/protected space was combined with the delivery of a life skills curriculum by a female mentor (exceptions were Hahn et al., 2016; Lu & Anderson, 2015) as well as providing information on returns to education or alternative roles for girls (exceptions were Hahn et al., 2016; Lu & Anderson, 2015; Morrell et al., 2014; Özler et al. 2020). Therefore, it is difficult to disentangle the effects of the curriculum and mentor (as well as other study components) from the effects of the safe spaces themselves. However, Ashraf et al. (2018) report on the results of a four‐arm study: (1) a negotiation group in which female coaches led six after school sessions over the course of two weeks for groups of about 15–20 girls training them in negotiation and interpersonal communication; (2) a safe space “placebo” group in which girls participated in the same type of groups, led by the same female coaches, but instead of the negotiation curriculum girls could play games, talk, or do homework; or (3) a control group. Unlike the other evaluations in this group, the inclusion of a “placebo” arm provides an estimate of the effects of safe/protected spaces (including mentors) on their own. The authors find small but statistically significant effects of the safe spaces group on enrolment, though they observe no effects on absenteeism or academic skills. The arm that included negotiation training had significantly stronger effects for most outcomes.^11^ Mensch et al. (2019) also did not report significant effects of the safe space intervention arm on education outcomes. Rather, significant effects on literacy were observed in the arm that included those components, in addition to facilitated book groups and provision of e‐readers.

Although most of the included studies were unable to isolate the effects of safe space interventions on their own, results were mixed, and did not reveal a strong pattern of effective interventions, even when other components were present.

The authors of included studies with null results provide a number of explanations for why traditional safe space programs on their own may not be effective in improving education outcomes. In the case of Austrian et al. (2020), where the intervention targeted particularly vulnerable girls in Zambia who face considerable social and economic barriers, participation was low; only 30% attended half or more of the safe space sessions, and 25% did not attend at all. The authors note that if the economic constraints faced by girls and their households are not addressed, educational outcomes are unlikely to improve, an observation also made by Stark et al. (2018) in explaining the null findings for the safe spaces intervention they evaluated in Ethiopian refugee camps. In the case of the Girl Empower intervention evaluated by Özler et al. (2020) in Liberia, the authors note the lack of a “systematic strategy to engage with community” in settings such as Liberia with entrenched gender norms and “habituation to conflict,” implying that establishing safe spaces alone may not be enough to either address or circumvent community‐level expectations about girls.^12^

Three of the included studies took a more narrowly defined approach to girls' groups, investigating the effects of: (1) female study groups in Bangladesh (Hahn et al., 2016), (2) seat assignment near other girls in China (Lu & Anderson, 2015) and (3) “girl‐friendly” extracurricular activities led by female teachers in Malawi (Morrell et al., 2014). Results from these interventions, with a clearer focus on building girls' academic skills, were more promising overall, though many of the effect sizes were small.

While providing a space for girls to meet with female mentors may reduce the isolation they face in many settings and build their social assets, absent other effective components (e.g., literacy and/or numeracy training, negotiation training, or financial incentives), we find little evidence that mentored groups alone will improve education outcomes, especially academic skills. More research is needed to disentangle the different components and pathways of change for safe space‐based interventions with girls.

#### 5.3.7 Lack of teaching materials and supplies (Barrier 7)

Interventions addressing lack of teaching materials and supplies were all multicomponent and evaluations did not disentangle the distinct effects of providing teaching materials and supplies. Moreover, in some studies, materials and supplies comprised a minor element of a much larger program. We thus have very low confidence in the evidence and additional research is needed. Further, we found no studies that evaluated programs removing gender bias from textbooks or other teaching materials.

**Figure 4.7.1:**
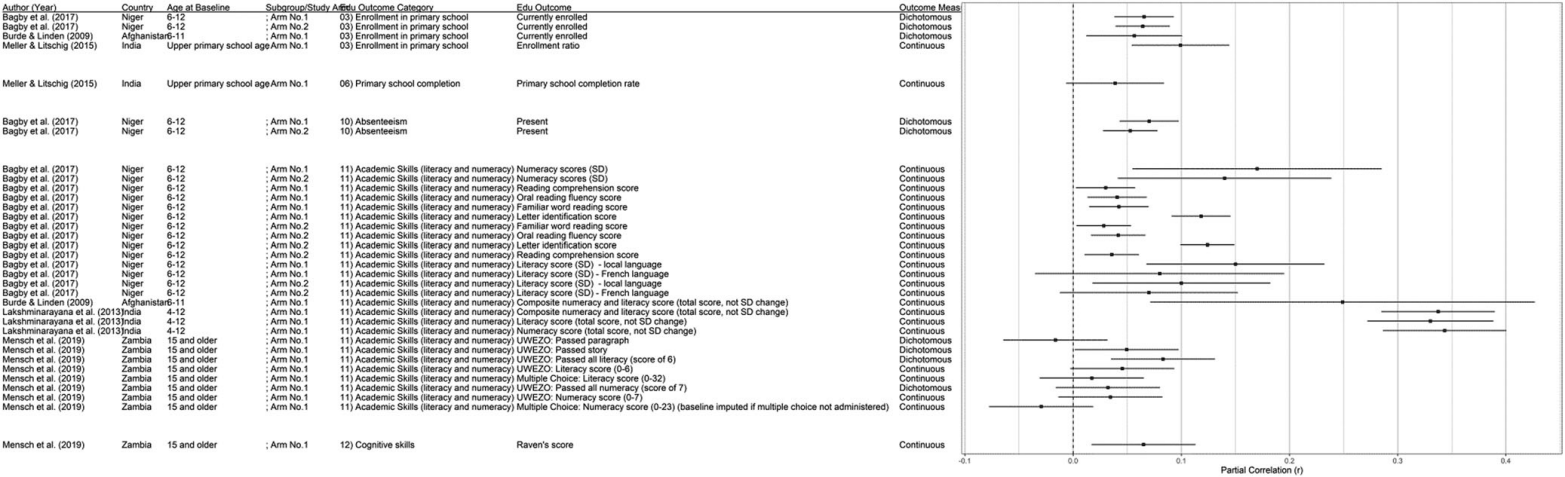
Forest plots showing partial correlation coefficients and 90% confident intervals for direct effects for girls, Barrier 7

**Figure 4.7.2:**
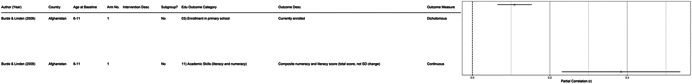
Forest plots showing and 90% confident intervals for direct effects for overall effects for girls and boys combined, Barrier 7* *Green markers indicate that interventions were significantly more effective for girls than boys

We identified five studies, four experimental and one quasi‐experimental, that included providing girls with teaching materials and supplies (Table 5.7). Study quality was high, with most having a low risk of bias and one (Meller & Litschig, 2015) having some concerns. All five studies provided estimates of effects on girls (GRADE Summary 1; Figure 4.7.1) and one study estimated the overall effect for girls and boys combined, and interactions by sex (GRADE Summary 2; Figure 4.7.2).

Within the five studies that estimated effects for girls, the majority of effects for enrolment/attainment were significant (medium size). For academic skills the majority of effects were also significant, ranging in size from small to large. The one paper that examined combined effects for girls and boys (GRADE Summary 2), had a significant impact on both enrolment/attainment and academic skills. Two out of two estimated overall effects were in the expected direction, one medium size, the other large but with a wide CI, and both showed significantly greater improvement for girls than for boys (Burde & Linden, 2009).

All five programs were multicomponent. Two of these included teaching materials as part of efforts to make schools more gender sensitive and these programs included many other components such as construction of classrooms, housing for female teachers, preschools, separate latrines for girls and boys, community mobilization in support of girls' education, promotion of gender‐equitable classrooms, mentoring, SMCs, deworming, day care centers for younger siblings, flexible timing of classes, gender sensitization for teachers, remedial classes, bridge courses to re‐enroll drop‐outs, and vocational training (Bagby et al., 2017; Meller & Litschig, 2015). In this context, the provision of teaching materials and supplies likely played a minor role, and the effect of this component on education outcomes was not isolated by the evaluation design. Another study included teaching materials and supplies as part of an effort to provide community‐based schools in Afghanistan (Burde & Linden, 2009). This intervention, and its evaluation, focused tightly on the supply‐side provision of schools and how reduced distance to schools improves education outcomes, especially for girls. The study design does not allow for examining whether the inclusion of teaching materials contributed to the impact of the program.

The last two programs included teaching materials and supplies in the context of interventions focused more directly on this barrier (Lakshminarayana et al., 2013; Mensch et al., 2019). In both studies, the teaching materials were provided in the context of after school groups led by teachers/facilitators who were trained and provided with curricula and/or guidance on pedagogy. Lakshminarayana et al. (2013) evaluated a program that provided learning materials and supplementary teaching to public primary students in grades two through four in Andhra Pradesh, India. In addition, in half the intervention villages girls also received kits comprised of a school uniform, shoes, socks, undergarments, and a school bag, though the effects of this additional intervention were not compared to the control group. Remedial instruction using cooperative‐reflective learning pedagogy, which aims to promote peer learning, higher order thinking skills, and leadership, was provided in school after normal school hours over the course of two academic years, and reinforced what was being taught in schools. Accompanying learning materials were interactive and designed to strengthen learning of concepts and problem solving. Participants were also provided with learning materials such as pens, notebooks, and erasers. The evaluation found significant improvements in composite test scores, math scores, and language test scores compared to controls for both girls and boys. There was no significant difference between girls' and boys' outcomes after adjusting for baseline differences.^13^

In Zambia, Mensch et al. (2019) evaluated a program for grade 7 girls in public school that provided mentor‐led safe spaces, an empowerment curriculum, and e‐readers with accompanying curriculum to facilitate reading and discussion in the groups. Each e‐reader had about 100 books, mostly fiction, of varying reading levels. Girls kept their e‐reader over the course of the 6‐month intervention, taking them home and keeping them over school breaks. To tease out the effects of the e‐reader and facilitated book group, a third study arm provided just the mentor‐led safe spaces and empowerment curriculum. Both intervention arms also included a modest community engagement component. The evaluation found that girls in the e‐reader arm scored significantly better than girls in the control arm on two of three basic literacy assessments and on a non‐verbal reasoning assessment. While girls in the e‐reader arm also had higher scores for more advanced literacy assessments, these improvements were not significant and no effect on numeracy was observed. The safe‐spaces only arm had no significant effect on any of the academic or cognitive skill outcomes, suggesting that exposure to a facilitated book group and access to an e‐reader with engaging content for adolescent girls can improve basic literacy skills and reasoning ability.

While indirectness concerns give us very low confidence in the results and we cannot conclude that teaching materials and supplies are effective interventions in and of themselves, in the context of multicomponent programs they may play a beneficial role. More research is needed on this question. Furthermore, no studies explicitly tested gender‐equitable textbooks and, indeed, little mention is made of gender‐biased textbooks, although Mensch et al. (2019) noted the selection of books for e‐readers included “strong female protagonists and nontraditional gender roles.”

#### 5.3.8 Insufficient academic support (Barrier 8)

We find evidence that programs that provided training or remedial support, many of which also integrated technology, are effective at improving learning outcomes for girls, with moderate confidence in the results based on existing evidence including small, medium and large effect sizes. We also find some indications that these programs improve learning for girls and boys combined, but they do not appear to narrow gender gaps in learning. We find insufficient direct evidence as to whether these interventions improve school enrolment or attainment for girls.

We identified 13 studies (15 papers), four quasi‐experimental and nine experimental, all multi‐component (see Table 5.8). Eight out of the nine experimental studies had a low risk of bias; the exception (Cho et al., 2019) had some concerns due to a lack of information about the randomization procedure, and some imbalances at baseline. Risk of bias in the quasi‐experimental studies varied, from low (Okurut, 2015; Okurut, 2018) to some concerns (Meller & Litschig, 2015) to high risk of bias due to failure to properly control for confounding or deal with missing data and lack of information about methods (Hungi & Ngware, 2017; Morrell et al., 2014) (see Tables 3 and 4).

**Figure 4.8.1:**
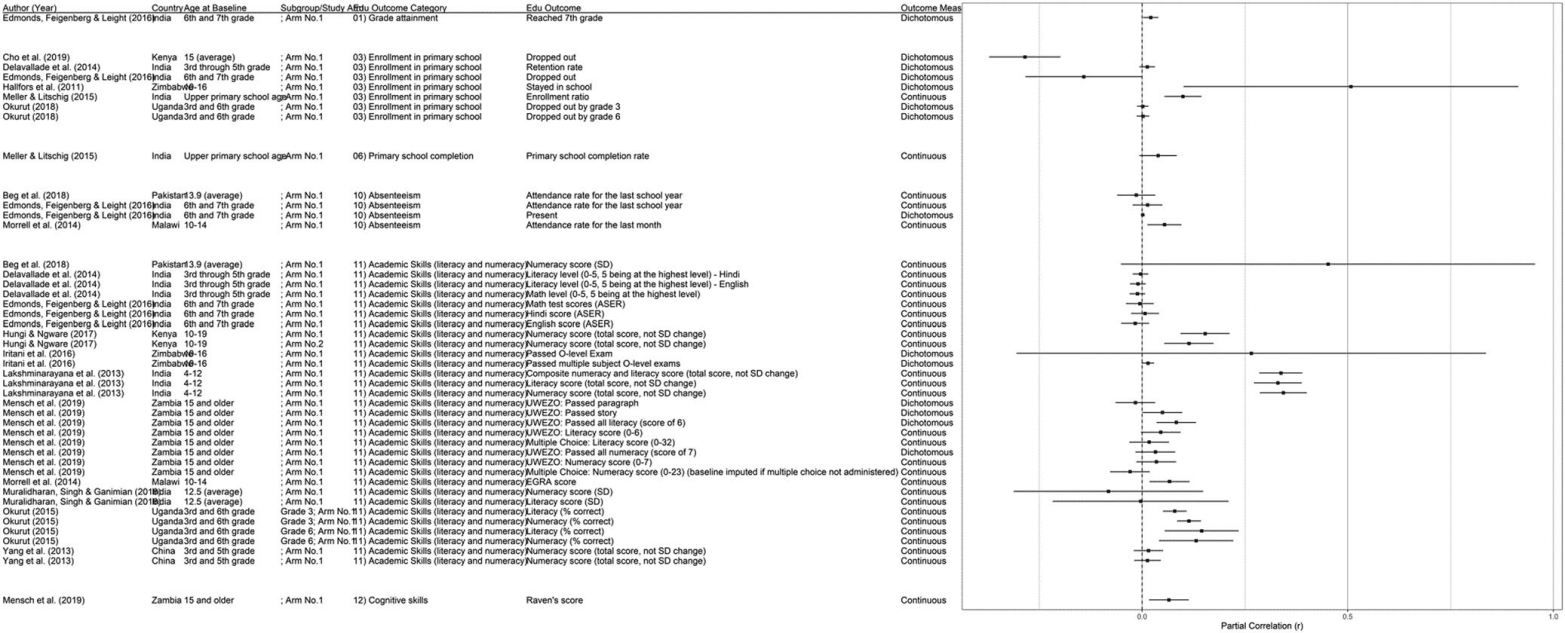
Forest plots showing partial correlation coefficients and 90% confident intervals for direct effects for girls, Barrier 8

**Figure 4.8.2:**
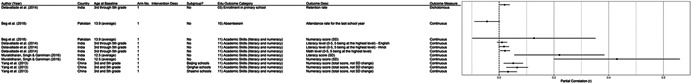
Forest plots showing and 90% confident intervals for direct effects for overall effects for girls and boys combined, Barrier 8

The interventions in these studies included provision of academic support conceptualized broadly in the form of: (1) additional educational content beyond what is usually offered in class; (2) homework assistance; (3) remedial help; and (4) counselling to address attendance issues. All 13 studies estimated effects on education outcomes for girls (GRADE Summary 1 and Figure 4.8.1), while four estimated pooled effects for girls and boys and differences by sex (GRADE Summary 2 and Figure 4.8.2) (Beg et al., 2018; Delavallade et al., 2014; Muralidharan et al., 2016; Yang et al., 2013).

As with the other barriers, two challenges emerge when trying to disentangle the effects of interventions designed to address lack of academic support: (1) they took different forms and were implemented in different settings; and (2) they were often combined with other program components, making it difficult to isolate the direct effects of activities focused on academic support (see Table 5.8). Some of the interventions evaluated in these studies provided a form of academic support as part of a larger multi‐component program, some of which included school fees or other financial incentives (Cho et al., 2019; Hungi & Ngware, 2017; Iritani et al., 2016; Hallfors et al., 2011), while others focused more directly on training or remedial support (Beg et al., 2018; Lakshminarayana et al., 2013; Mensch et al., 2019; Muralidharan et al., 2016; Yang et al., 2013), mentoring and life skills education (Edmonds et al., 2016; Morrell et al., 2014), school policy or infrastructure changes (Meller & Litschig, 2015; Okurut, 2015, 2018) or efforts to increase community support for girls' education (Delavallade et al., 2014). However, the variety of interventions captured in this barrier also illuminates some important findings.

Focusing in on the six studies that directly evaluated interventions with a strong training or remedial academic support focus, we find that the approaches varied. Four out of the six interventions included a technology component, and in each case was connected to a curriculum and/or facilitated by a teacher or mentor. Mensch et al. (2019), which tested an after‐school e‐reader literacy program for 7th grade girls in Zambia, embedded this component within a safe space empowerment program using female mentors. Lakshminarayana and colleagues (2013) report on a government program in India that provided two‐hour after school instruction classes using supplementary teaching and learning materials, while Delavallade et al. (2014) evaluate the Educate Girls program in India, which include after‐school “games‐based” education for girls and boys several times a week over 3 months. Beg and colleagues (2018) discuss the effects of a “light touch” eLearn intervention tested in schools in Pakistan that involved multimedia video presentations corresponding to topics in the curriculum. Muralidharan et al. (2016) report on an evaluation of the after school Mindspark program in India, a technology‐led instructional program developed by an Indian education firm. Finally, Yang and colleagues (2013) studied the effects of computer‐assisted learning (CAL) remedial tutoring sessions designed to complement in‐class math and language curricula in China. All six programs reported some improvements in academic skills for girls.^14^ None of the evaluations that reported results for girls and boys found differences in improvements, meaning that they neither narrowed nor widened gender gaps.

Turning to the studies that looked more indirectly at academic support components, two focused largely on mentoring and life skills education programs, with academic support as a component within the larger interventions. The Girls Education Program (GEP) in India focused on life skills education and mentoring, implemented in schools by a “social mobilizer” (SM), who also acted as a female role model and mentor, including providing girls with support services to stay in school as needed. The evaluation observed declines in school dropout and increases in school progression, but no improvements in attendance or learning (Edmonds et al., 2016). Similarly, the program evaluated by Morrell and colleagues (2014) trained female teachers to run participatory, girl‐friendly, extracurricular activities focused on improving girls' self‐confidence, sexual and reproductive health and academic skills in Malawi. In contrast to the GEP in India, the program in Malawi led to an improvement in attendance and academic skills for girls (Morrell et al., 2014). However, in both cases, it is difficult to disentangle the effects of the life skills education and mentoring from any additional academic support provided through the program (see results for *Inadequate Life Skills (Barrier 11)* for more information).

The remaining studies included under this barrier examined a variety of different programs—some effective, others less so. Like the programs focused on mentoring and life skills education, other programs included numerous components, making it difficult to isolate any independent effects of academic support activities (Hallfors et al., 2011; Iritani et al., 2016; Meller & Litschig, 2015). One of the intervention arms in the program evaluated by Hungi and Ngware (2017) provided an incentivized subsidy for 12‐ to 19‐year‐old girls from low‐income households living in Nairobi slums to enrol in secondary school based on their primary school leaving exam score, along with after school homework support and life skills mentoring. The program led to substantial improvements in academic skills, which may have been in part due to the academic support component. Similarly, Cho et al. (2019) found that a program providing school uniforms and payment of secondary school fees, in addition to academic support to resolve absenteeism issues, led to improvements in primary school enrolment.^15^ A similar model in Zimbabwe, including payment of school fees, uniforms, additional school supplies (including sanitary napkins, underpants and soap) and a school‐based helper to monitor attendance, led to improvements in dropout but not in academic skills (Hallfors et al., 2011; Iritani et al., 2016). After the third round of follow‐up, school fees were offered to the control group. The authors report that the comprehensive intervention (fees plus academic support and additional supplies) was more effective than the fees alone. They also find that the intervention increased bonding with schools and teachers, and participants were more likely to feel that teachers cared about them—a finding that provides an additional mechanism through which academic support might operate to improve dropout (Hallfors et al., 2011; Iritani et al., 2016). However, given previous evidence from other reviews, the effects of these studies may be attributable to the financial incentives included in each of these programs (Baird et al., 2013; Snilstveit et al., 2015). In contrast, the Educate Girls program evaluated in India by Delavallade and colleagues (2014) combined after‐school academic support for all students with community enrolment and support to SMCs focused on girls' education. They found moderate gains in enrolment and attainment, especially for girls, but more substantial gains in learning for all students.

Finally, the papers by Okurut (2015, 2018) found that the AP policy in Uganda, aimed at encouraging children to stay enrolled from primary to secondary school, led to significant improvements in literacy and numeracy for girls and boys in primary school (Okurut, 2015), and more mixed effects on dropout (Okurut, 2018). The policy change, which included the addition of remedial classes before and after school for academically weak students, neither narrowed nor widened gender gaps in learning or dropout (Okurut, 2015, 2018).

In sum, when we focus in on programs with targeted training or remedial support, we find evidence that in some settings these interventions are effective at improving academic skills for girls, demonstrating small, medium, and large effect sizes, with moderate confidence in these results based on existing evidence. However, we find weak/insufficient evidence for effects of these interventions on enrolment or attainment for girls due to indirectness concerns and mixed results.

#### 5.3.9 Inadequate sports programs and health and childcare services for girls (Barriers 9 and 10)

No studies directly evaluating sports or childcare services interventions were identified—more research is needed.

**Figure 4.9.1:**
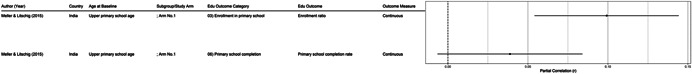
Forest plots showing partial correlation coefficients and 90% confident intervals for direct effects for girls, Barrier 9

**Figure 4.10.1:**
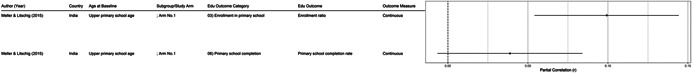
Forest plots showing partial correlation coefficients and 90% confident intervals for direct effects for girls, Barrier 10

Only one study, Meller and Litschig (2015), a quasi‐experimental study in India, included any mention of sports equipment or health and childcare services for girls (Tables 5.9 and 5.10; Figure 4.9.1 and 4.10.1; GRADE Summary 1). We found some concerns with regard to risk of bias in this study due to issues with controlling for confounding adequately, dealing with missing data, and measurement of outcomes. The intervention included new sports equipment as part of its infrastructure upgrade and offered, among its menu of services, the possibility of day care for younger siblings. However, the intervention had so many elements, including construction, flexible timing of classes, gender sensitization for teachers, remedial classes, bridge courses to re‐enroll drop‐outs, vocational training, and girls' toilets, that it is not possible to draw any conclusions about whether provision of sports equipment or health and childcare services makes a difference for school enrolment or primary school completion among girls.

#### 5.3.10 Inadequate life skills (Barrier 11)

While evidence on life skills interventions in some settings suggest they may be effective in improving education outcomes, more stringent analysis leads us to conclude that more research is needed, as mixed results and the small number of studies that disentangle the discrete effects of life skills education give us low confidence in the evidence.

**Figure 4.11.1:**
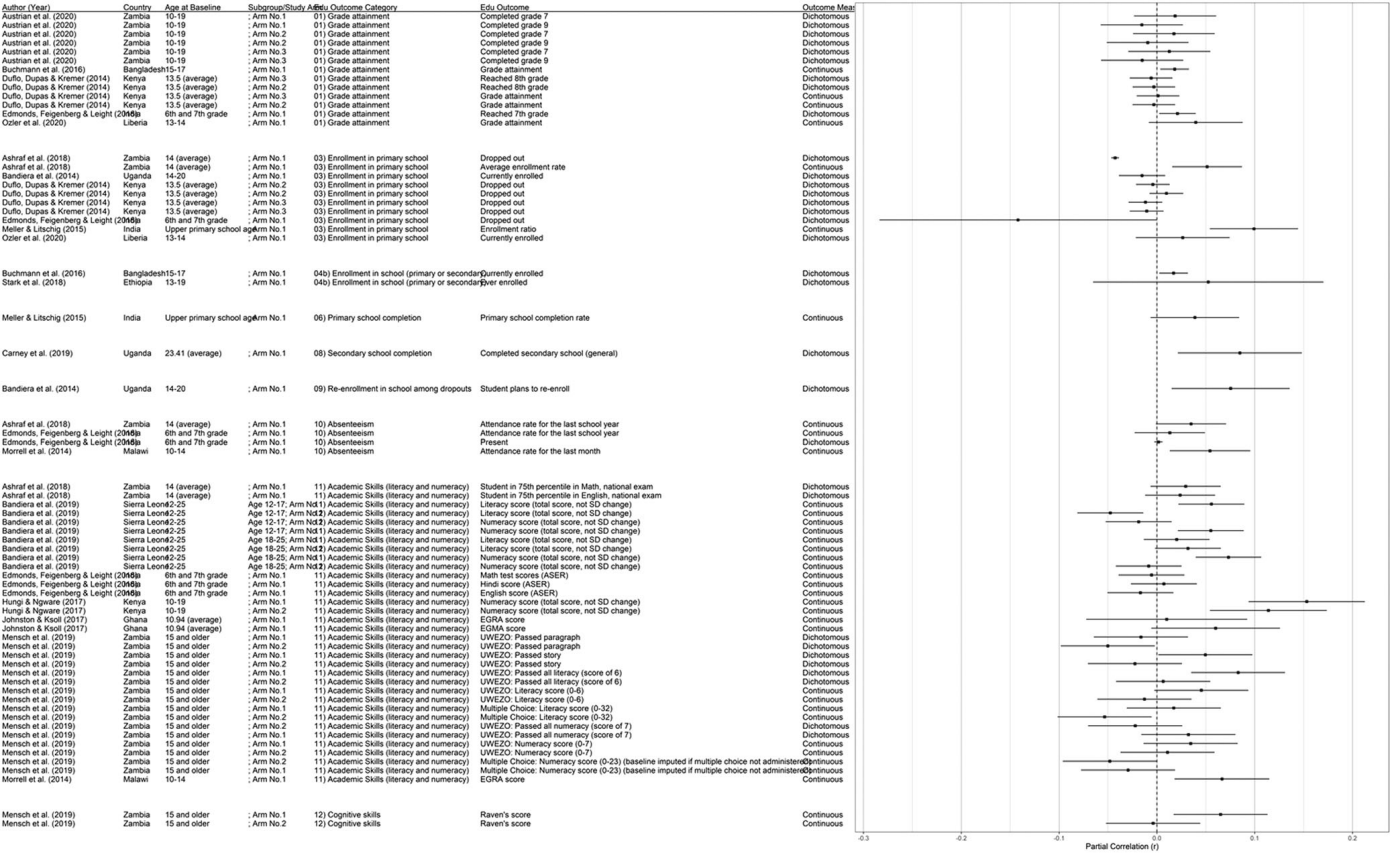
Forest plots showing partial correlation coefficients and 90% confident intervals for direct effects for girls, Barrier 11

**Figure 4.11.2:**
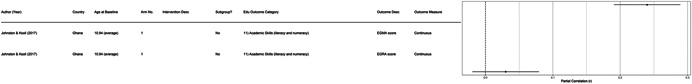
Forest plots showing and 90% confident intervals for direct effects for overall effects for girls and boys combined, Barrier 11

Fifteen studies assessed interventions that aimed to improve life skills (Table 5.11). Twelve used experimental and three used quasi‐experimental designs. Risk of bias was generally low, with the exception of two studies with high risk of bias (Hungi & Ngware, 2017; Morrell et al., 2014) due to factors such as high risk of confounding, and two with some concerns (Austrian et al., 2020; Meller & Litschig, 2015). The interventions mostly included life skills curricula facilitated in groups of adolescents outside of school, although some were implemented in classrooms during the school day (Carney et al., 2019; Duflo et al., 2014; Edmonds et al., 2016) and the location was unclear for others (Meller & Litschig, 2015). In terms of participants, 12 of the programs were focused on girls, and four included both girls and boys (Carney et al., 2019; Duflo et al., 2014; Johnston & Ksoll, 2017; Meller & Litschig, 2015). Fifteen of the studies provided estimates of effects on girls (GRADE Summary 1; Figure 4.11.1), and one study also estimated the effect for girls and boys combined with interactions by sex (GRADE Summary 2; Figure 4.11.2).

The study that examined combined effects for girls and boys (GRADE Summary 2; Figure 4.11.2), estimated effects for two academic skills, one of which was significant overall (for girls and boys) but showed no indication that girls benefitted more than boys (Johnston & Ksoll, 2017). Given that the life skills component was not the main component in this multicomponent intervention, although the effect size was large, there are serious indirectness concerns.

All programs that measured effects for girls specifically were multi‐component. That is, life skills in either safe spaces or classroom settings was one program strategy implemented along with other strategies, such as financial incentives or mathematics instruction. The additional components varied in both type and number from one study to the next, including: financial literacy (Austrian et al., 2020), health vouchers (Austrian et al., 2020), savings accounts (Austrian et al., 2020; Özler et al., 2020), vocational training (Bandiera et al., 2014, 2019), education support (Buchmann et al., 2016), financial incentives (Buchmann et al., 2016; Hungi & Ngware, 2017; Özler et al., 2020), social entrepreneurship and leadership course (Carney et al., 2019), mentoring (Carney et al., 2019; Edmonds et al., 2016), student business clubs (Carney et al., 2019), school uniforms (Duflo et al., 2014), homework support (Hungi & Ngware, 2017), sensitizing parents and/or community about the importance of girls' schooling (Hungi & Ngware, 2017, Mensch et al., 2019), girl‐friendly schools (Meller & Litschig, 2015), facilitated reading groups with e‐readers (Mensch et al., 2019), girl‐friendly extracurricular activities (Morrell et al., 2014), and interactive satellite instruction in English and Math (Johnston & Ksoll, 2017).

As with other barriers, it is not possible to tease out the effects of life skills from many of these multicomponent programs. However, several of the studies (Ashraf et al., 2018; Buchmann et al., 2016; Duflo et al., 2014; Mensch et al., 2019) were designed in a manner that allows us to assess more directly the effects of their life skills components on education outcomes.

Buchmann and colleagues test three different intervention approaches in a four‐arm RCT: (1) a 6‐month group‐based empowerment program that included life skills as well as basic literacy, numeracy, communication skills, and reproductive health; (2) a conditional incentive (cooking oil) to delay marriage, (3) combined empowerment program and conditional incentive; and (4) control. The life skills/empowerment arm significantly increased grade attainment and the likelihood of being in school. Notably, the arm that combined the empowerment program with the incentive provided no significant additional or separate effect.

A study in Kenya separated out the effects of an HIV education program—both alone and in combination with provision of uniforms (Duflo et al., 2014). The HIV education program trained three teachers in each primary school to help them deliver the national HIV/AIDS curriculum, which focuses on abstinence until marriage. While the HIV education arm demonstrated no effect on grade attainment (two measures) or dropout, this may not be surprising given the generally poor performance of abstinence‐only programs. Notably, while the uniform arm significantly improved grade attainment for girls (both reaching 8th grade and grades completed) the arm that combined the uniforms with the abstinence curriculum had no such impact.

Mensch et al. (2019) conducted an RCT in Zambia to evaluate the effects of access to books on girls' education outcomes. Schools were randomized into three study arms: (1) safe spaces groups that followed an empowerment curriculum plus a modest community engagement component; (2) safe spaces groups with empowerment curriculum, modest community engagement, plus e‐readers and facilitated book groups; and (3) control. Looking only at the safe spaces with empowerment curriculum arm, they found no effect on any of the literacy or numeracy outcomes assessed. While this arm also included community engagement, it is unlikely that community engagement would undermine effects of the empowerment program.

Last, Ashraf et al. (2018) present the results of a three‐arm randomized trial testing the effects of an intervention that taught negotiation skills to 8th grade girls in primary schools in Lusaka, Zambia. This study went further than others in terms of isolating the specific impact of life skills. Girls were randomized into: (1) a negotiation group in which female coaches led six after school sessions over the course of two weeks for groups of about 15–20 girls training them in negotiation and interpersonal communication; (2) a safe space group in which girls participated in the same type of groups, led by the same female coaches, but instead of the negotiation curriculum girls could play games, talk, or do homework; or (3) a control group. The evaluation demonstrated significant effects of the negotiation arm in the expected direction on two education outcomes (enrolment and dropout), but no effect on attendance, English, or math skills. While the safe spaces arm also demonstrated positive effects, they were smaller than those for the negotiation arm, and a mediation analysis using a direct measure of girls' negotiation ability found that it explained a large portion of the treatment effect.

The studies that provide more direct evidence on the effects of life skills on education thus show mixed results. Specifically, three educational attainment and enrolment outcomes were assessed by these studies, with two‐thirds studies demonstrating significant impact on attainment and enrolment. Ashraf et al. (2018) and Buchmann et al. (2016) found predominantly small (often close to null) effects and one medium effect, and Duflo et al. (2014) found no significant effects. We note that the latter were reporting on an abstinence‐only HIV program (Duflo et al., 2014). For academic/cognitive outcomes only two studies (Ashraf et al., 2018; Mensch et al., 2019) measured direct effects of life skills finding no significant beneficial effects on academic or cognitive outcomes. Overall, while life skills education interventions were effective in some contexts, the mixed results and indirectness concerns lead to low confidence in existing evidence. We conclude further research is needed on whether, and if so how, life skills education affects education outcomes for girls.

#### 5.3.11 Inadequate menstrual hygiene management (Barrier 12)

With only four studies that meet our inclusion criteria, we conclude that more research is needed to understand the effects of interventions designed to address inadequate MHM on education outcomes. Three of the four included studies directly tested the effects of MHM interventions. However, the findings were inconsistent and two of the studies had important challenges in design or attrition that make it difficult to accurately assess the effects of these interventions.

Our search identified four studies (six papers) evaluating the effects of interventions that addressed inadequate MHM (see Table 5.12). Those interventions ranged from straightforward provision of menstrual cups (Oster & Thornton, 2009) and/or sanitary pads (Benshaul‐Tolonen et al., 2019; Phillips‐Howard et al., 2016), to training on how to make reusable sanitary pads (Wilson et al., 2012), and provision of sanitary pads as part of a broader intervention including multiple components (Hallfors et al., 2011; Iritani et al., 2016). In contrast to many of the other barriers, the interventions that fell under this barrier were more narrowly targeted: for three of the four studies, addressing MHM was the sole focus (the exception was Hallfors et al., 2011; Iritani et al., 2016). All four studies were experimental, and three out of the four had a low risk of bias. The exception (Wilson et al., 2012) had a high risk of bias due to small cluster size, issues with effective randomization, and self‐reported absenteeism at endline (see Tables 3 and 4). Two of the interventions were conducted in Kenya (both in western Kenya), one in Zimbabwe, and one in Nepal. All four studies had an experimental design.

**Figure 4.12.1:**
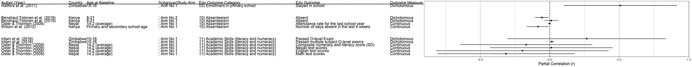
Forest plots showing partial correlation coefficients and 90% confident intervals for direct effects for girls, Barrier 12

Each study reported the effects of interventions on girls (GRADE Summary 1, Figure 4.12.1). As shown in Figure 4.12.1, three of the four studies reported a significant effect of the intervention in the expected direction for at least one outcome (except for Oster & Thornton, 2009), and three out of six papers reported significant effects in the expected direction. None of the six models estimating the effects of these interventions on academic skills found evidence of an effect.

One of the significant effects on absenteeism and school dropout came from a study done in Kenya, the results of which were published in two different papers (Benshaul‐Tolonen et al., 2019; Phillips‐Howard et al., 2016). The study randomized participants to one of three arms: receiving a menstrual cup, receiving sanitary pads, and a control arm. In one paper (Benshaul‐Tolonen et al., 2019), the authors reported a significant effect of participation in the sanitary pad arm on absenteeism (5.4% reduction compared to the control group) along with a small but nonsignificant effect of the menstrual cup arm on absenteeism. However, they note that most of the episodes of absenteeism were among girls reported to have transferred schools, and when these students are removed from the analysis there is no longer an intervention effect.^16^ The same study found no effects of either intervention arm on school dropout (Phillips‐Howard et al., 2016).^17^ Note that the results from the Phillips‐Howard et al. (2016) study are not included in Figure 4.12.1 due to misleading results from the conversion equation.

Oster and Thornton (2009), who found no effects of menstrual cup distribution on absenteeism in Nepal, note that girls in their sample only report missing 1.3 days of school on average over the course of the school year due to menstruation. Therefore, even an effective intervention would be unlikely to have a large impact on education in that setting. However, they found that girls reported increased convenience as a result of the menstrual cups, which may be considered a valuable outcome, despite a lack of effects on self‐esteem, empowerment, gynaecological health, or daily activities (Oster & Thornton, 2009).

Results from a multi‐component intervention in Zimbabwe also showed a significant effect on both age at marriage and school dropout (Hallfors et al., 2011; Iritani et al., 2016). However, we are unable to isolate the role of sanitary pad distribution, which was part of a broader intervention including payment of school fees, uniforms, school supplies (exercise books, pens, soap, underpants), and an adult assigned to monitor school attendance and help as needed. The third study that found significant effects on absenteeism reported on an intervention that provided training on how to make reusable sanitary pads (Wilson et al., 2012). However, as noted, that study received a high risk of bias score.

Given the small number of studies, predominantly small and null effect sizes, and concerns about risk of bias (Wilson et al., 2012) and attrition (Benshaul‐Tolonen et al., 2019; Phillips‐Howard et al., 2016), we find little evidence to support these interventions. However, we have low certainty in these results, and conclude that more research is needed on the effects of interventions designed to address lack of MHM supplies. We are aware of at least two ongoing evaluations of MHM programs, both in Kenya (Muthengi & Austrian, 2018; Zulaika et al., 2019).

#### 5.3.12 Lack of water and sanitation (Barrier 13)

We found some promising evidence that WASH interventions may improve primary school enrolment and attendance for girls. Overall, we find that many of the interventions including a WASH component had a significant effect on education outcomes for girls, especially regarding enrolment and attendance. However, four of the seven identified studies included WASH as one component in a larger intervention and it is difficult to isolate the independent effects of the WASH component. Therefore, we recommend more research to understand the circumstances in which WASH interventions are most likely to improve education outcomes for girls.

**Figure 4.13.1:**
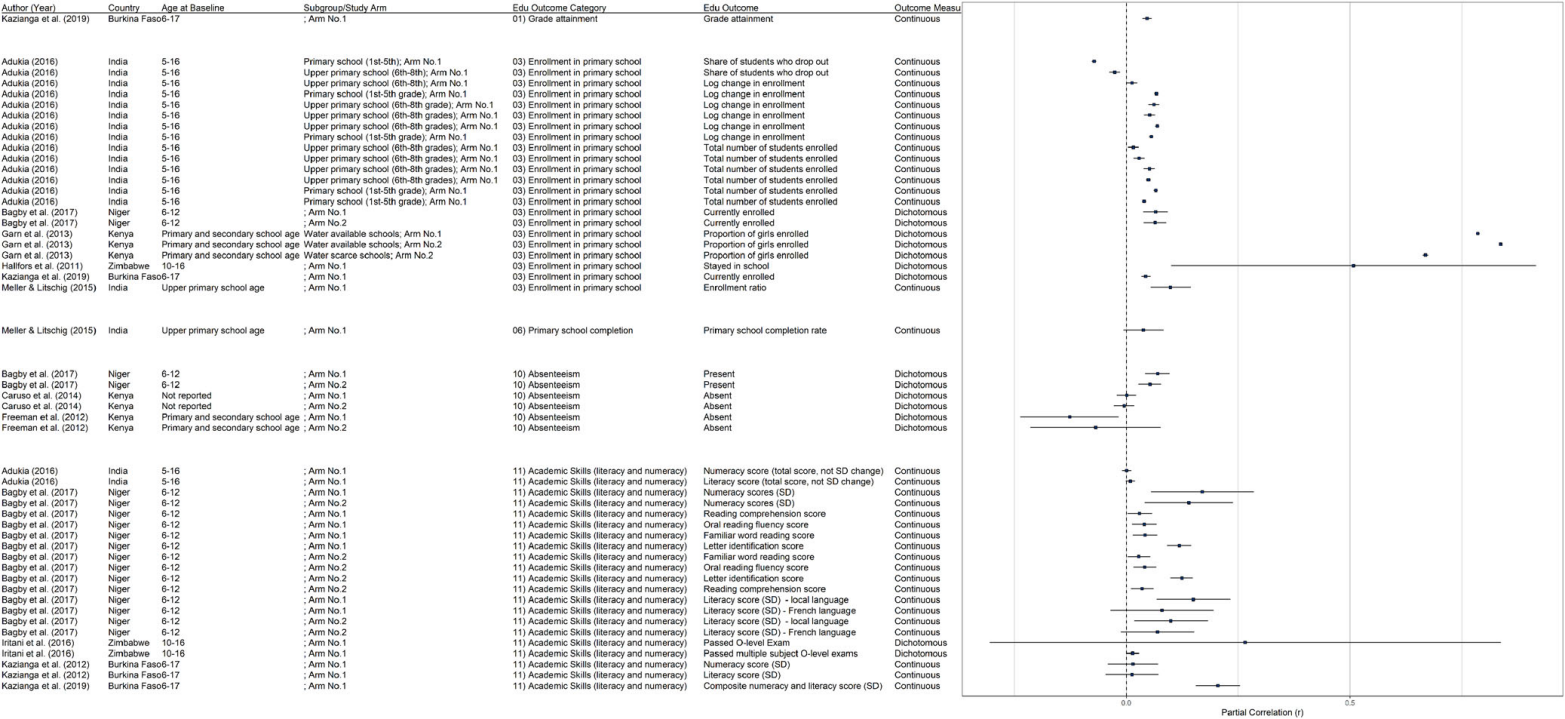
Forest plots showing partial correlation coefficients and 90% confident intervals for direct effects for girls, Barrier 13

**Figure 4.13.2:**
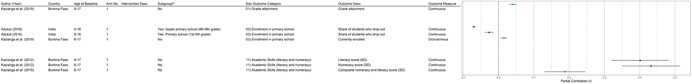
Forest plots showing and 90% confident intervals for direct effects for overall effects for girls and boys combined, Barrier 13* *Green markers indicate that interventions were significantly more effective for girls than boys

We identified seven studies (10 papers) investigating the effects of interventions designed to address lack of water and sanitation on education outcomes (see Table 5.13). Five of the studies had an experimental design, two were quasi‐experimental. Four of the five experimental studies had a low risk of bias; the exception (Freeman et al., 2012; Garn et al., 2013) had some concerns about bias due to missing outcome data and measurement of the outcome. Both quasi‐experimental studies had some concerns about bias, one due to reporting of methods‐specific tests (Adukia, 2016), and the other (Meller & Litschig, 2015) due to concerns about handling of confounding, missing data, and measurement of outcomes. Two of the studies were conducted in Kenya, two in India, and one each in Zimbabwe, Niger and Burkina Faso. All of the included studies (and papers) evaluated the effects of interventions on education outcomes for girls (GRADE Summary 1, Figure 4.13.1). Two studies, described in three papers, reported on overall effects for girls and boys combined and differences by sex (GRADE Summary 2, Figure 4.13.2).

As shown in Table 5.13, interventions ranged from more narrowly defined approaches focused on latrine construction or cleaning, water treatment and/or handwashing (Adukia, 2016; Caruso et al., 2014; Freeman et al., 2012; Garn et al., 2013) to broader multicomponent programs that included WASH activities alongside others, such as support for gender equitable classrooms, literacy training, provision of food and school supplies, and additional training and mentoring activities (Bagby et al., 2017; Hallfors et al., 2011; Iritani et al., 2016; Kazianga et al., 2012; Kazianga et al., 2019; Meller & Litschig, 2015). The former group of studies provides more direct estimates of the effects of WASH interventions on education outcomes, while it is difficult to disentangle the effects of these components from the broader programs in the latter examples.

With regard to school enrolment and attainment, most of the interventions showing significant effects included multiple other components in addition to WASH interventions, making it difficult to isolate the contributions of the WASH components (Bagby et al., 2017; Hallfors et al., 2011; Iritani et al., 2016; Kazianga et al., 2012; Kazianga et al., 2019; Meller & Litschig, 2015). For example, the Burkinabe Response to Improve Girls' Chances to Succeed (BRIGHT) program in Burkina Faso included multiple components, such as separate latrines for boys and girls, take‐home rations, and literacy training, among others. Although the program led to improvements in both enrolment and learning, the authors are unable to estimate the proportion of those improvements attributable to the WASH components. The authors found significantly larger improvements in enrolment for girls than boys at endline (Kazianga et al., 2012); and sustained improvements several years later, including significantly larger effects for girls in enrolment and learning, the latter of which was fully attributable to higher grade progression in intervention schools (Kazianga et al., 2019). Therefore, it is plausible that the separate latrines contributed to the enrolment effects and, indirectly, the learning effects, but whether they did so is unknown. The contribution of WASH components to improvements in enrolment or dropout is perhaps less plausible in other interventions, such as the one evaluated by Iritani et al. (2016) and Hallfors et al. (2011), which included the provision of school fees, uniforms, and school supplies, in addition to the distribution of soap. Similarly, although the National Programme for Education of Girls at Elementary Level (NPEGEL) in India had significant effects on school enrolment for girls (Meller & Litschig, 2015), the authors are unable to isolate the potential contribution of latrine construction from other activities, such as the provision of additional services for girls (e.g., day care centers, flexible timing of classes), remedial courses, and vocational training.

The evaluation of the IMAGINE (IMprove the educAtion of Girls In NigEr) and NECS (Niger Education and Community Strengthening) program in Niger provides somewhat more insight into the contribution of the WASH components (Bagby et al., 2017). The study compared the effects of NECS alone, which focused on activities designed to increase access to high quality education and improve reading achievement in local languages, to NECS in combination with IMAGINE, which focused on school construction, including separate latrines. The authors found that both arms (NECS alone and NECS + IMAGINE) experienced significant improvements in primary school enrolment, attendance, local language scores and math scores, but no effect on French language scores. Impacts on enrolment and attendance were slightly larger for girls than boys in both arms, but the difference was not statistically significant. Overall, the effects were similar between arms, meaning there was no substantial additional benefit from the IMAGINE activities (including latrine construction) above and beyond the NECS activities (Bagby et al., 2017).

The remaining studies identified in our review examined WASH‐focused interventions more directly. SWASH+ was a school‐based WASH program, implemented in government primary schools in four districts, and evaluated using three main study arms: (1) water treatment and hygiene promotion (WT + HP), which included a three day training for teachers on hygiene promotion, behaviour change and water treatment methods, regular follow‐up visits through the school year, handwashing and drinking water containers as well as a 1‐year supply of WaterGuard (a water disinfectant); (2) water treatment and hygiene promotion, along with additional sanitation improvement, focused on provision of latrines up to the government of Kenya standard pupil to latrine ratio, with a maximum of seven latrines; and (3) a control arm (Freeman et al., 2012; Garn et al., 2013). After 2 years of follow‐up, the authors found no overall effect of the intervention on absenteeism, despite a suggestive effect among girls only (Freeman et al., 2012). Further analyses revealed that, within “water scarce” schools, both intervention arms led to significant increases in enrolment, and narrowing gender gaps in enrolment (Garn et al., 2013). They found no effects within “water available” schools. The authors hypothesize several mechanisms driving these effects, including general benefits of having a more appealing school environment, health benefits, privacy, less time spent fetching water for girls, and/or the need for water during menstruation, given more pronounced effects for girls during sixth and seventh grades (Garn et al., 2013).

The study team subsequently explored the added benefit of handwashing and latrine cleaning interventions in schools enrolled in the SWASH+ study that had not receive an improved water source as part of the intervention, had a dry season water source within one km (“water available” schools), and had more than 25% of the school's latrines identified as dirty (Caruso et al., 2014). The study observed improvements in latrine use and handwashing in both arms but saw no effects on absenteeism. The authors hypothesized several reasons for the lack of effect, including that latrine cleaning alone may not have addressed structural issues within latrines that affected use, such as running water, and younger pupils may have needed training in latrine use. Although handwashing improved, additional analyses revealed a nonsignificant reduction in *Escherichia coli* contamination on pupils' hands in a subset of trial schools (Caruso et al., 2014).

The final study that examined the isolated effects of WASH interventions was a quasi‐experimental evaluation of a national latrine construction program in India (Adukia, 2016). The author examined differing effects of unisex versus sex‐specific latrine construction by age group. She observed increased enrolment of pubescent‐age girls, though predominantly when providing sex‐specific latrines, and no effects on learning. However, she found that the construction of any latrine (sex‐specific or not) benefitted younger girls and boys, who may be particularly vulnerable to illness from uncontained waste. The author points out that the findings on younger children underline the fact that the availability of water and sanitation in schools may be important for broader reasons than menstruation. She also notes that, given the lack of effects on learning, investments that increase enrolment and attainment may need to be accompanied by efforts to make schools more effective learning sites (Adukia, 2016).

Overall, when we look only at the studies providing direct estimates of the effects of WASH interventions, the results are somewhat mixed based on outcome. Although insufficient evidence exists to draw strong conclusions, we find some promising evidence that WASH interventions may improve primary school enrolment and attendance for girls in some settings with statistically significant effects ranging from small to large. Less evidence is available on learning outcomes, with only one study directly measuring effects and finding no significant results. Therefore, despite some promising evidence, we find that more research is needed to understand which components of WASH interventions are most likely to be effective in different contexts and populations.

#### 5.3.13 Inadequate school access (Barrier 14)

We find promising evidence that interventions designed to address inadequate school access may improve school enrolment, attainment, and possibly learning for girls. Many of the included interventions were effective at improving school enrolment or attainment for girls, as well as learning outcomes. However, efforts to expand access to school are often part of larger multi‐component initiatives, and much of the existing evidence is unable to isolate the direct effects of the access‐related components on education outcomes. Within the smaller group of studies examining the direct effects of these interventions, some promising findings emerge with regard to effects on school enrolment. The findings on effects on learning are more mixed for girls, but consistently positive for boys and girls combined. More research is needed to understand the circumstances in which interventions that expand access to school are most effective.

**Figure 4.14.1:**
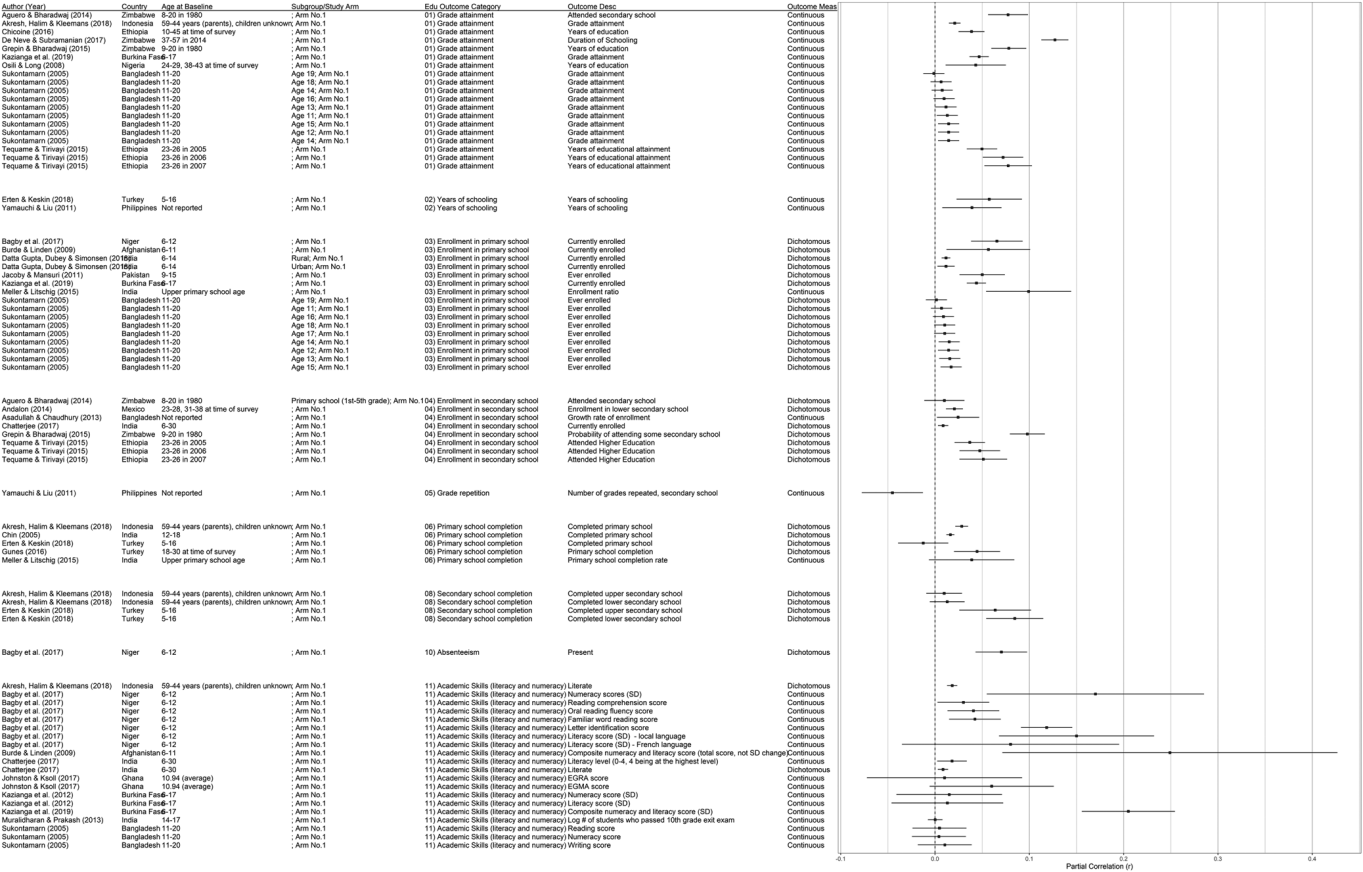
Forest plots showing partial correlation coefficients and 90% confident intervals for direct effects for girls, Barrier 14

**Figure 4.14.2:**
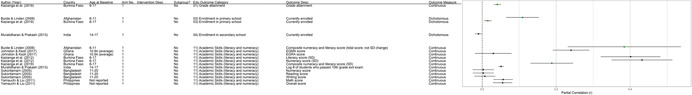
Forest plots showing and 90% confident intervals for direct effects for overall effects for girls and boys combined, Barrier 14*. *Green markers indicate that interventions were significantly more effective for girls than boys

We identified 23 studies (24 papers) examining the effects of interventions designed to address inadequate school access (see Table 5.14). Out of the 23 studies, three were experimental (Bagby et al., 2017; Burde & Linden, 2009; Johnston & Ksoll, 2017) and the remaining 20 were quasi‐experimental. All three of the experimental studies had low risk of bias, as did 12 of the quasi‐experimental studies. The remaining eight quasi‐experimental studies had some concerns regarding risk of bias. Most had concerns in terms of their handling of missing data—largely due to lack of information provided by the authors, while others raised concerns about their ability to effectively address confounding, reporting of tests related to the methods used, and, to a lesser extent, bias in selection of participants or reporting of results (see Tables 3 and 4). All of the included studies reported the effects of interventions for girls (GRADE Summary 1, Figure 4.14.1), while a smaller group reported the effects for girls and boys combined, and differences by sex (GRADE Summary 2, Figure 4.14.2).

As shown in Table 5.14, most of the included studies fall under at least one other barrier, highlighting the challenge of isolating the effects of components aiming to improve access to school from other program activities. Several of the included studies evaluated multicomponent NGO programs that included school construction alongside numerous other interventions (Bagby et al., 2017 in Niger; Kazianga et al., 2012; Kazianga et al., 2019 in Burkina Faso; Asadullah & Chaudhury, 2013 in Bangladesh; Sukontamarn, 2005 in Bangladesh; Burde & Linden, 2009 in Afghanistan). These wide‐ranging interventions all led to improvements in education outcomes for girls, and often effectively narrowed or closed gender gaps in enrolment and attainment. Several studies also found evidence that the interventions improved learning outcomes for girls (Bagby et al., 2017; Burde & Linden, 2009; Kazianga et al., 2019), gains that exceeded those for boys in both cases where the authors report differences (Burde & Linden, 2009; Kazianga et al., 2019). Despite the seeming success of these broad interventions, they provide limited evidence of the specific contribution of individual components to improvements in education outcomes for girls. Nonetheless, they provide useful insights into the importance of addressing school access for girls.

Four of the included studies (Chatterjee, 2017; Chin, 2005; Meller & Litschig, 2015; Datta Gupta et al., 2018) assessed the impacts of multicomponent national programs in India, some of which were better able to isolate the effects of individual components than others. Chin (2005) explored the effects of Operation Blackboard (OB) in India, the country's first major program to address school quality, launched in 1987. Through OB, the government sought to provide all primary schools with a teaching‐learning equipment packet, including blackboards, books, charts, and teachers' manuals. It also sought to provide all primary schools that had only one teacher with a second teacher. The author focuses on the effects of the teacher provision component of OB and finds that, despite inefficiencies in the program's implementation, the distribution of teachers across schools changed and the program led to improvements in primary school completion, especially for girls and the poor. Datta Gupta et al. (2018) investigate the effects of a subsequent package of Indian government programs: the District Primary Education Programme (DPEP) introduced in the mid‐1990s, the Sarva Shiksha Abhiyan (SSA) launched in 2000 and the Mid‐day Meal Program (MDM) universalized in 2001. The reforms were wide‐ranging, including substantial investments in school infrastructure, textbook development, teacher professional development, early childhood education, provision of meals in school, and strengthening community involvement. The authors observed significant increases in school attendance resulting from the reforms, effects that were about twice as strong for girls. Meller and Litschig (2015) evaluated two government programs that were layered onto the SSA program: (1) the National Programme for Education of Girls at Elementary Level (NPEGEL), started in 2003 to provide flexible funding to schools for a range of activities, including small infrastructure projects and making the timing of classes more flexible, among other components; and (2) the Kasturba Gandhi Balika Vidyalaya (KGBV), started in 2004, which set up new boarding schools targeting upper primary out of school girls, in addition to working with communities to identify girls who had dropped out. The authors find that the combined program (NPEGEL + KGBV) led to a significant increase in enrolment for girls, and a reversal of the gender gap in enrolment (Meller & Litschig, 2015). Despite evidence in support of these government initiatives, the fact that they were so wide‐ranging makes it difficult to disentangle the contributions of each component toward improvements in education outcomes for girls. However, Chatterjee (2017) attempted to isolate the effects of the KGBV program, given its focus on school construction for girls. He found that exposed cohorts were more likely to have attended school and perform better on reading tests. He also noted that the independent effects of KGBV on enrolment were positive, but smaller than those in previous combined assessments (including Meller & Litschig, 2015), indicating that enrolment effects may have been largely driven by NPEGEL. Taken together, evidence from several wide‐ranging education initiatives in India supports significant positive effects on enrolment and learning, especially for girls and the poor. It appears that those improvements were driven in part by school construction activities.

Many of the included papers examined the effects of policy changes focusing, in part, on expanding access to school through school construction, AP, compulsory education laws, access to transportation, and/or deregulation. Included papers examined policy changes in Ethiopia (Chicoine, 2016; Tequame & Trivayi, 2015), Zimbabwe (Agüero & Bharadwaj, 2015; De Neve et al., 2017; Grépin & Bharadwaj, 2015), Turkey (Erten & Keskin, 2018; Güneş, 2016), Nigeria (Osili & Long, 2008), Mexico (Andalón et al., 2014); Indonesia (Akresh et al., 2018), and the Philippines (Yamauchi & Liu 2011a, 2011b). Without exception, these policies led to significant increases in enrolment and/or attainment for girls.

In Ethiopia, the 1994 Education and Training Policy (EETP) required public education to be free for grades 1–10, increased the number of public higher education institutions, and deregulated private provision of higher education. Both papers examining this policy found significant increases in enrolment and attainment as a result of the policy (Chicoine, 2016; Tequame & Trivayi, 2015). In Zimbabwe, a 1980 policy reform reduced academic and structural restrictions limiting advancement toward secondary school, including through automatic grade progression to secondary school and a large secondary school construction effort focused on rural areas. All three studies examining this policy change also found significant effects on school enrolment or attainment for girls (Agüero & Bharadwaj, 2015; De Neve et al., 2017; Grépin and Bharadwaj, 2015). In Turkey, two studies (Erten & Keskin, 2018; Güneş, 2016) investigated the effects of the 1997 Compulsory Schooling Law, which increased mandatory school attendance from 5 to 8 years, included restoration of old schools and construction of new schools, recruitment and training of new teachers, purchase and distribution of computers, development of a bus system, and distribution of free books and meals to low‐income students. Both studies found significant increases in educational attainment for girls as a result of this policy (Erten & Keskin, 2018; Güneş, 2016). Osili and Long (2008) also find a significant effect of the Universal Primary Education program in Nigeria, which eliminated school fees for primary education, increased primary school construction and provided teacher training institutions, on attainment for girls. Andalón et al. (2014) found a significant effect of Mexico's National Agreement for the Modernization of Basic Education in 1992—which included extension of compulsory education and construction of public lower secondary schools—on attainment for girls. In Indonesia, Akresh and colleagues (2018) found a significant effect of a national school construction project—which included recruitment, training and pay for teachers to staff the schools—on attainment and literacy for women. Finally, Yamauchi and Liu (2011a, 2011b) investigated the long‐term effects of the Third Elementary Education Project (TEEP) in the Philippines, implemented from 2000 to 2006. TEEP included the following components: school building construction and renovation, textbook distribution, teacher training, school‐based management, and other modules to improve school facilities, as well as a process of decentralizing decision‐making to the school level. The program led to significant improvements in national achievement test scores, and a significant reduction in grade repetition for girls. Unlike many other countries where studies in this review were conducted, the authors note a gender gap in attainment in favor of women in the Philippines, which widened as a result of the TEEP program.

In addition to government reforms, several of the included studies examined the effects of NGO or community schools on education outcomes for girls. Sukontamarn (2005) evaluated the BRAC model, which was operating through more than 30,000 nonformal primary schools at the time of the study. The author found that cohorts exposed to NGO schools, most of which used the BRAC model, had a higher probability of enrolment; the effect was largely explained by higher enrolment for girls in rural areas, especially those in “BRAC target households,” which tend to be among the poorest. The author also found that being enrolled in an NGO school had a positive and significant effect on children's test scores, with no difference by sex. Beyond the presence of the school in a community, the author found support for two mechanisms connecting NGO schools to higher enrolment: a high percentage of female teachers and having parent‐teacher associations (PTAs) (Sukontamarn, 2005). Asadullah and Chaudhury (2013) explored the effects of BRAC‐run primary schools on female enrolment in secondary madrasas. They found that, by targeting out of school children from poor families, BRAC schools led to the feminization (increasing proportion of female vs. male students) of secondary madrasas, more so than public secondary schools. Burde and Linden (2009) also investigated the effects of constructing community‐based schools, but in randomly selected villages in northwest Afghanistan. They found that the program significantly increased enrolment and test scores among all children and led to a narrowing of gender gaps. Enrolment increases were significantly larger among girls, as were improvements in test scores; the latter difference was explained completely by increases in enrolment (Burde & Linden, 2009). While community schools expand access to schools to those who previously did not have access, these studies show that beyond access to school, other components of these schools, including the percent of female teachers, the presence of PTAs, and targeting marginalized communities may contribute to beneficial outcomes.

Bagby and colleagues (2017) evaluate the effects of the IMAGINE and NECS projects, a partnership between the government of Niger and the Millennium Challenge Corporation. The authors compare the combined outcomes with the outcomes from the NECS project on its own. IMAGINE focused on the construction of “girl‐friendly” schools that had three classrooms, housing for three female teachers, a preschool, and separate latrines for boys and girls that were equipped with hand‐washing stations. NECS was a package of activities designed to increase access to high quality education (e.g., borehole construction and maintenance, mobilization of student governance structures, promotion of gender‐equitable classrooms and student leadership) and improve reading achievement in local languages (train and support teachers in new methods of reading instruction, develop reading materials in local languages). The authors found that both arms (NECS alone and NECS + IMAGINE) led to improvements in enrolment, attendance, and academic skills for girls, but they observed no added benefit of IMAGINE beyond the NECS activities. They also found that IMAGINE had a negative economic rate of return given its high costs and minimal added benefits. In this case, the authors find limited benefit of a school construction program, in the context of a broader government initiative focused on expanding access to quality schooling.

The BRIGHT program in Burkina Faso also constructed “girl‐friendly” primary schools, and provided additional amenities, such as take‐home rations and textbooks, and literacy training (Kazianga et al., 2012). The prototype BRIGHT school included separate toilets for girls and boys, a borehole with a pump to provide clean water, teacher and student desks and chairs, and a playground. The program had significant effects on enrolment for primary age children, and on test scores. They found that enrolment increased significantly more for girls than boys, which was fully explained by the “girl‐friendly” amenities, rather than simply constructing the schools, which also had an effect for both groups (Kazianga et al., 2012). The authors found sustained effects on enrolment and test scores 7 years after the intervention, and larger effects for girls than boys; gender gaps in educational outcomes closed in intervention villages (Kazianga et al., 2019). These results point to the potential added value of “girl‐friendly” amenities in school construction initiatives when aiming to close gender gaps in enrolment or attainment.

The remaining studies included under this barrier evaluated the effects of somewhat more narrowly focused interventions, including providing bicycles to girls enrolled in secondary school in India (Muralidharan & Prakash, 2013), providing distance learning in Ghana (Johnston & Ksoll, 2017), or exploring the effects of social barriers on access to school in Pakistan (Jacoby & Mansuri, 2011). Although these interventions are not directly comparable, they provide evidence of the potential benefits of each approach.

Muralidharan and Prakash (2013) study the effects of a program in Bihar state in India in 2006 that provided secondary school girls with bicycles to improve their access to school. They find that the program significantly increased girls' enrolment in secondary school and reduced the gender gap in secondary school enrolment. Most of the increases took place in the villages where secondary schools were farther away, and the program was more cost effective than a comparable conditional cash transfer program. The authors point out that programs such as these that improve school access might be complementary to school construction programs given the added benefits of mobility and lower costs (Muralidharan & Prakash, 2013).

Johnston and Ksoll (2017) report on the results of a cluster randomized trial testing whether remote instruction in rural Ghanaian primary schools improved student outcomes. The program provided solar power and satellite technology to 70 randomly selected primary schools and broadcast daily math and English lessons. Professionally trained teachers taught the lessons through video conference from Accra, while in‐person teachers from the rural schools managed the classrooms. The authors find that, 2 years after implementation, the program significantly improved numeracy and literacy scores for students. They argue that these improvements were likely driven by increased instructional quality rather than increased instruction time, as well as increases in the teacher‐student ratio.

Jacoby and Mansuri (2011) conducted a quasi‐experimental study to understand the effects of social barriers to school enrolment in Pakistan, which they define as communal heterogeneity. Specifically, they examine girls' ability to travel to schools outside of their communities due to the custom of purdah, or female seclusion, and caste differentiation, which may lead to discrimination against low caste groups by high caste groups. They find that, controlling for distance to school, the odds of enrolling are significantly lower for girls (but not boys) who would need to cross settlement boundaries to attend, and for low caste girls and boys residing in high caste‐dominant communities. Conversely, they find that girls who have access to schools within their settlement, and low‐caste boys and girls who have access to a school in a low‐caste dominant area, are significantly more likely to enroll.

Taken together, a substantial body of evidence exists of the effects of policies or interventions designed, in part, to address lack of access to school for girls. One challenge with this evidence is that the wide‐ranging nature of government reforms, which often include activities like school construction as one of several pieces, makes it difficult to isolate the contributions of those components. Further, beyond whether school construction is effective, this body of evidence raises important questions about how the nature of school facilities (e.g., “girl‐friendly” characteristics), placement of those facilities (e.g., in rural and/or homogenous areas), and staffing and resources given to those facilities may further influence the impact of high quality and gender‐sensitive education. Existing evidence also highlights some alternative approaches to facilitating access to education, including distributing bicycles and remote learning. Given this body of evidence, we conclude that efforts to expand access to school are promising approaches to improving enrolment and attainment—with significant effects of small and medium size among the studies that more directly measured the impact of these programs—and, possibly, learning for girls, though this had more inconsistent results. However, more research is needed to understand which components are most effective in different populations and evaluate the cost‐effectiveness of different approaches.

#### 5.3.14 Poor policy/legal environment (Barrier 15)

Overall, when assessing the interventions that also focus on improving access to school, largely through school construction, we find promising evidence of the effects of policies and laws aiming to improve education outcomes for girls. However, when we set aside those policies, the effects of which may be fully explained by school construction, the remaining policies are varied, and the findings inconsistent, and we conclude that more research is needed on which policies might be most effective at improving girls' education outcomes.

**Figure 4.15.1:**
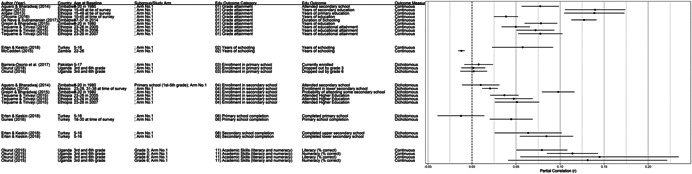
Forest plots showing partial correlation coefficients and 90% confident intervals for direct effects for girls, Barrier 15

**Figure 4.15.2:**
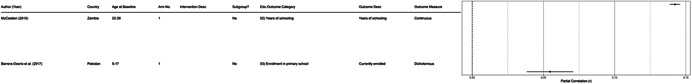
Forest plots showing and 90% confident intervals for direct effects for overall effects for girls and boys combined, Barrier 15* *Green markers indicate that interventions were significantly more effective for girls than boys

We identified twelve studies (thirteen papers) examining the effects of interventions addressing poor policy or legal environments (see Table 5.15).^18^ Eleven of the studies were quasi‐experimental. The one experimental study (Barrera‐Osorio et al., 2017) had a low risk of bias. Seven of the quasi‐experimental studies had a low risk of bias. The remaining quasi‐experimental studies (Argaw, 2013; Chicoine, 2016; Erten & Keskin, 2018; Güneş, 2016; Tequame & Trivayi, 2015) had some concerns, largely related to failure to report information on how they handled missing data, or results from methods‐specific tests (see Tables 3 and 4). Eleven of the included studies (12 papers) estimated effects of the interventions on girls (GRADE Summary 1, Figure 4.15.1), while a smaller group estimated differences by sex (GRADE Summary 2, Figure 4.15.2).

As shown in Table 5.15, we find substantial overlap between the interventions included in the previous group (those designed to improve access to school) and in this group (policies and laws designed to improve education for girls). Eight of the twelve studies were also included in the previous barrier, all of which found significant effects of governments policies that included school construction along with other policy changes, and all of which led to improvements in girls' educational attainment. Two studies (Chicoine, 2016; Tequame & Trivayi, 2015) found a significant effect of the 1994 Education and Training Policy in Ethiopia, which required public education to be free for grades one through 10, increased the number of public higher education institutions, and deregulated private provision of higher education, on attainment in Ethiopia. In Zimbabwe, a 1980 policy reform reduced academic and structural restrictions limiting advancement toward secondary school, including through automatic grade progression to secondary school and a large secondary school construction effort focused on rural areas. All three studies examining this policy change also found significant effects on school enrolment or attainment for girls (Agüero & Bharadwaj, 2014; De Neve et al., 2017; Grépin & Bharadwaj, 2015). Andalón et al. (2014) found a significant effect of Mexico's National Agreement for the Modernization of Basic Education in 1992, which included extension of compulsory education and construction of public lower secondary schools, on attainment for girls. In Turkey, Güneş (2016) investigated the effects of the 1997 Compulsory Schooling Law, which increased mandatory school attendance from 5 to 8 years, in addition to other components (restoration of old schools and construction of new schools, recruitment and training of new teachers, etc.). The author found significant increases in educational attainment for girls as a result of this policy (Güneş, 2016).

This section also includes evaluations of several interventions that were not included in the previous section. Argaw (2013) evaluates the effects of a different component of the 1994 Education and Training Policy in Ethiopia, one that was focused on the introduction of mother tongue instruction, the implementation of which varied across regions. The author found that the policy led to a significant increase in primary and secondary attainment for girls. Okurut (2015, 2018) investigated the effects of AP policies in primary school in Uganda. The AP policy was implemented as part of a broader education strategy aiming to improve efficiency in the system. As part of the policy, remedial classes were available before and after school for academically weak students. The author found significant effects of the AP policy on literacy and numeracy in primary 3 and primary 6, larger effects in rural areas compared to urban areas, and similar effects for boys and girls (Okurut, 2015). In addition, the author found some effect on dropout among male and female students in rural areas in primary grade three, but not in urban areas and not in primary grade six (Okurut, 2018).

McCadden (2015) evaluated the effects of a 1997 School Re‐Entry Policy (REP) in Zambia on education outcomes for girls. The author finds that, although educational attainment increased for adolescent mothers following the policy, it did not increase as much as for females overall, or for males overall, reflecting larger trends taking place in the country. The author points out that, in addition to this policy, efforts are needed to address the social, financial and practical challenges in returning to school after giving birth.

Finally, Barrera‐Osorio and colleagues (2017) examined the effects of a program in Pakistan that provided either a “gender‐uniform” subsidy to schools (350 rupees per student) or a “gender‐differentiated” subsidy to schools (350 rupees for each male student, 450 rupees for each female student). The program targeted newly created publicly funded private primary schools in rural underserved districts. The authors found no difference in girls' enrolment based on treatment arm, but they did find a significant increase in enrolment overall (for girls and boys combined).

Overall, when assessing the interventions that also focus on improving access to school, largely through school construction, we find promising evidence of the effects of policies and laws aiming to improve education outcomes for girls. However, when we set aside those policies, the effects of which may be fully explained by school construction, the remaining policies are varied, and the findings inconsistent, including null findings and beneficial effects of medium size, though in some settings these had wide CIs. Therefore, we conclude that more research is needed on the effects of policies and laws, especially those that do not include a school construction or tuition elimination component.

#### 5.3.15 Inability to afford tuition and fees (Barrier 16)

The impacts of programs and policies that directly addressed the inability to afford tuition and fees appear effective. However, in the four studies that looked at differential effects on girls and boys, there was no indication that gender gaps were reduced in these settings. Note that we excluded cash transfer interventions, or other types of transfers to the household, so these results apply to transfers directly to schools.

We identified 21 studies and 22 articles that examined the effects of interventions designed to address an inability to afford tuition and fees (Table 5.16). Of these, most had a low risk of bias, while three studies (Blimpo et al., 2016; Cho et al., 2019; Chyi & Zhou, 2010) had “some concerns,” and two (Hungi & Ngware, 2017; Lucas & Mbiti, 2010) had a “high” risk of bias including because of high risk of confounding and missing data. Fifteen studies were quasi‐experiments, with the remaining six being experimental studies.

Twelve of the studies evaluated multi‐component interventions (Agüero & Bharadwaj, 2014; Chicoine, 2016; Cho et al., 2019; Chyi & Zhou, 2010; De Neve & Subramanian, 2017; Grant, 2015; Grépin and Bharadwaj, 2015; Güneş, 2016; Hallfors et al., 2011; Hungi & Ngware, 2017; Iritani et al., 2016; Mbiti et al., 2019; Osili & Long, 2008), which included other components such as school construction, recruitment of new teachers, teacher training, school materials provision, academic support, life skills training, automatic grade progression, and cash transfers. The nine studies of single component programs ranged from free primary education policies (Grogan, 2009; Keats, 2018; Lucas & Mbiti, 2010; Makate, 2016), and tuition and/or fee waivers programs paid directly to the school (Adelman et al., 2017; Barrera‐Osorio et al., 2017; Blimpo et al., 2016; Duflo et al., 2019; Hermida, 2014).

**Figure 4.16.1:**
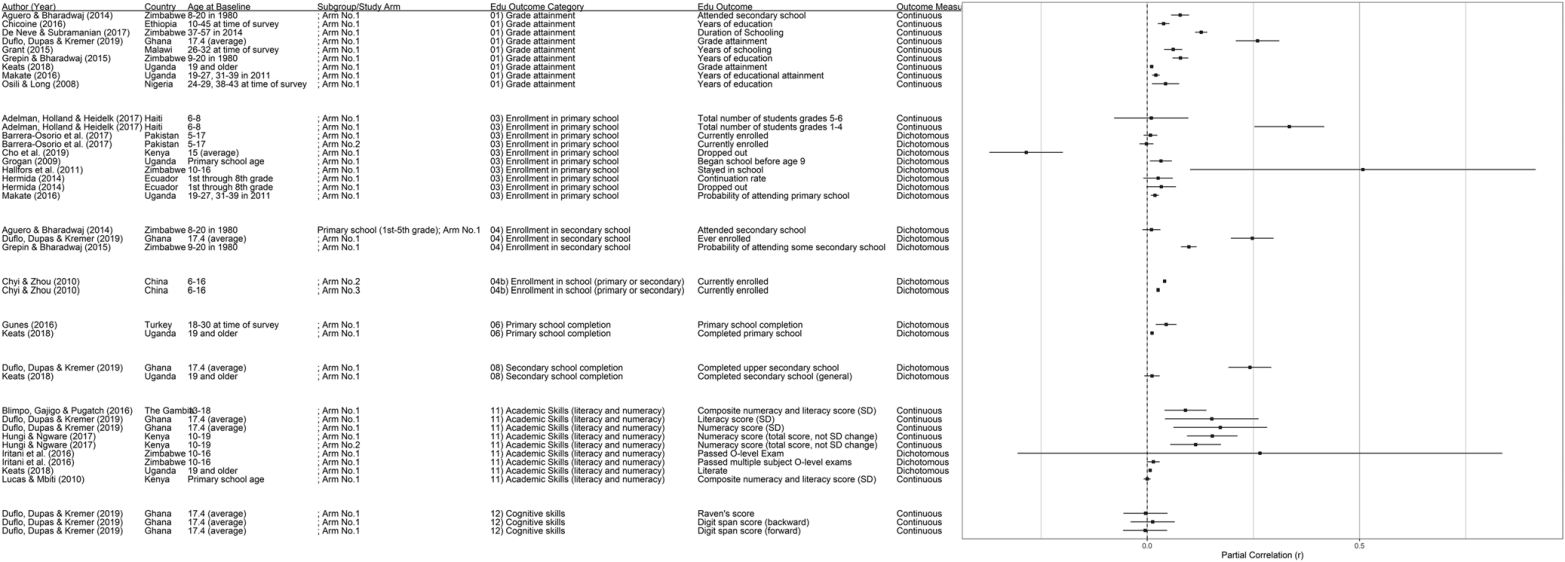
Forest plots showing partial correlation coefficients and 90% confident intervals for direct effects for girls, Barrier 16

Twenty of these assessed outcomes among girls (GRADE Summary 1; Figure 4.16.1). Focusing on studies that provide direct evidence, for the six enrolment and attainment outcomes we find that a majority of these studies (5/7) find at least one significant beneficial effect, including both large and small effect sizes. For academic skills most studies that directly assess effects of providing tuition and fees find at least one significant beneficial effect, ranging from small (close to null) to medium in size. The study that measured cognitive outcomes did not find a significant beneficial effect.

**Figure 4.16.2:**
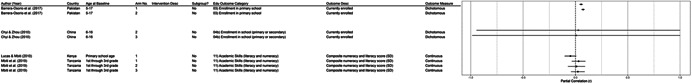
Forest plots showing and 90% confident intervals for direct effects for overall effects for girls and boys combined, Barrier 16* *Green markers indicate that interventions were significantly more effective for girls than boys

As for gender differential effects, one out of four studies and papers (Barrera‐Osorio et al., 2017; Chyi & Zhou, 2010; Lucas & Mbiti, 2010; Mbiti et al., 2019) found significant effects in the expected direction for children overall (GRADE Summary 2, Figure 4.16.2). There were no gender differences for any of the effects. Only two of the studies that looked at outcomes for girls and boys combined provided direct evidence and only one of them found a significant effect (medium size) in the hypothesized direction, thus we have very low confidence in this evidence.

Overall, interventions and policies designed to address the inability to afford tuition and fees seem to be effective in improving girls' schooling outcomes However, we found, albeit with very low confidence, that these programs have not significantly reduced gender gaps in the settings where differential effects on girls and boys were assessed.

#### 5.3.16 Inability to afford school materials (Barrier 17)

While findings suggest that programs that address the cost of school materials, at least as a part of the overall interventions, are promising, we have greater confidence in results for enrolment and attainment outcomes than for academic skills.

**Figure 4.17.1:**
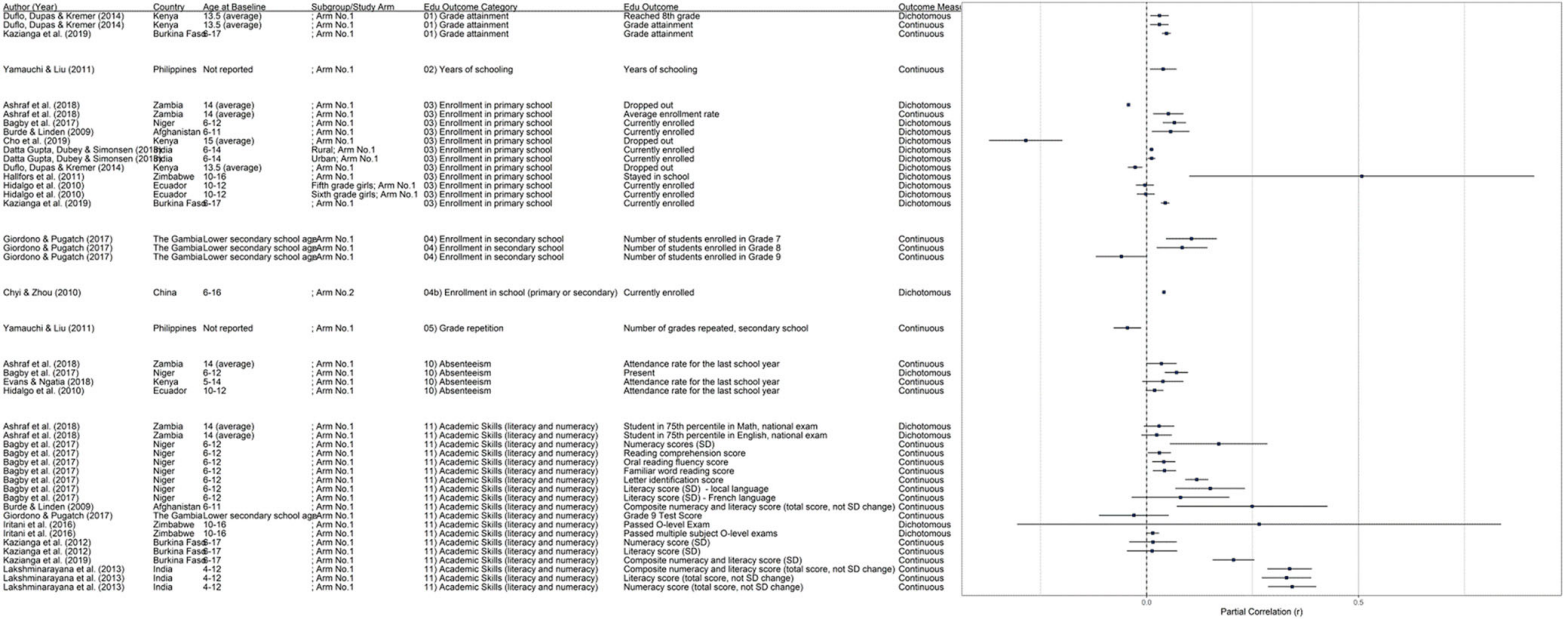
Forest plots showing partial correlation coefficients and 90% confident intervals for direct effects for girls, Barrier 17

Fourteen studies (16 articles) attempted to address an inability to afford school materials (see Table 5.17). Eight studies had a low risk of bias, and six (Cho et al., 2019; Chyi & Zhou, 2010; Datta Gupta et al., 2018; Giordono & Pugatch, 2017; Kazianga et al., 2012; Kazianga et al., 2019; Yamauchi & Liu, 2011a, 2011b) had some concerns. All 14 reported on effects of these interventions on girls, with 12 studies demonstrating at least one significant effect in the expected direction (GRADE Summary 1, Figure 4.17.1). Among these only four studies (Duflo et al., 2014; Evans & Ngatia, 2018; Giordono & Pugatch, 2017; Hidalgo et al., 2010) report the results of a single‐component intervention, including the provision of school uniforms to students (Duflo et al., 2014; Evans & Ngatia, 2018; Hidalgo et al., 2010) and a scholarship program covering school fees and expenses such as books, uniforms, shoes, etc. (Giordono & Pugatch, 2017). The other studies included components such as school construction (Bagby et al., 2017; Datta Gupta et al., 2018; Yamauchi and Liu, 2011a, 2011b), academic support (Cho et al., 2019; Hallfors et al., 2011; Iritani et al., 2016; Lakshminarayana et al., 2013), sanitary products (Hallfors et al., 2011; Iritani et al., 2016), in‐school feeding (Datta Gupta et al., 2018), and negotiation training and mentoring (Ashraf et al., 2018; Giordono & Pugtach, 2017), among others.

Looking at the studies that measured the effects of providing school materials directly, most found a significant effect for at least one attainment or enrolment outcome (Duflo et al., 2014 (small effects); Giordono & Pugatch, 2017 (medium effect); Hidalgo et al., 2010 (no significant effect). Evans and Ngatia (2018) found no significant effects for absenteeism. For academic skills, only Giordono and Pugatch (2017) measured this and found no significant effects.

**Figure 4.17.2:**
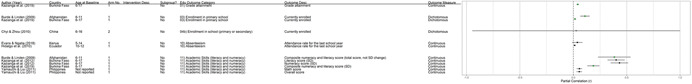
Forest plots showing and 90% confident intervals for direct effects for overall effects for girls and boys combined, Barrier 17* *Green markers indicate that interventions were significantly more effective for girls than boys

For the gender differential results (GRADE Summary 2, Figure 4.17.2) only two studies (Evans & Ngatia, 2018; Hidalgo et al., 2010) implemented single‐component interventions, which were both uniform provision. The other studies implemented multi‐component programs that included school construction (Burde & Linden, 2009; Kazianga et al., 2012; Kazianga et al., 2019), tuition waivers and caps (Chyi & Zhou, 2010), gender‐separate latrines, and in‐school feeding (Kazianga et al., 2012; Kazianga et al., 2019). For the studies that measured the effects directly, 1/2 studies found a small, significant effect in the expected direction for girls and boys combined, but no differential effects by gender (Evans & Ngatia, 2018).

In general, few studies implemented interventions designed to strictly test the causal effects of access to school materials on education outcomes for girls. While interventions that contain school materials components are promising for enrolment and attainment, for learning outcomes more research is needed given the small number of direct studies measuring academic skills and inconsistent effects.

#### 5.3.17 Lack of adequate food (Barrier 18)

Overall, we find evidence that interventions addressing lack of food may be effective at improving school enrolment, attainment, and attendance for girls. The results on learning were mixed. More research is needed to isolate the effects of these interventions, and to clarify the circumstances in which programs that address inadequate food access are most likely to be effective.

**Figure 4.18.1:**
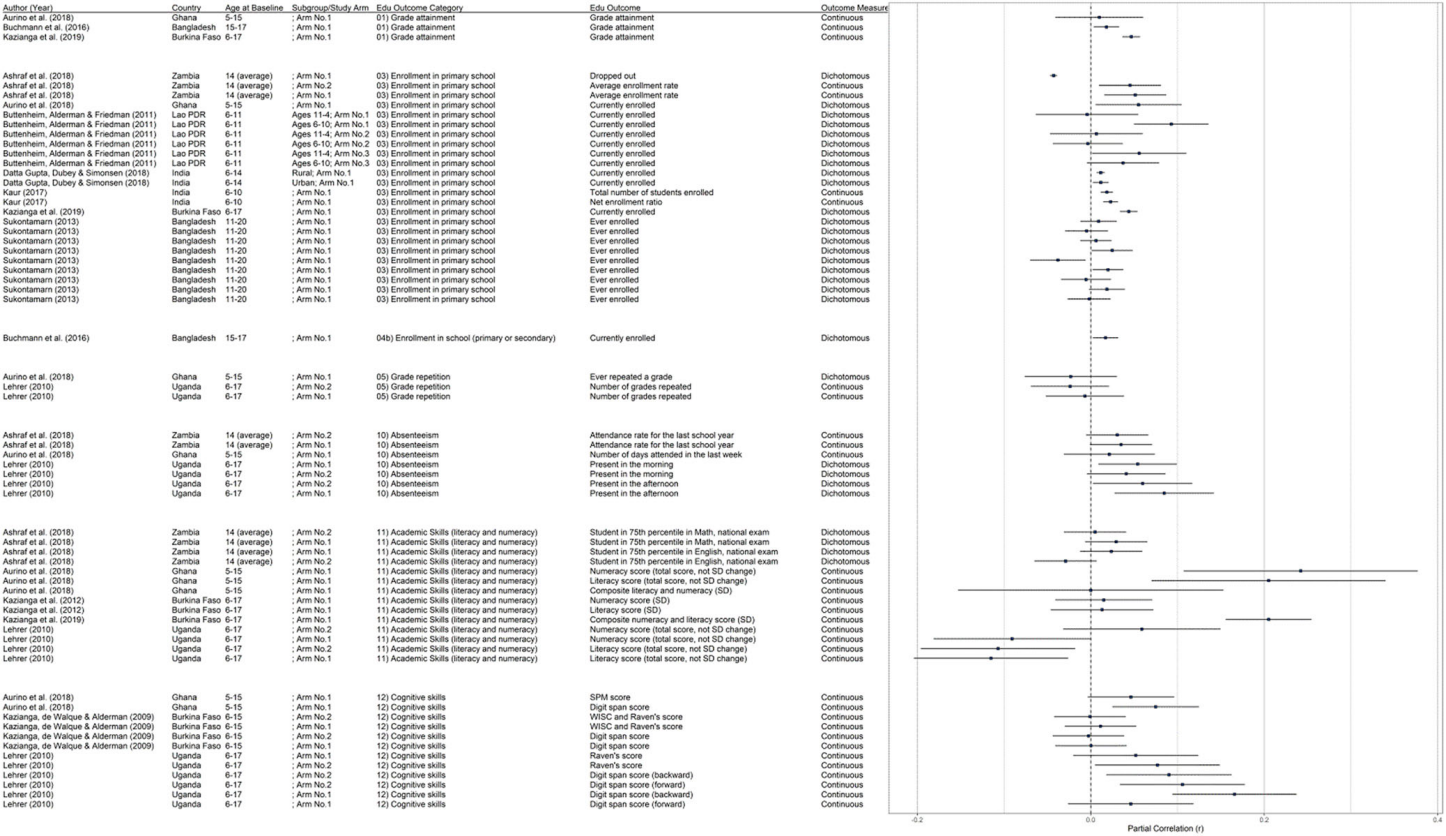
Forest plots showing partial correlation coefficients and 90% confident intervals for direct effects for girls, Barrier 18

**Figure 4.18.2:**
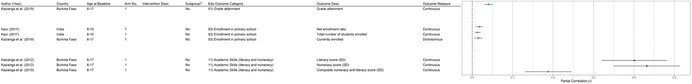
Forest plots showing and 90% confident intervals for direct effects for overall effects for girls and boys combined, Barrier 18* *Green markers indicate that interventions were significantly more effective for girls than boys

Ten studies (11 papers) addressed lack of adequate food (see Table 5.18). Five studies were experimental, all with a low risk of bias. Out of the five quasi‐experimental studies, two had low risk of bias and three had some concerns, largely related to lack of information in the papers about handling of missing data or other sources of bias (see Tables 3 and 4). All 10 studies estimated effects of the interventions for girls (GRADE Summary 1, Figure 4.18.1). Only two studies (Kaur, 2017; Kazianga et al., 2012; Kazianga et al., 2019) reported both overall and gender‐differential effects of school feeding programs on education, both of which found significant effects (GRADE Summary 2, Figure 4.18.2). Several other studies reported significantly larger effects for girls than boys but did not share results of tests for interactions (Aurino et al., 2018; Datta Gupta et al., 2018; Sukontamarn, 2013).

Many of the interventions included in this group were single component (see Table 5.18). These studies examined per‐student payments to local caterers for meals in Ghana (Aurino et al., 2018), in‐school feeding in India (Kaur, 2017), free monthly food conditional on school enrolment in Bangladesh (Sukontamarn, 2013), and in‐school feeding plus take‐home rations in Lao PDR (Buttenheim et al., 2011), Burkina Faso (Kazianga et al., 2009), and Uganda (Lehrer, 2010).

Three studies (Buttenheim et al., 2011; Kazianga et al., 2009; Lehrer et al., 2010) compared the effects of two approaches—on‐site school feeding versus take‐home rations conditional on attendance—on education outcomes. Two of those studies, conducted in northern rural Burkina Faso (Kazianga et al., 2009) and internally displaced person camps in Uganda (Lehrer, 2010) found significant improvements in girls' enrolment and/or attendance from both programs, while effects on cognition and learning varied. The third study, conducted in the Lao People's Democratic Republic, did not find evidence of effects. The authors note that districts with higher levels of baseline enrolment selected into the program, which might explain the null findings, and that the high costs of travel and food delivery make these programs particularly challenging in the study context (Buttenheim et al., 2011).

The remaining single component studies examined the effects of government programs in India, Bangladesh, and Ghana. Aurino and colleagues (2018) found that the government of Ghana's School Feeding Program led to gains in enrolment and several measures of learning for girls. Sukontamarn (2013) found that the government of Bangladesh's Food for Education program led to improvements in enrolment, especially for boys, with some variations based on household characteristics. Last, Kaur (2017) found that the Indian government's Mid‐day Meal Scheme, described as the largest school feeding program in the world, increased primary enrolment, especially for girls and other disadvantaged populations.

Other included interventions, however, had multiple components, making it more difficult to isolate the effects of food provision on outcomes. For example, Datta Gupta (2018) took a broader approach than Kaur (2017) by investigating the effects of a package of Indian government programs: the DPEP introduced in the mid‐1990s, the SSA launched in 2000 and the MDM. The reforms were wide‐ranging, including substantial investments in school infrastructure, textbook development, teacher professional development, early childhood education, provision of meals in school, and strengthening community involvement. The authors observed significant increases in school attendance resulting from the reforms, effects that were about twice as strong for girls.

Ashraf et al. (2018) evaluated a program in Zambia that trained 8th grade girls in negotiation skills, provided safe space with female mentors, and offered girls information about returns to education. Meetings with mentors included daily lunches in both intervention arms. They found small effects of the safe spaces group on its own on enrolment, but significantly stronger effects of the arm including negotiation skills. Similarly, Buchmann and colleagues tested three different intervention approaches in a four‐arm RCT: (1) a 6‐month group‐based empowerment program that included life skills as well as basic literacy, numeracy, communication skills, and reproductive health; (2) a conditional incentive (cooking oil) to delay marriage; (3) combined empowerment program and conditional incentive; and (4) control. The life skills/empowerment arm significantly increased grade attainment and the likelihood of being in school. Notably, the arm that combined the empowerment program with the cooking oil incentive provided no additional or separate effect. Similarly, as described in previous sections, the BRIGHT program in Burkina Faso constructed “girl‐friendly” primary schools, and provided additional amenities, such as take‐home rations and textbooks, and literacy training (Kazianga et al., 2012). The program had significant effects on enrolment for primary age children, and on test scores. The authors found that enrolment increased significantly more for girls than boys, as well as sustained effects on enrolment and test scores seven years after the intervention (Kazianga et al., 2019).

In sum, we find evidence that these interventions are effective at improving girls' enrolment, attainment, and attendance, with the significant effects ranging from small to medium size. The results on learning were mixed, including null to medium effects and one large effect size. More research is needed to clarify the circumstances in which programs that address inadequate food access are most likely to be effective.

## 6 DISCUSSION

### 6.1 Summary of main results

In an attempt to parse out as clearly as possible which interventions affected which barriers, we examined results by intervention group (based on barrier they were designed to address) and specific outcome (e.g., primary school completion) using GRADE assessments. This resulted in an important granular assessment but a small number of studies for every barrier‐outcome pairing. We thus combined the outcomes into two groups—those related to enrollment/attainment and those related to academic skills—to capture overall patterns. Based on these aggregated GRADE results, we find that:

- Effective interventions exist to address three gender‐related barriers: inability to afford tuition and fees, lack of adequate food, and insufficient academic support.
- Promising interventions exist to address three gender‐related barriers: lack of water and sanitation, inadequate school access, and inability to afford school materials.
- More research is needed on the effects of interventions designed to address the remaining 12 gender‐related barriers to education for girls. Some of these did not have enough directly relevant research to draw conclusions with high confidence, such as lack of support for girls' education, child marriage and adolescent pregnancy, SRGBV, lack of safe spaces and social connections, lack of teaching materials and supplies, inadequate sports programs for girls, inadequate health and childcare services, and inadequate MHM. Other barriers had heterogenous effects, and while interventions addressing these barriers may be effective in some settings, more research is needed. This sub‐group includes lack of information on returns to education/alternative roles for women, gender‐insensitive school environment, inadequate life skills, and poor policy/legal environment.

We find substantial gaps in the evidence. Several gender‐related barriers to girls' schooling are under‐examined. Eight barriers have fewer than 10 evaluations, and five barriers—child marriage and adolescent pregnancy, SRGBV, inadequate sports programs for girls, inadequate health and childcare services, and inadequate MHM—have fewer than five evaluations and thus need more research to understand whether and how they work. Also, as discussed below, nearly half of programs implemented in our included studies are multi‐component, and thus many addressed more than one barrier. Yet many evaluations were not designed to tease out the effects of individual components. As a result, for some barriers for which substantial evidence exists on interventions (e.g., inadequate life skills education, lack of safe spaces), we are unable to draw conclusions about effects on education outcomes. Further, the combination of components varies between studies, with few comparable interventions, further limiting our ability to identify packages of interventions that work well.

In addition to the question of whether interventions were effective or promising for girls, we are also interested in understanding the extent to which interventions narrowed or closed gender gaps in settings where girls were at a disadvantage. Interventions that narrow or close gender gaps are presumably doing so by addressing gender‐related barriers to schooling. Overall, we found too few studies to provide conclusive insights into whether the interventions either reduced gender gaps and/or addressed gender‐related barrier(s) to schooling. We should note, however, that even in cases where the authors found no significant difference in effects for boys and girls, the barrier being addressed may still have a gendered component. Those interventions might have effectively addressed a shared barrier for girls and boys (e.g., by improving pedagogy) without effectively addressing gender‐related components (e.g., girls' hesitation to speak up in class). A more gender‐responsive intervention might both improve outcomes for girls and boys and narrow gender gaps. A lack of differential effects of interventions by gender, therefore, is difficult to interpret as it may signify multiple dynamics. It could be that the barrier is not, in fact, gender‐related, or that the intervention did not address the gender‐related barrier (but did address other non‐gendered barriers), or that the barrier is gender‐related in some settings but not others. More research is needed on the question of which interventions narrow gender gaps, beyond improving outcomes for girls, in settings where inequalities exist.

#### 6.1.1 Themes and questions emerging across barriers

In addition to this overall assessment of the evidence, there are several notable research gaps and observations at the level of individual barriers and intervention types. In the table below we have summarized key themes that emerged across this body of evidence and provided examples of remaining research questions related to those themes.

**Table:** 

Theme	Examples of remaining questions
Approaches aiming to shift gender norms and attitudes	Whether and how do these interventions contribute to improved education outcomes? Are they a necessary precursor to program implementation, and do such efforts become less critical as behavior shifts in response to the program? Is it an individual's—for example a parent's—perception of community norms, or their individually held attitudes that are more likely to drive changes in decisions about girls' schooling? Are multi‐level programs—those working at other levels of the ecosystem in addition to the girl herself—more effective than programs working just with girls?
Interventions addressing enrolment or attainment versus learning	Do gender gaps exist with regard to learning, and if so, is it harder to find interventions that close those gaps? Or is it simply that gender gaps are more common in enrolment and attainment, so interventions are more likely to narrow those gaps? Do interventions to shift gender norms primarily impact education outcomes such as enrolment and attainment or are there pathways by which learning outcomes also improve?
Addressing barriers that fall outside the formal school system	How might programs aiming to address barriers, such as child marriage and adolescent pregnancy, affect education outcomes? What levels—or combination of levels—of the ecosystem (e.g., school, community, household) are most critical to intervene upon to reduce gender‐based violence?
Combining and prioritizing intervention components	Is there a core essential package of interventions to address gender‐related barriers to schooling for girls? How should program implementers or policymakers prioritize when choosing between intervention components? Are certain combinations of components important for success, for example, teacher training combined with provision of school materials?
What is the purpose of safe spaces interventions?	Are safe space interventions a platform, such as schools, for the delivery of effective content, training, or incentives, or should they be considered an intervention on their own? If there is an inherent benefit to safe spaces, can those benefits be replicated and scaled through schools?
What is the theory of change behind common approaches to improving girls' education?	Whether and how are sports programs for girls distinct from other extracurricular activities? What are the ways in which inadequate healthcare is most likely to affect education outcomes for girls? What are the ways in which gender‐insensitive school environments most likely to affect girls' education outcomes?
Who should be reached by these interventions?	Are toilets important for students of all ages, or just adolescents? Which students need to be reached most by community schools? Which types of financial transfers (e.g., tuition and fees vs. providing school materials) work best for different groups? Which barriers are most important for primary school enrolment and completion versus secondary school enrolment and completion? How does this vary across settings?
How should program components be implemented?	What is the optimum duration and exposure for different interventions? Which life skills—negotiation, grit, agency, critical thinking, etc.—matter for which outcomes? How key are roles of facilitators such as mentors in determining the success of nonformal education interventions?

While researchers have begun to investigate many of these questions, more work is needed to bring clarity to intervention design and prioritization. Our review aims to answer a fairly narrow question about which interventions have been shown to work in certain contexts, while these broader questions about how and why interventions might work are equally essential to improving outcomes for girls.

### 6.2 Overall completeness and applicability of evidence

This systematic review highlights the important pattern of implementing, and evaluating, multicomponent interventions designed to address gender‐related barriers to schooling. While for some barriers (inadequate MHM, inability to afford tuition and fees, lack of adequate food) the majority of studies test the direct effects of single component interventions, this is not the case for most barriers. This is perhaps appropriate, as the gender‐related barriers to girls' education may be complex and multi‐faceted in many settings. However, given the design of many of the included multi‐component studies, it is difficult to draw conclusions about the extent to which some individual components affect schooling in a given setting. Designing multi‐component studies in such a way as to offer estimates of the contributions of each component (e.g., using a factorial design) would provide much‐needed clarity on which components are most essential.

We did not identify any studies meeting our inclusion criteria that test the effects of an intervention designed to address SRGBV on education outcomes for girls, nor did we find more than one study that evaluated an intervention with a sports or school‐based health or childcare component. Further, as our focus was on evidence from studies that employed methods to address endogeneity and better approximate the causal impact of a given intervention on schooling outcomes for girls, we did not include qualitative, mixed methods, and quantitative studies that did not use the methods outlined in our inclusion criteria. These studies provide context from which we constructed the framework for this review and provide important supporting information to explore the questions about how and why interventions might work laid out in the previous section.

### 6.3 Quality of the evidence

There was generally low risk of bias among the included studies, with 24% (*n *= 20) marked as having some concerns with risk of bias, and 5% (*n *= 4) having high risk of bias. The vast majority of experimental studies (36/41) had low risk of bias, though some common areas of concern were regarding deviations in assignment from intended interventions and selection of the reported result. Quasi‐experimental studies were far more likely to have higher risk of bias (19/41 had some concerns or high risk), particularly due to confounding and missing data. However, as noted above, this may be due to the phrasing of the questions in the ROBINS‐I tool, which was not designed for secondary analysis of exposures such as large‐scale policy changes, the analyses of which are more common in the social sciences.

### 6.4 Limitations and potential biases in the review process

This systematic review has a number of limitations and potential sources of bias, some of which are related to our approach, and other that are related to the nature of the evidence.

In terms of our approach, we note several limitations. In contrast to many previous reviews, we chose to define the inclusion criteria for our review, and organize our results, around barriers to education rather than specific interventions. We took this approach to try to illuminate the reasons that certain interventions, or groups of interventions, might be effective, rather than focusing on specific interventions (e.g., distributing bicycles, take‐home rations), which might be more context specific. However, this approach also presents new challenges. An agreed‐upon framework outlining potential gender‐related factors that may affect school participation and learning among girls in LMICs does not yet exist. Thus, researchers often did not specify particular barrier(s) programs aimed to address, nor did they use the same vocabulary or rationale when discussing barriers. Therefore, it is possible that we miscategorized some studies, and/or that our categorizations might differ from those that the authors themselves would use.

Similarly, many studies appear to address more than one barrier; thus, the same results are reported in multiple barrier categories. For example, Morrell et al. (2014) found significant effects of their intervention on attendance and literacy. Because the intervention appeared to address three barriers—gender insensitive school environment, lack of safe spaces and social connections, and insufficient academic support—those same results are listed in three barrier categories. The more categories a study addresses, the more it contributes to our overall findings. That is, undue influence, both in terms of effective interventions and noneffective interventions, is given to studies that appear to address multiple barriers. Finally, as noted, the more effects estimated and reported by a study, the greater the influence that study has on our aggregated findings.

Third, the contextual nature of these barriers, which are not of equal importance in every setting, inherently determines the potential for success of an intervention. In contexts where a gender‐related barrier such as child marriage is a primary cause of school‐leaving for girls, the intervention's potential for impact on education outcomes may be high and overcoming that barrier may also be more challenging. We note that almost half (36, or 44%) of the studies included in this review were conducted in four countries—India (14), Kenya (9), Uganda (7), Bangladesh (6). The extent to which these findings are transferable to other settings must be carefully considered in light of the gender‐related barriers that operate at national and subnational levels.

Another set of limitations of our analysis reflects the state of evaluation research in girls' education as it relates to school participation and learning. One key limitation is our inability to isolate the effectiveness of individual components for many multi‐component studies. For example, the BRIGHT school construction program included construction of girl‐friendly primary schools, incentives for children to attend school and mobilized community support for girls' schooling, and found significant effects on current enrolment, attainment and composite academic skills (Kazianga et al., 2012; Kazianga et al., 2019). The question is whether a particular component drove those effects or rather the combination of elements drove effects, which cannot be answered given the study design. Had the design been factorial, that is, included multiple appropriately powered study arms, each adding an additional relevant component, it would have been possible to compare the effect of those individual components with one another and the control. This would undeniably increase the costs of conducting a study, but it is feasible with sufficient resources. For example, Ashraf et al.'s (2018) design included three arms: the first arm included safe space groups with female mentors and training on negotiation and interpersonal communication; the second arm included safe space groups with female mentors; and the third arm was a control. The authors were able to determine whether providing a safe physical space for girls to meet is as effective in improving education outcomes as an intervention that adds training in negotiation skills to the safe space group.

A second limitation of this analysis is the number of different outcomes and measures reported, which made it difficult to compare study results. We identified 10 different outcome categories including, attendance, enrolment, re‐enrolment, attainment, completion, and academic skills. Furthermore, within categories, there is considerable variability in the measurement of specific outcomes. For example, for literacy, assessments measure: letter identification, familiar word identification, oral fluency (words and paragraphs), and/or reading comprehension. And, for some studies academic skills are assessed via a composite of literacy and numeracy rather than separate measures of literacy and numeracy. Some studies use standardized tools, for example, the Early Grade Math and Reading Assessments (EGMA and EGRA) developed by RTI International to assess foundational skills (2014, 2015) or the UWEZO learning assessment tool (Twaweza, n.d.); other studies use performance on national exams. Some studies use the actual scores; others use gains in scores. Some outcomes are measured dichotomously, others continuously. Some studies estimate separate effects by age or age group while others aggregate findings by age. This increases the difficulty of conducting meta‐analyses. However, instead we rated the strength of evidence of effects for interventions addressing each barrier based on the GRADE criteria, specifically effect direction and size, number of studies and participants, and certainty in the evidence. We recommend that future studies investigating the effect of interventions to improve girls' education include an agreed upon set of outcome measures in order to facilitate comparisons between studies.

A third limitation is that interventions and exposures differed markedly across studies, even within barriers. As mentioned previously, this also undermines our ability to conduct meta‐analyses. Given the importance of replication to draw definitive conclusions regarding the most effective interventions to improve girls' education outcomes, it would be helpful in the future if the same interventions were tested in multiple settings with similar evaluation designs.

### 6.5 Agreements and disagreements with other studies or reviews

As described previously, this review was designed to complement previous review—systematic or otherwise—that have assessed what works to improve education outcomes. Many of those reviews have focused broadly on what works overall, outlining differences by sex when available (Glewwe & Muralidharan, 2016; McEwan, 2015; Snilstveit et al., 2015), while others have focused more explicitly on differences by sex (Evans & Yuan, 2021), or girls' education and gender equality (Sperling & Winthrop, 2015; Unterhalter et al., 2014). Still other reviews have focused on specific types of interventions, such as cash transfers (Baird et al., 2013), which were excluded from our review, or MHM interventions (Sumpter & Torondel, 2013). To our knowledge, this is the first systematic review with a focus on the effects of interventions designed to address gender‐related barriers to education for girls. As such, there is some overlap with content of previous reviews, although our search was more recent, and our inclusion criteria (both in terms of topics and study design) are different from previous reviews.

We are unable to review all of the previous reviews on what works in education here due to the rapidly changing nature of the field, and challenges in comparing reviews directly. For example, in a review of reviews on what works to improve learning, Evans and Popova (2015) note that six reviews of the evidence had been published in the prior year alone. They find substantial variation in the conclusions drawn from each review, largely driven by the sample of research included. They note that across reviews, the three types of programs that are recommended somewhat consistently are: pedagogical interventions (including CAL) that tailor teaching to student skills; repeated teacher training interventions, often linked to another pedagogical intervention; and improving accountability through contracts or performance incentives in certain contexts. The authors echo an important point made by each review, which is that broad intervention categories (e.g., pedagogical interventions, computer interventions) are not necessarily wholly effective or ineffective, as the details of the specific interventions matter a great deal. They conclude that future reviews should, in part, separate out more specific interventions to provide concrete guidance to policymakers and practitioners. While this review of reviews does not focus on gender differences in effects, the broad findings about variations within and across categories of interventions are consistent with our findings.

We briefly review the findings of selected recent reviews in education, including those that focused on overall effects (of girls and boys combined), as well as those that focused more explicitly on gendered drivers or differences in outcomes. While we do not compare each one to our findings, we identify what we see as important areas of alignment or departure from previous reviews. The most comprehensive recent systematic review on what works in education was conducted by Sniltsveit et al. (2015). Evaluations were included if they used experimental or quasi‐experimental methods to examine program impacts on the following outcomes: enrolment, attendance, dropout, completion and learning. The review, which synthesized results from 216 programs in 52 LMICs, shared insights about what works at three levels: (1) children and households; (2) schools and teachers; and (3) systems. For children and households, the authors found that cash transfers are effective at improving participation, while merit‐based scholarships are most effective at improving learning outcomes. They also found that school feeding was a promising intervention both for increasing participation and test scores, but that the effects of providing information to children or parents, reducing user fees, and school‐based health programs are not clear due to lack of evidence. Overall our results largely align with these findings. For example, we conclude that interventions that address financial barriers (e.g., tuition and fees, inability to afford school materials) as well as those that address inadequate food may be effective or promising. We also find that despite some promising evidence, results are mixed on whether providing information to parents and students on returns to education leads to improvements in education outcomes for girls, and we found no studies that directly examined school‐based health programs.

For schools and teachers, Sniltsveit et al. (2015) found that programs using structured pedagogy to change the classroom environment had the largest and most consistent positive effects on learning of any interventions included in their review. They also found that remedial education, additional instructional time, and construction of new schools were promising for improving learning outcomes, but more research was needed; providing education‐related “hardware,” such as materials and technology, was often not sufficient to improve learning outcomes; and, despite limited evidence on teacher‐focused interventions, they found some evidence that teacher incentives have small effects on children's learning outcomes. Our results on programs including a strong training or remedial academic support component—a subset of those addressing lack of academic support—are consistent with these findings. As for provision of school materials, we found promising evidence that this approach may improve enrolment and attainment for girls. We also find evidence that the efforts to expand access to school, including school construction, are promising approaches to improving education outcomes for girls, especially enrolment, attainment and completion.

Glewwe and Muralidharan (2016) also published a review of the evidence for improving education outcomes in developing countries, through which they identified 118 high‐quality studies conducted from 1990 to 2014. They conclude that demand‐side interventions that increase the returns to schooling or reduce household costs, or increase students' returns to efforts, are effective at increasing time in school and learning outcomes but vary in cost‐effectiveness. They also argue that many expensive school inputs (e.g., school construction) are often not as effective at improving outcomes, while some (often less expensive) inputs are effective (e.g., bicycles). Our review includes both types of inputs, and finds some evidence in support of each, but we do not consider cost‐effectiveness. Glewwe and Muralidharan (2016) note the challenges in rigorously measuring the effects of many school inputs, including school infrastructure, teachers' education levels, and teacher training, and resulting lack of sufficient evidence on those topics. As was the case in our study, they identify a set of evaluations of what they describe as “large‐scale provision of resources,” including both interventions providing broad packages of school inputs, and large amounts of money that schools can use to buy the inputs of their choice. There is no overlap between the “large‐scale” interventions included in their review and those included in ours, but they find limited evidence of the effectiveness of these other categories of inputs on education outcomes overall. They describe three broad improvements to pedagogy that are likely to lead to improved performance in developing countries: (a) more effectively accounting for the variation in initial level of student preparation; (b) breaking the tight link between pedagogy and the textbook; and (c) focusing on education for all rather than just the elite. We did not identify enough studies that included these pedagogy‐related components to come to any solid conclusions on their effects on girls' school performance. They also find support for interventions that improve school governance—especially top‐down administrative monitoring—and teacher accountability. These types of interventions were largely lacking from the studies included in our review, except as a component of larger complex programs. They argue that the evidence points to several promising ways that spending on education can be done more efficiently.

Another recent review focused on school‐based interventions designed to improve learning (McEwan, 2015). The author identified 77 experiments with treatments broken down into three broad groups: instructional inputs (materials, computer/technology, grants, teacher training, class size/small‐group instruction/tracking), health inputs (food/beverage/nutrients, deworming drugs, malaria drugs, other), and incentives (information, performance incentives, contract/volunteer teachers, school management or supervision). The author finds that monetary grants and deworming treatments had no significant effect on average, and that a handful of other interventions (nutritional, information dissemination, school management) had small and somewhat inconsistent effects. He found larger effect sizes for the following interventions: computer/instructional technology, teacher training, smaller class sizes and learning groups or competency‐based grouping, contract or volunteer teachers, student and teacher performance incentives, and instructional materials. We find evidence that academic support/remedial education programs, most of which included a technology component, are effective approaches to improving learning outcomes for girls. This author also cautions that many of the interventions are implemented together, such as teacher training and other instructional inputs, making it difficult to estimate the precise contribution of each component on its own (McEwan, 2015).

While these reviews share important characteristics, including using rigorous inclusion criteria and reflecting on cost‐effectiveness of different interventions, none focused on gender differences in effects, or on interventions designed to address gender‐related barriers. Several other reviews have been conducted that have more directly integrated a gender perspective but have less often used clear criteria for inclusion of evaluations and/or ratings of the quality of evidence. A 2014 review focused on girls' education and gender equality identified 169 studies of interventions that improve girls' education and gender equality (Unterhalter et al., 2014). The theory of change guiding the review acknowledged, like our conceptual framework, that factors outside of the school environment affect these outcomes. Studies were included if they reported on an intervention related to the topic of the review and were published after 1991. In contrast to the other reviews discussed, the authors do not provide specific methodological criteria used for inclusion or ratings of study quality, although the latter was conducted. The authors divide interventions into three categories: those focusing on resources and infrastructure, changing institutions, and changing norms and including the most marginalized in education decision‐making. They hypothesize that, while each type of intervention can be effective on its own, impact will be greatest when these types of interventions are combined, a theory which in many ways is reflected in the interventions included in our review. With regard to resource and infrastructure interventions, they find that the effectiveness depends on careful targeting, and is enhanced when linked to processes associated with learning and teaching. This finding is consistent with the point made by Glewwe and Muralidharan (2016), that investments in infrastructure are most effective when linked with pedagogy. Unterhalter et al. (2014) note that these interventions are more likely to contribute to improvements in attendance, enrolment and attainment than empowerment or gender equality. They also note that, broadly, “in kind health interventions” can enhance enrolment and learning for boys and girls, though these should be in tandem with other interventions. They find no notable effects of direct school feeding and MHM interventions on test scores and attendance, respectively, which is consistent with our findings, although we did find support for the effects of school feeding on enrolment and attainment. On institutional change and policy, they underline the importance of having teachers who are adequately supported to enhance girls' schooling through education and training. They describe the importance of a “quality mix” that combines various approaches to enhancing quality, including concern with gender equality in teaching, attention to curriculum, learning materials and pedagogical practices, and attention to local context. They confirm that interventions designed to shift gender norms and enhance inclusion are under‐researched, and recommend further research on girls' clubs, faith communities, working with boys on gender equality, and strategies to include marginalized girls and women in decision‐making, among others. Though we find rigorous evaluations of interventions such as safe spaces provision, life skills and empowerment curricula, teacher training, and enhancing community support for girls' education, the multi‐component nature of the majority of these interventions makes it extremely difficult to conclude how these individually affect girls' schooling outcomes.

Another review focused on girls' education identified 138 studies to inform the core findings, which were identified as strong if the research was peer‐reviewed and/or met one of the following criteria: included a control group, measured outcomes before and after an intervention, isolated and controlled for variables, or was conducted over a sustained period of time (Sperling & Winthrop, 2015). Studies that did not meet these criteria were included but flagged as “promising,” while findings from anecdotes or other designs were identified as needing more research. As such, this review serves as a helpful catalogue of research on girls' education at the time, rather than a review of the most rigorous interventions and ratings of the quality of evidence. The authors share numerous insights that echo other reviews, including: the need to pair interventions focused on expanding school access with efforts to improve quality, as well as reducing the opportunity costs of schooling for girls; the potential benefit of school‐based health interventions for enrolment and learning; and the need to reduce time and distance to school for girls. They discuss the growing evidence on girl‐friendly schools, which they note often include a package of interventions aiming to improve quality and gender equality simultaneously. They also discuss growing evidence of the challenge posed by widespread SRGBV, and the potential impacts of efforts to provide gender sensitivity training to teachers and students and provide safe spaces to girls. Last, they discuss the evidence on improving the quality of schooling through teachers, including through improved pedagogy, increasing the number of female teachers, and training in gender sensitivity. While the authors do not weigh in on the relative quality of the evidence around many of these questions, with some exceptions, they raise important issues of relevance to our review. In many cases they include studies that did not meet our inclusion criteria, and therefore our findings may depart in some ways from theirs.

Our review examined three barriers that are financial, at least in part: inability to afford tuition and fees, inability to afford school materials, and lack of adequate food. We found evidence that interventions to address all three types of barriers are promising or effective. Given previous work on cash transfers, these interventions were excluded from our review, including scholarships or in‐kind transfers paid to households. However, it is worth mentioning that Baird et al. (2013) meta‐analysis on the effectiveness of conditional and unconditional cash transfers found that both sets of financial interventions may improve the odds of school enrolment and attendance overall, though the effects on test scores was minimal. Conditional interventions tended to have stronger effects on enrolment and attendance relative to unconditional interventions. As for gender, their results suggest that while both conditional and unconditional transfers were effective in increasing enrolment for boys and girls relative to control, conditional interventions may have had a marginally more significant impact on enrolment for girls relative to unconditional interventions. However, they note that there were fewer studies that disaggregated by sex, with implications for the interpretation of these results. The efficacy of financial incentives to improve education outcomes has been touched on in other reviews, and the results of Baird et al. (2013) systematic review and meta‐analysis are in line with the claims of those reviews.

Evans and Yuan (2021) sought to identify the programs most effective for improving girls' access and learning, comparing interventions aimed at girls with general interventions including both girls and boys. They conclude that interventions including boys and girls were as effective in improving access and learning as girl‐targeted interventions. As the authors acknowledge, targeting girls with an intervention is not the same as targeting gender‐related barriers to schooling and, as they also note, the finding that general interventions are as effective and have the added benefit of improving outcomes for boys does not mean that “we don't have to worry about gender in education.”

Generally, our results echo the findings of other reviews, though with some caveats. The challenges faced by previous reviews, most notably trying to ascertain the effects of individual components of large multicomponent programs, are challenges that we faced as well. Consistent with others' findings, we find evidence that interventions addressing financial barriers (tuition and fees, inadequate food, lack of school materials), inadequate school access, and lack of WASH facilities, especially toilets, may be promising or effective approaches to improving school enrolment and attainment for girls. However, many previous reviews indicate that programs that address pedagogy and skills‐based learning, such as teacher training and materials, academic support, and life skills or empowerment curricula, may help improve education outcomes. While we find evidence that remedial education or tutoring programs may be effective approaches to improving learning for girls, overall we find less focus on improving pedagogy in the gender‐focused literature. We also find that more research is needed to understand the circumstances in which life skills education and safe space programs might improve education outcomes for girls.

## 7 AUTHORS' CONCLUSIONS

### 7.1 Implications for research

To our knowledge, this is the first systematic review that has assessed the effects of interventions designed to address gender‐related barriers to education for girls. As others have noted, much of the existing evidence on what works to improve education outcomes in LMICs is not disaggregated by sex (Evans & Yuan, 2021). Beyond disaggregation of results, previous rigorous reviews on education interventions largely did not focus on interventions designed to address gender‐related barriers, such as construction of “girl‐friendly” schools or addressing SRGBV, unless they were explicitly focused on girls. Through this review we have attempted to bring together two often divergent areas of research. In some areas we find that results are consistent with previous evidence, for example around cost of schooling and expanding access to school. In other areas we find that insufficient evidence exists on whether some widespread intervention approaches, including MHM, are likely to improve education outcomes for girls, and in which settings.

We have identified several core implications for research and practice based on this review. First, we find evidence gaps for the girls' education impact of interventions designed to address the following areas due to lack of directly relevant research: lack of support for girls' education, child marriage and adolescent pregnancy, SRGBV, lack of safe spaces and social connections, lack of teaching materials and supplies, inadequate sports programs for girls, inadequate health and childcare services, and inadequate MHM; or because of inconsistent effects: lack of information on returns to education/alternative roles for women, gender‐insensitive school environment, inadequate life skills, and poor policy/legal environment. There were also no studies identified that looked at important subsets of these barriers, specifically, no evaluations of the impact on education outcomes of comprehensive sexuality education, nor of textbooks and other learning materials that are free of gender biases and stereotypes.

We find evidence of effectiveness of interventions addressing three barriers (inability to afford tuition and fees, lack of adequate food, and insufficient academic support), and promising results for interventions that address three of the barriers (lack of water and sanitation, inadequate school access, inability to afford school materials). That said, not all of these barriers should necessarily privilege education outcomes. For example, for programs that aim to reduce child marriage, it may or may not be the case that what works best for education outcomes is the same as what works best to delay marriage. This is a testable question, but one that also includes weighing inequality and vulnerability.

Second, in our search, we identified studies that, though rigorous, did not disaggregate their results by sex. One essential step toward building the evidence base in these areas is to power studies with sufficient sample sizes to disaggregate results by sex, and report results for males and females even if there are no significant differences.

Third, many of the interventions evaluated by studies included in our review contained numerous components, and most evaluations were not designed to disentangle the effects of those components. While it is useful to have examples of large‐scale multi‐component programs that have effectively improved education outcomes for girls, information on which components are most effective, and for whom—and, equally important, which components do not contribute to improved outcomes—might be more practically useful for those seeking to adapt successful interventions across settings and/or at scale. Studies that can tease out whether and what specific combinations of components are a recipe for success are similarly lacking.

Related to this point, the fourth implication of our research is that many interventions that appear to be targeting a similar barrier (e.g., inadequate life skills education) even with a small number of program components, do so in vastly different ways, ranging from different duration of interventions, frequency of meetings, curricular content, role of mentors/facilitators, etc. Without shared definitions of core components of these approaches, even high‐quality studies will be difficult to compare, and results would be difficult to apply across settings. Similarly, it may be beneficial for future studies to explore the pathways between interventions and their effects on education outcomes, as it is difficult to understand how and why changes do or do not take place without explicit recognition of the barriers a study aims to address and an explicit theory of change describing how changes are likely to occur.

## ROLES AND RESPONSIBILITIES

- Content: Stephanie Psaki, Nicole Haberland, Barbara Mensch, and Erica Chuang.
- Systematic review methods: Barbara Mensch, Stephanie Psaki, Nicole Haberland, and Erica Chuang.
- Statistical analysis: Erica Chuang and Lauren Woyczynski.
- Information retrieval: Erica Chuang, Lauren Woyczynski, Anne Smith, Grace Sheehy, Aditi Patrikar, Jeanette Shekelle, Fiona Gambanga, Nura Anwar, Rachel Passmore, Lili Warren, and Isabel Odean.
- Advisory group members: David Evans (World Bank), Matthew Jukes (RTI International), Cynthia Lloyd (Independent Consultant), Patrick McEwan (Wellesley College), and Birte Snilstveit (3ie).
- Other independent consultants and advisors: Katherine Willson (Independent Consultant).

## SOURCES OF SUPPORT

This project is funded by Echidna Giving.

## DECLARATIONS OF INTEREST

The review team has no known conflicts of interest.

## PLANS FOR UPDATING THE REVIEW

The authors plan to update the review within 3 years.

## Supporting information

Supporting information.

Supporting information.

Supporting information.

Supporting information.

Supplementary Information

## Footnotes

^1^
Nutritional supplementation interventions at school are included as a separate barrier due to their effects on the household spending, in addition to child health.

^2^
Policies that reduce or eliminate school fees are featured under the inability to afford tuition and fees barrier.

^3^
Cash transfer interventions targeting individuals and households were not included in this review because there have been several recent systematic reviews that have analyzed these programs (e.g., Baird et al., 2013; Snilstveit et al., 2015).

^4^
As stated above, although a common intervention approach, poverty reduction interventions targeting girls will not be included in this review because there have been several recent systematic reviews that have analyzed these programs (e.g., Baird et al., 2013; Snilstveit et al., 2015).

^5^
Endogeneity arises when an explanatory variable is correlated with the error term either because it is unobserved/unmeasured, or because there is measurement error, or because there is simultaneity, that is, it is jointly determined with the dependent variable (Wooldridge, 2012).

^6^
Studies that analyze the effects of a RCT but only report data collected after the intervention was implemented were eligible only if they satisfied one of the following criteria: (1) studies do not suffer from issues of statistical power as given by sample size calculations reported in the study, or (2) they report a total sample size of over 10,000, or have more than 1000 observations per cluster in each arm on average. These criteria are roughly drawn from Muralidharan (2017).

^7^
The list is drawn from Shadish et al. (2002).

^8^
For two studies (Aber et al., 2017; Mbiti et al., 2019), we converted the reported gender‐differential results for boys to those of girls by multiplying the estimated effects by −1.

^9^
Note that the arms that include the in‐kind incentive to delay marriage were not included among our independent results, and that this description is provided purely for context.

^10^
Eble and Hu 2019 focused on girls who perceived themselves to have lower ability in math.

^11^
It should be noted that the intervention was relatively short‐term, comprising six after school sessions over two weeks, and one additional “booster” session one to 2 months later.

^12^
We only present results from the safe spaces arm in our tables. The study also included a safe spaces arm combined with a cash transfer that found a small effect (*p* < .10) on school enrolment, though neither arm was effective in reducing the incidence of sexual violence, which was their primary objective.

^13^
Notably, the kit of clothes and school bag for girls provided no additional benefits for any of these outcomes. That is, the impact of the learning materials and supplementary teaching intervention plus clothing kit for girls was not statistically different from the learning materials and supplementary teaching intervention alone. However, this additional clothing arm was not compared to the control group, and thus was not included in our analysis.

^14^
It should be noted that Beg et al. reported the results of standardized tests that combine multiple subject matters beyond literacy and numeracy, but those results were excluded from our review based on our list of prespecified outcomes.

^15^
It is notable, however, that this program led to increased HSV‐2 incidence for girls, and other indicators that participation in secondary school may have led to peer pressure for a higher standard of living, and changes in attitudes toward sexual behaviour that led to increased risk‐taking (Cho et al., 2019).

^16^
When the authors ran models excluding transfer‐related absences, the estimated effects of the intervention on absenteeism were no longer statistically significant, though they point out that this sample is biased by the fact that transfer students were more likely to be older girls (Benshaul‐Tolonen et al., 2019).

^17^
Note that the results from converting estimated effects to partial correlation coefficients appear to indicate a significant increase in school dropout in both intervention arms, but this is a result of error introduced in the conversion process, and is not consistent with the null results reported by the authors.

^18^
Note that free primary and secondary education policies fall under the inability to afford tuition and fees (Barrier 16) due to the financial nature of those types of policies.
