# Supplementary material for: Policies and interventions to remove gender‐related barriers to girls' school participation and learning in low‐ and middle‐income countries: A systematic review of the evidence
Source: Campbell Syst Rev. 2022 Jan 19;18(1):e1207. doi: 10.1002/cl2.1207 (PMC8770660; doi:10.1002/cl2.1207)
Supplement: Supplementary file 1 — Supporting information. [file CL2-18-e1207-s002.docx]

# Appendix

## Section A. Search Strategy and Search Terms

1. Search Strategy

We used the following search strategy to search ERIC and adapted it for the databases listed in the Methods section. Searches limited to 2000-2019 publication dates. The search terms below are grouped by:

- Geographic (regions and countries) Set (S4)
- Year Published Set (S5)
- Gender Set (S7)
- Education Set (S9)
- Study Set (S13)
- Intervention Set (S19)

2. Search Terms

| **S#** | **SET NAME** | **QUERY: BOOLEAN/PHRASE IN ERIC ON EBSCOHOST MONDAY FEBRUARY 11, 2019** |
| --- | --- | --- |
| S1 | REGION | Africa OR Asia OR Caribbean OR 'Central America' OR 'Latin America' OR 'Middle East' OR 'South America' OR 'West Indies' |
| S2 | COUNTRIES | Afghanistan OR Albania OR Algeria OR Angola OR Argentina OR Armenia OR Azerbaijan OR Bangladesh OR Benin OR Belarus OR Belize OR Bhutan OR Bolivia OR Bosnia OR Herzegovina OR Botswana OR Brasil OR Brazil OR 'British Virgin Islands' OR Bulgaria OR 'Burkina Faso' OR Burundi OR 'Cabo Verde' OR 'Cape Verde' OR Cambodia OR Cameroon OR 'Central African Republic' OR Chad OR Chile OR China OR Colombia OR Comoros OR Congo OR 'Costa Rica' OR 'Cote d'Ivoire' OR 'Ivory Coast' OR Croatia OR Cuba OR 'Czech Republic' OR 'Slovakia' OR Djibouti OR Dominica OR 'Dominican Republic' OR 'DPR Korea' OR 'East Timor' OR 'Timor Leste' OR Ecuador OR Egypt OR 'El Salvador' OR Eritrea OR Estonia OR Ethiopia OR Fiji OR Gabon OR Gambia OR Gaza OR (Georgia W5 republic) OR Ghana OR Grenada OR Guatemala OR Guinea OR 'Guinea Bissau' OR Guyana OR Haiti OR Honduras OR Hungary OR India OR Indonesia OR Iran OR Iraq OR Jamaica OR Jordan OR Kazakhstan OR Kenya OR Kiribati OR Kosovo OR 'Kyrgyz Republic' OR Kyrgyzstan OR 'Lao PDR' OR Laos OR Latvia OR Lebanon OR Lesotho OR Liberia OR Libya OR Lithuania OR Macedonia OR Madagascar OR Malaysia OR Malawi OR Maldives OR Mali OR 'Marshall Islands' OR Mauritania OR Mauritius OR Mexico OR Micronesia OR Moldova OR Mongolia OR Montenegro OR Morocco OR Mozambique OR Myanmar OR Burma OR Namibia OR Nauru OR Nepal OR Nicaragua OR Niger OR Nigeria OR 'Northern Mariana Islands' OR Oman OR Pakistan OR Palau OR Panama OR Paraguay OR Peru OR Philippines OR Poland OR Romania OR 'Russian Federation' OR Russia OR Rwanda OR 'Saint Kitts' OR 'St Kitts' OR Nevis OR 'St Lucia' OR 'Saint Lucia' OR 'Saint Martin' OR 'St Martin' OR 'St Vincent' OR 'Saint Vincent' OR Grenadines OR Samoa OR 'Sao Tome' OR Principe OR Senegal OR Serbia OR Seychelles OR 'Sierra Leone' OR 'Soloman Islands' OR Somalia OR 'South Africa' OR 'Sri Lanka' OR Sudan OR Suriname OR Swaziland OR 'Syrian Arab Republic' OR Syria OR Tajikistan OR Tanzania OR Thailand OR Togo OR Tonga OR Trinidad OR Tobago OR Tunisia OR Turkey OR Turkmenistan OR (Turks W1 Caicos) OR Tuvalu OR Uganda OR Ukraine OR Uruguay OR Uzbekistan OR Vanuatu OR Venezuela OR Vietnam OR 'Viet Nam' OR 'West Bank' OR Yemen OR Zambia OR Zimbabwe |
| S3 | LMIC TEXTWORDS | (developing W1 nation) OR (developing W1 nations) OR (developing W1 countr*) OR (developing W1 econom*) OR (developing W1 world) OR (less* W1 developed W1 nation) OR (less* W1 developed W1 nations) OR (less* W1 developed W1 countr*) OR (less* W1 developed W1 econom*) OR (less* W1 developed W1 world) OR (under* W1 developed W1 nation) OR (under* W1 developed W1 nations) OR (under* W1 developed W1 countr*) OR (under* W1 developed W1 econom*) OR (under* W1 developed W1 world) OR (underdeveloped W1 nation) OR (underdeveloped W1 nations) OR (underdeveloped W1 countr*) OR (underdeveloped W1 econom*) OR (underdeveloped W1 world) OR (low* W2 income W1 nation) OR (low* W2 income W1 nations) OR (low* W2 income W1 countr*) OR (low* W2 income W1 econom*) OR (middle W1 income W1 nation) OR (middle W1 income W1 nations) OR (middle W1 income W1 countr*) OR (middle W1 income W1 econom*) OR (underserved W1 nation) OR (underserved W1 nations) OR (underserved W1 countr*) OR (deprived W1 nation) OR (deprived W1 nations) OR (deprived W1 countr*) OR (poor W1 nation) OR (poor W1 nations) OR (poor W1 countr*) OR (third W1 world) OR (transitional W1 nation) OR (transitional W1 nations) OR (transitional W1 countr*) OR (transitional W1 econom*) |
| S4 | GEOGRAPHIC SET | S1 or S2 OR S3 |
| S5 | YEAR PUBLISHED | YR 2000-2019 |
| S6 | GEO/YR SET | S4 AND S5 |
| S7 | GENDER SET | gender OR woman OR women OR female OR females OR girl* OR (sex W1 role*) OR (sex W1 difference*) OR DE sex differences OR DE sex role |
| S8 | GEN/GEO/YR SET | S6 AND S7 |
| S9 | EDUCATION SET | SU education* OR school* OR learn* |
| S10 | ED/GEN/GEO/YR SET | S8 AND S9 |
| S11 | STUDY DESIGN | ('random* control* trial*') OR (control W1 endogeneity) OR (regression W1 discontin*) OR (regression W1 model*) OR ('instrumental variable* analys*') OR ('interrupted time series') OR (pre W1 test*) OR (post W1 test*) OR (pretest*) OR (posttest*) OR ('match* comparison group*') OR (matching N2 procedure*) OR (comparison W1 group*) OR (control W1 group*) OR (quasi W1 experiment*) OR (quasiexperiment*) OR (mixed W1 method*) OR (cross W1 random*) OR (least W1 square* W1 model*) OR (experiment*) OR SU regression OR DE randomized controlled trials OR DE pretests posttests OR DE quasiexperimental design OR DE educational experiments OR DE educational research OR DE mixed methods research OR DE program effectiveness OR DE program evaluation |
| S12 | STUDY RESULT | (evidence N4 intervention*) OR (evaluat* N4 intervention*) OR (impact* N4 intervention*) OR (effect* N4 intervention*) OR (result* N4 intervention*) OR (outcome* W1 measure*) OR (causal) OR (case W1 stud*) OR (best W1 practice*) OR (what W1 workS) OR (program W2 result*) OR (program W1 evaluation*) OR (program W1 effect*) |
| S13 | STUDY SET | S11 OR S12 |
| S14 | STUDY/ED/GEN/GEO/YR SET | S10 AND S13 |
| S15 | INTERVENTION DESCRIPTORS | DE academic achievement OR DE access to education OR DE after school programs OR DE critical thinking OR DE curriculum OR DE daily living skills OR DE educational attainment OR DE empowerment OR DE emotional development OR DE fees OR DE HIV OR DE AIDS OR DE marriage OR DE married students OR DE pregnancy OR DE pregnant students OR DE sanitary facilities OR DE sanitation OR DE school safety OR DE school security OR DE school uniforms OR DE sexually transmitted infections OR DE student costs OR DE teacher education OR DE textbooks OR DE violence OR DE women administrators OR DE women faculty |
| S16 | INTERVENTIONS TEXTWORDS | (academic W1 achieve*) OR (academic W1 engagement) OR (grade W1 attain*) OR (grade* N2 repetition) OR (grade* N2 progress*) OR (education* W1 attain*) OR (enroll* N2 status) OR (school* N2 access) OR (school * W1 participation) OR (school* W1 supplies) OR (school* N3 fee*) OR (school* N3 dropout*) OR (school* N2 facilit*) OR (school* N2 infrastructur*) OR (school* N4 sanita*) OR (school* N2 quality) OR (school* N2 complet*) OR (school* N2 enroll*) OR (school* N2 continu*) OR (school* N2 attend*) OR (school* N2 safety) OR (school* W1 security) OR (school* N4 uniform*) OR (school* N4 (toilet* OR latrine*)) OR (village W2 school*) OR (distance W2 school*) OR (barrier* N3 retention) OR (gender n4 violence) OR (gender wN2 sensitiv*) OR (gender W1 norm*) OR (gender N2 discriminat*) OR (gender N3 attitude*) OR (early W1 childbearing) OR (early W1 marr*) OR (child W1 marr*) OR (adolescen* N2 pregnan*) OR (adolescen* N2 childbearing) OR (adolescen* W1 mother*) OR (parent* N3 attitude*) OR (opportunity W1 cost*) OR (direct W1 cost*) OR (legal W1 framework*) OR (political N2 support) OR (health W2 issue*) OR (HIV N3 prevent*) OR (STI OR STD) OR (cash W1 transfer*) OR (access W2 service*) OR (transition N2 work*) OR (teacher N3 bias*) OR (teacher N3 attitude*) OR (teacher N3 train*) OR (teacher W1 education) OR (female* N3 administrator*) OR (female* N3 teach*) OR (years W2 school*) OR (gender N2 equality) OR (earning N2 capacity) OR (student* N4 pregnan*) OR (curricul*) OR (women W1 faculty) OR (women W1 administrat*) OR (student W1 cost*) OR (after W1 school W1 program*) OR (married W1 student*) OR (textbook*) OR (menstrual) OR (student* N3 re-enrolment) OR (test score*) OR (exam taking) OR (sexual W1 reproductive W1 health) |
| S17 | SKILL INTERVENTION TERMS | (critical W1 thinking) OR (emotion al W1 learning) OR (daily W1 living) OR (emotional W1 development) OR (self W1 efficacy) OR (mental W1 health) OR (knowledge W1 rights) OR (civic W1 engagement) OR (financial W1 literacy) OR (self W1 aware*) OR (skill* N2 acqui*) OR (cognitive W1 ability) |
| S18 | SKILLS INTERVENTION GROUPED | (life OR negotiat* OR relationship* OR decisionmaking OR agency OR empower* OR abuse OR ambition OR coercion OR communicat* OR autonomy OR literacy OR numeracy OR advocacy OR resilience OR harassment OR aspiration OR sexual*) N5 skill* |
| S19 | INTERVENTION SET | S15 OR S16 OR S17 OR S18 |
| S20 | INTERV/STUDY/ED/GEN/GEO/YR SET | S14 AND S19 |

## Section B. Assessment of Risk of Bias

The following risk of bias tools were adapted from RoB 2 (Higgins et al., 2018) for randomized studies, and ROBINS-I (Sterne et al., 2016) and Psaki et al. (2019) for non-randomized studies. The following are the values for response options:

- Y=“Yes”
- PY=“Possibly Yes”
- PN=“Possibly No”
- N=“No”
- NI=“No Information”
- NA=“Not Applicable”

Values in green indicate response options for which there may be low risk of bias. Values in red indicate response options for which there may be high risk of bias. We follow the guidelines for RoB 2, ROBINS-I and Psaki et al. (2019) as closely as possible to judge for risk of bias.

**1) Randomized Studies**

| **Bias Domain** | **Signalling questions** | **Response Options** |
| --- | --- | --- |
| **Bias from randomization process** | 1.1 Was the allocation sequence random? | Y / PY / PN / N / NI |
|  | 1.2 Was the allocation sequence concealed until participants were enrolled and assigned to interventions? | Y / PY / PN / N / NI |
|  | 1.3 Did baseline differences between intervention groups suggest a problem with the randomization process? | Y / PY / PN / N / NI |
|  | **Risk-of-bias judgement** | Low / High / Some concerns |
| **Bias from deviations in assignment from intended interventions** | 2.1. Were participants aware of their assigned intervention during the trial? | Y / PY / PN / N / NI |
|  | 2.2. Were carers and people delivering the interventions aware of participants' assigned intervention during the trial? | Y / PY / PN / N / NI |
|  | 2.3. If Y/PY/NI to 2.1 or 2.2, Were there deviations from the intended intervention that arose because of the experimental context? | NA / Y / PY / PN / N / NI |
|  | 2.4. If Y/PY to 2.3, Were these deviations from intended intervention balanced between groups? | NA / Y / PY / PN / N / NI |
|  | 2.5 If N/PN/NI to 2.4, were these deviations likely to have affected the outcome? | NA / Y / PY / PN / N / NI |
|  | 2.6 Was an appropriate analysis used to estimate the effect of assignment to intervention? | Y / PY / PN / N / NI |
|  | 2.7 If N/PN/NI to 2.6, was there potential for a substantial impact (on the result) of the failure to analyze participants in the group to which they were randomized? | NA / Y / PY / PN / N / NI |
|  | **Risk-of-bias judgement** | Low / High / Some concerns |
| **Bias from missing outcome data** | 3.1 Were data for this outcome available for all, or nearly all, participants randomized? | Y / PY / PN / N / NI |
|  | 3.2 If N/PN/NI to 3.1, is there evidence that result was not biased by missing outcome data? | NA / Y / PY / PN / N |
|  | 3.3 If N/PN to 3.2, could missingness in the outcome depend on its true value? | NA / Y / PY / PN / N / NI |
|  | 3.4 If Y/PY/NI to 3.3, do the proportions of missing outcome data differ between intervention groups? | NA / Y / PY / PN / N / NI |
|  | 3.5 If Y/PY/NI to 3.3, is it likely that missingness in the outcome depended on its true value? | NA / Y / PY / PN / N / NI |
|  | **Risk-of-bias judgement** | Low / High / Some concerns |
| **Bias due to measurement of outcome** | 4.1 Was the method of measuring the outcome inappropriate? | Y / PY / PN / N / NI |
|  | 4.2 Could measurement or ascertainment of the outcome have differed between intervention groups? | Y / PY / PN / N / NI |
|  | 4.3 If N/PN/NI to 4.1 and 4.2, were outcome assessors aware of the intervention received by study participants? | Y / PY / PN / N / NI |
|  | 4.4 If Y/PY/NI to 4.3, could assessment of the outcome have been influenced by knowledge of intervention received? | NA / Y / PY / PN / N / NI |
|  | 4.5 If Y/PY/NI to 4.4, is it likely that assessment of the outcome was influenced by knowledge of intervention received? | NA / Y / PY / PN / N / NI |
|  | **Risk-of-bias judgement** | Low / High / Some concerns |
| **Bias due to selection of reported result** | 5.1 Was the trial analyzed in accordance with a prespecified plan that was finalized before unblinded outcome data were available for analysis? | Y / PY / PN / N / NI |
|  | Is the numerical result being assessed likely to have been selected, on the basis of the results, from... |  |
|  | 5.2. ... multiple outcome measurements (e.g. scales, definitions, time points) within the outcome domain? | Y / PY / PN / N / NI |
|  | 5.3 ... multiple analyses of the data? | Y / PY / PN / N / NI |
|  | **Risk-of-bias judgement** | Low / High / Some concerns |
| **Overall risk of bias** | **Risk-of-bias judgement** | Low / High / Some concerns |

**2) Non-randomized Studies**

| **Bias Domain** | **Signalling questions** | **Response Options** |
| --- | --- | --- |
| **Bias due to confounding** | 1.1 Is there potential for confounding of the effect of the intervention in this study? | Y / PY / PN / N / NI |
|  | If Y/PY to 1.1: Assess time-varying confounding: |  |
|  | 1.2 Was the analysis based on splitting participants’ follow-up time according to intervention received?  If N/PN, answer questions relating to baseline confounding (1.4 to 1.6)  If Y/PY, proceed to question 1.3. | NA / Y / PY / PN / N / NI |
|  | 1.3 Were intervention discontinuations or switches likely to be related to factors that are prognostic for the outcome?  If N/PN, answer questions relating to baseline confounding (1.4 to 1.6)  If Y/PY, answer questions relating to both baseline and time-varying confounding (1.7 and 1.8) | NA / Y / PY / PN / N / NI |
|  | **Related to baseline confounding only**: |  |
|  | 1.4 Did the authors use an appropriate analysis method that controlled for all the important confounding domains? | NA / Y / PY / PN / N / NI |
|  | 1.5 If Y/PY to 1.4, were confounding domains that were controlled for measured validly and reliably for measured variables available in this study? | NA / Y / PY / PN / N / NI |
|  | 1.6 Did the authors control for any post-intervention variables that could have been affected by the intervention? | NA / Y / PY / PN / N / NI |
|  | **Related to time-varying confounding**: |  |
|  | 1.7 Did the authors use an appropriate analysis method that adjusted for time-varying confounding? | NA / Y / PY / PN / N / NI |
|  | 1.8 If Y/PY to 1.7, were confounding domains that were adjusted for measured validly and reliably by the variables available in this study? | NA / Y / PY / PN / N / NI |
|  | **Risk-of-bias judgement** | Low / High / Some concerns |
| **Bias in selection of participants into the study** | 2.1 Was selection of participants into the study (or into the analysis) based on participant characteristics observed after the start of the intervention? | Y / PY / PN / N / NI |
|  | If Y/PY to 2.1: |  |
|  | 2.2 Were the post-intervention variables that influenced selection likely to be associated with the intervention? | NA / Y / PY / PN / N / NI |
|  | 2.3 Were the post-interventions that influenced selection likely to be influenced by the outcome or a cause of the outcome? | NA / Y / PY / PN / N / NI |
|  | 2.4 Do start of follow-up and start of intervention coincide for most participants? | Y / PY / PN / N / NI |
|  | 2.5 If Y/PY to 2.2 and 2.3, or N/PN to 2.4, were adjustment techniques used likely to correct for the presence of selection biases? | NA / Y / PY / PN / N / NI |
|  | **Risk-of-bias judgement** | Low / High / Some concerns |
| **Bias in classification of interventions** | 3.1 Were intervention groups clearly defined? | Y / PY / PN / N / NI |
|  | 3.2 Was the information used to define intervention groups recorded at the start of the intervention? | Y / PY / PN / N / NI |
|  | 3.3 Could classification of intervention status have been affected by knowledge of the outcome or risk of the outcome? | Y / PY / PN / N / NI |
|  | **Risk-of-bias judgement** | Low / High / Some concerns |
| **Bias due to deviations from intended interventions** | 4.1 Were the deviations from the intended intervention beyond what would be expected in usual practice? | Y / PY / PN / N / NI |
|  | 4.2 If Y/PY to 4.1, were these deviations from intended intervention unbalanced between groups *and* likely to have affected the outcome? | NA / Y / PY / PN / N / NI |
|  | **Risk-of-bias judgement** | Low / High / Some concerns |
| **Bias due to missing data** | 5.1 Were outcome data available for all, or nearly all, participants? | Y / PY / PN / N / NI |
|  | 5.2 Were participants excluded due to missing data on intervention status? | Y / PY / PN / N / NI |
|  | 5.3 Were participants excluded due to missing data on other variables needed for the analysis? | Y / PY / PN / N / NI |
|  | If PN/N to 5.1, or Y/PY to 5.2 or 5.3: |  |
|  | 5.4 Are the proportion of participants and reasons for missing data similar across interventions? | NA / Y / PY / PN / N / NI |
|  | 5.5 Is there evidence that results were robust to the presence of missing data? | NA / Y / PY / PN / N / NI |
|  | **Risk-of-bias judgement** | Low / High / Some concerns |
| **Bias in measurement of outcomes** | 6.1 Could the outcome measure have been influenced by knowledge of the intervention received? | Y / PY / PN / N / NI |
|  | 6.2 Were outcome assessors aware of the intervention received by study participants? | Y / PY / PN / N / NI |
|  | 6.3 Were the methods of outcome assessment comparable across intervention groups? | Y / PY / PN / N / NI |
|  | 6.4 Were any systematic errors in measurement of the outcome related to intervention received? | Y / PY / PN / N / NI |
|  | **Risk-of-bias judgement** | Low / High / Some concerns |
| **Bias in selection of the reported result** | Is the reported effect estimate likely to be selected on the basis of the results, from… |  |
|  | 7.1 …multiple outcome *measurements* within the outcome domain? | Y / PY / PN / N / NI |
|  | 7.2 …multiple *analyses* of the intervention-outcome relationship? | Y / PY / PN / N / NI |
|  | 7.3 …different subgroups? | Y / PY / PN / N / NI |
|  | **Risk-of-bias judgement** | Low / High / Some concerns |
| **Methods-specific criteria** | **Natural experiments** (Craig et al., 2012): | Y / N |
|  | 8.1.1 The authors note that the analysis was of a natural experiment or an exogenous change/event. | Y / PY / PN / N / NI |
|  | 8.1.2 The context in which the natural experiment occurred was described. | Y / PY / PN / N / NI |
|  | 8.1.3 The intervention and assignment process were described. | Y / PY / PN / N / NI |
|  | 8.1.4 The methods used to estimate impact were explicitly stated. | Y / PY / PN / N / NI |
|  | 8.1.5 Quantitative procedures to reduce the risk of bias were implemented and qualitative and/or theoretical justification was provided for use of aforementioned procedures. | Y / PY / PN / N / NI |
|  | **Regression discontinuity design:** | Y / N |
|  | 8.2 Authors gave justification for the distance from the cut-off point between treatment and control or authors weighted the matches to their distance to the cut-off point. | Y / PY / PN / N / NI |
|  | **Matching methods** (Stuart, 2010): | Y / N |
|  | 8.3.1 Covariates used were explicitly listed and were all not associated with treatment assignment and outcomes of interest. | Y / PY / PN / N / NI |
|  | 8.3.2 Authors performed and reported the results of a Rosenbaum test (or equivalent) for hidden bias. | Y / PY / PN / N / NI |
|  | 8.3.3 More than 90% of matches made. | Y / PY / PN / N / NI |
|  | If the study uses Mahalanobis: |  |
|  | 8.3.4 Fewer than 8 covariates were utilized. | NA / Y / PY / PN / N / NI |
|  | 8.3.5 Authors state that all covariates are normally distributed. | NA / Y / PY / PN / N / NI |
|  | If the study uses propensity score matching: |  |
|  | 8.3.6 Propensity score estimation model has been validated (e.g. logistic regression, boosted CART, generalized boosted models). | NA / Y / PY / PN / N / NI |
|  | 8.3.7 If linear propensity score matching, used a caliper of 0.25 standard deviations of the linear propensity score or justification is given for a larger caliper. | NA / Y / PY / PN / N / NI |
|  | If the study uses nearest-neighbor matching: |  |
|  | 8.3.8 If caliper matching, 90% of matches are within one standard deviation of the mean of covariate, or justification is given for a larger caliper . | NA / Y / PY / PN / N / NI |
|  | 8.3.9 If with replacement, causal inference issues are noted and addressed in the analysis (e.g. using frequency weights). | NA / Y / PY / PN / N / NI |
|  | 8.3.10 If with replacement, the number of times each control is matched is noted and justification is given if there are a large number of matches for each control. | NA / Y / PY / PN / N / NI |
|  | **Heckman correction and instrumental variables:** | Y / N |
|  | 8.4.1 The instrumenting equation is significant at F≥10 or the author reports and assesses whether the R-squared of the instrumenting equation is adequate for appropriate identification. | Y / PY / PN / N / NI |
|  | 8.4.2 All instruments/corrections are reported and significant p≤0.05. | Y / PY / PN / N / NI |
|  | 8.4.3 The authors qualitatively assess the exogeneity of the instrument/identifier and provide a theoretical justification for it. | Y / PY / PN / N / NI |
|  | 8.4.4 A Hausman test for exogeneity (or equivalent) was performed and reported where the authors cannot reject the null at 95% confidence. | Y / PY / PN / N / NI |
|  | **Maximum likelihood models or OLS:** | Y / N |
|  | 8.5 A Hausman test for exogeneity (or equivalent) was performed and reported where the authors cannot reject the null at 95% confidence. | Y / PY / PN / N / NI |
|  | **Controlled pre/post or difference-in-differences:** | Y / N |
|  | 8.6 The authors used a fixed effects or difference-in-differences multivariate regression. | Y / PY / PN / N / NI |
|  | **Interrupted time series** (Bernal, Cummins, and Gasparrini, 2017): |  |
|  | 8.7.1 The period of implementation of the policy or intervention was well defined, or the authors specified a period of graduate roll-out. | Y / PY / PN / N / NI |
|  | 8.7.2 The authors attempt to control for seasonality through methods such as time stratified models, periodic functions (i.e. Fourier terms), and splines or they provided justification for why seasonality was not an issue. | Y / PY / PN / N / NI |
|  | 8.7.3 The data analyzed contains more than one time point before and after the period of implementation of policy or intervention. | Y / PY / PN / N / NI |
|  | 8.7.4 Autocorrelation was assessed by analyzing a plot of the residuals and the partial autocorrelation function or deviance residuals over time, using a 95% CI. | Y / PY / PN / N / NI |
|  | 8.7.5 If data are normally distributed, a Breusch-Godfrey test was conducted where the authors could not reject the null at 95% confidence. | NA / Y / PY / PN / N / NI |
|  | 8.7.6 If residual autocorrelation was assessed to be present, a model such as Prais regression or ARIMA model was used in order to adjust for autocorrelation. | NA / Y / PY / PN / N / NI |
|  | 8.7.7 If a Poisson distribution was assumed, over-dispersion was controlled for using a scaling adjustment though a Pearson chi-squared test (utilizing the residual degrees of freedom) assessed at 95% confidence. | NA / Y / PY / PN / N / NI |
|  | **Risk-of-bias judgement** | Low / High / Some concerns |
| **Overall risk of bias** | **Risk-of-bias judgement** | Low / High / Some concerns |

## Section C. Deviations from Protocol

The following are discrepancies between the protocol and the review. These differences were conscious decisions made by the authors over the course of conducting selection of studies and analysis that were deemed necessary for ease of interpretation, and are organized by the overarching section of the review.

- Methods
  - Criteria for considering studies for this review
    - Included the following barriers based on the studies identified in the search:
      - Lack of teaching materials and supplies
      - Lack of safe spaces and social connections
      - Lack of information on returns to education/alternative roles for women
      - Child marriage and adolescent pregnancy
      - Inability to afford tuition and fees
      - Inability to afford school materials
      - Lack of adequate food
    - Added under Types of Studies Difference-in-Differences as a method of analysis that would allow a study to be eligible for inclusion.
    - Added under Types of participants that we would include studies that interact treatment assignment with gender.
  - Data collection and analysis
    - Under Criteria for determination of independent findings, we edited our criteria to specify that we would prioritize the longest term follow-up in cases where the publication date of the earliest published article and longest-term follow-up conflicted.
    - We altered our criteria for multiple intervention groups under Unit of Analysis Issues to report the results of each relevant arm from multi-arm studies separately instead of combining the results of multiple arms into one effect size.
    - Under Meta-analysis of Primary Outcomes, we added or provided more detail to criteria to determine what would be included as a part of potential meta-analyses. We included that studies had to use the same general type of methodology (experimental or quasi-experimental), that measurement of the reported outcome must be the same across all studies, and that a minimum of 3 studies were needed in order to run a meta-analysis.
    - We excluded the secondary outcomes from our analyses due to our assessment that the limited findings may present an incomplete, possibly even biased, picture of the effects of these interventions on the secondary outcomes.

## Section D. Model Types and Formulas Used for Conversion to Partial Correlations

***Notes:***

- IV here refers to independent variable, NOT instrumental variable
- DV refers to the dependent variable
- For all two-stage regressions, the second stage regressor is referenced as the IV
- $r$ refers to the partial correlation
- Equation 1.1 was applied to all results with continuous DVs to improve comparability, unless the equation didn’t properly fit the reported models. In those cases, we applied Equations 1.2.1-2.3 based on the bolded criteria below.

**Continuous DV**

- **Linear models with either Continuous or Dichotomous IVs**
  - Equation 1.1:
    - Equations:
      - $t=\frac{B}{{se}_{B}}$, where $t$ refers to the t-statistic
      - $r=\frac{t}{\sqrt{t^{2}+df}}$
    - Data needed:
      - T-statistic ($t$)or Unstandardized Regression Coefficient and Standard Error ($B, {se}_{B}$)
      - Residual Degrees of Freedom (sample size minus the number of predictors) ($df$)

**Dichotomous DV**

- **Logit Models**
- Equation 2.1: Logit models with dichotomous IV and dichotomous DV
  - Equations:
    - $B=log(OR)$
    - $d=B(\frac{\sqrt{3}}{\pi})$ , where $d$ refers to Cohen’s d
    - $r=\frac{d}{\sqrt{4+d^{2}}}$
  - Data needed:
    - Unstandardized Regression Coefficient or Odds Ratio ($B$ or $OR$)
- **Linear models with dichotomous IVs**
- Equation 2.2.1: Linear models with dichotomous IV and dichotomous DV (if control group success proportion is presented)
  - Equations:
    - $a=n_{treat}(p_{control}+B)$
    - $b=n_{treat}(1-(p_{control}+B))$
    - $c=n_{control}*p_{control}$
    - $d=n_{control}(1-p_{control})$
    - $r=\frac{\left( ad \right)-(bc)}{\sqrt{(a+b)(c+d)(a+c)(b+d)}}$
  - Data needed:
    - Unstandardized Regression Coefficient ($B$)
    - Control group sample size ($n_{treat}$)
    - Treatment group sample size ($n_{control}$)
    - Control group success proportion (i.e. mean) of DV ($p_{control}$)
- Equation 2.2.2: Linear models with dichotomous IV and dichotomous DV (if only overall success proportion is presented)
  - Equations:
    - $a=n_{treat}(p+.5B)$
    - $b=n_{treat}(1-(p+.5B))$
    - $c=n_{control}(p-.5B)$
    - $d=n_{control}(1-(p-.5B))$
    - $r=\frac{\left( ad \right)-(bc)}{\sqrt{(a+b)(c+d)(a+c)(b+d)}}$
  - Data needed:
    - Unstandardized Regression Coefficient ($B$)
    - Control group sample size ($n_{control}$)
    - Treatment group sample size ($n_{treat}$)
    - Overall success proportion (i.e. mean) of DV ($p$)
- **Probit Models**
- Imputed 0 if regression coefficient=0, otherwise:
- Equation 2.3: Probit models
  - Equation:
    - $d=\frac{B}{{SD}_{x}}$
    - $r=\frac{d}{\sqrt{r+d^{2}}}$
  - Data needed:
    - Unstandardized Regression Coefficient ($B$)
    - Standard Deviation of IV (either for the entire analytical sample or disaggregated by treatment and control groups) (${SD}_{x}$)

**Standard errors and Confidence Intervals**

- **Standard errors**
  - If only the standard error of the coefficient is available:
    - Equation 3.1:
      - ${se}_{r}=\frac{r*{se}_{B}}{B}$, where ${se}_{r}$ refers to the standard error of the Partial correlation
    - Data needed
      - Unstandardized Regression Coefficient ($B$)
      - Standard Error of the Unstandardized Regression Coefficient (${se}_{B}$)
  - If only the 95% confidence intervals for the coefficient are available:
    - Equation 3.2:
      - ${se}_{B}=\frac{{CI}_{upper}-{CI}_{lower}}{1.96}$, where ${se}_{B}$ refers to the standard error of the unstandardized regression coefficient
      - ${se}_{r}=\frac{r*{se}_{B}}{B}$, where ${se}_{r}$ refers to the standard error of the Partial correlation
    - Data needed
      - Unstandardized Regression Coefficient ($B$)
    - Confidence intervals of the Unstandardized Regression Coefficient (${CI}_{upper}$, ${CI}_{lower}$)
- **Confidence intervals**
  - The equation below can apply to either regression coefficients as well as partial correlations:
    - Equation 3.3
      - $CI=B\pm{se}_{B}\cdot1.96$
