# Supplementary material for: Policies and interventions to remove gender‐related barriers to girls' school participation and learning in low‐ and middle‐income countries: A systematic review of the evidence
Source: Campbell Syst Rev. 2022 Jan 19;18(1):e1207. doi: 10.1002/cl2.1207 (PMC8770660; doi:10.1002/cl2.1207)
Supplement: Supplementary file 5 — Supplementary Information [file CL2-18-e1207-s003.docx]

# Data and analyses

## GRADE Summary 1

## Findings of Effects for Girls: GRADE Certainty in Evidence and Consolidated Summary

| **Barrier** | **Outcome Description** | **Effect Direction and Size Summary** | **Number of Papers and Participants** | **Certainty in Evidence (Grade Low-High)** | **GRADE Ranking Based on:** | **Consolidated Summary** |
| --- | --- | --- | --- | --- | --- | --- |
| *Barrier 1: Lack of support for girls' education* | Grade attainment | Small to medium positive effects, 2/3 significant. Most directly measured finds no effect. | 20276 participants across 2 experimental papers; 17970 participants across 1 quasi-experimental paper | Very low ⊕OOO | Due to only three studies that largely measure effects indirectly; the most directly measured finds no effect. | More research needed due to lack of studies directly measuring intervention components that address this barrier. Of the four studies that provided somewhat more direct evidence (though they did not test this barrier explicitly) effects were heterogenous (mixed results and very low certainty in the evidence). |
|  | Enrollment in primary school | Small to moderate positive effects, 3/5 significant. Most directly measured finds no effect. | 21729 participants across 3 experimental papers; 21971 participants across 2 quasi-experimental papers | Very low ⊕OOO | Due to studies that only measure effects indirectly; the most directly measured finds no effect. |  |
|  | Enrollment in school (primary or secondary) | Small positive effect, significant (1) but indirect evidence. | 19060 participants across 1 experimental papers | Very low ⊕OOO | Only one study with indirect evidence. |  |
|  | Primary school completion | Small positive effect, non-significant (1), indirect evidence. | 4001 participants across 1 quasi-experimental papers | Very low ⊕OOO | Only one study with indirect evidence. |  |
|  | Absenteeism | Medium positive effect, significant (1), indirect evidence. | 13186 participants across 1 experimental papers | Very low ⊕OOO | Only one study with indirect evidence. |  |
|  | Academic Skills (literacy and numeracy) | 30 effects estimated overall, ranging from small and significantly negative (3) to small (4), medium (6), and large (1) significantly positive, with many null effects. Almost all indirect measures of effects. | 21665 participants across 3 experimental papers; 36688 participants across 3 quasi-experimental papers | Very low  ⊕OOO | Studies largely measure effects indirectly; the most directly measured finds no effect. |  |
|  | Cognitive Skills | In the same study, one arm found significant positive medium effect (1), the other arm had null effect (1). Both arms included efforts to increase support for girls' education, indicating it did not drive effects. | 1152 participants across 1 experimental paper | Very low ⊕OOO | Only one study with somewhat direct evidence of effects. |  |
| \| *Barrier 2: Child marriage and adolescent pregnancy* \| \| --- \| | Grade attainment | 2 studies, each w/ 1 effect: both effects significant in hypothesized direction, but small | 21519 participants across 2 experimental papers | Very low ⊕OOO | Very low due to small number of studies and indirectness concerns for both | More research needed due to small number of studies and lack of studies directly measuring intervention components that address this barrier. Moreover, none of the four studies examined whether reductions in child marriage led to effects on education outcomes.  No identified studies included adolescent pregnancy prevention as an explicit part of the intervention. |
|  | Enrolment in primary school | 2 studies, each w/ 1 effect: one study's (medium) effect size significant in hypothesized direction but w/ a wide confidence interval that bordered 0. The other study's effect was not significant | 8425 participants across 2 experimental papers | Very low ⊕OOO | Very low due to small number of studies, indirectness concerns, and inconsistent findings |  |
|  | Enrolment in school (primary or secondary) | 1 study w/ 1 effect: significant effect in hypothesized direction, but effect size is small and CI borders 0. | 19060 participants across 1 experimental paper | Very low ⊕OOO | Very low due to small number of studies, indirectness concerns, and small effect size w/ confidence interval bordering 0 |  |
|  | Re-enrolment in school among dropouts | 1 study w/ 1 effect: significant (medium) effect in hypothesized direction but wide CI | 5966 participants across 1 experimental papers | Very low ⊕OOO | Very low due to small number of studies and indirectness concerns |  |
|  | Absenteeism | 1 study, 2 measured effects: both statistically insignificant | 2459 participants across 1 experimental paper | Very low ⊕OOO | Very low due to small number of studies, indirectness concerns, and null effects |  |
|  | Academic Skills (literacy and numeracy) | 2 studies: only 3 of 11 effects were in beneficial direction and significant (medium effect size). Of remaining 8, 1 was significant not in the expected direction (medium effect size), and 7 were not significant. At study level 1 found at least 1 significant beneficial effect and the other study found no significant effects. | 7248 participants across 2 experimental papers | Very low ⊕OOO | Very low due to small number of studies, indirectness concerns, and inconsistent findings |  |
| *Barrier 3: Lack of information on returns to education for girls/*  *Alternative roles for women* | Grade attainment | 3 studies: 2 studies with one effect each showed significant beneficial effects, though effect sizes were small, and for 1 the CI bordered 0. In 3rd study (with 6 effects) no effect was significant | 22140 participants across 2 experimental papers; 17970 participants across 1 quasi-experimental paper | Very low ⊕OOO | Very low due to small number of studies with indirect evidence, and inconsistent findings | Despite encouraging results in some settings, more research is needed due to heterogenous effects across studies. Five studies directly assessed programs addressing this barrier (often in disparate ways), and all had at least one significant effect, although effects were heterogenous (including effect sizes that were often small and some CIs bordering zero). However, addressing this barrier was effective in some contexts. More research is needed on which approaches might be most effective at improving girls’ education outcomes in different contexts. |
|  | Years of schooling | 1 study (with 1 effect) finding a significant beneficial effect, but small effect size | 5496 participants across 1 quasi-experimental papers | Very low ⊕OOO | Very low due to small number of studies and small effect size |  |
|  | Enrolment in primary school | 6 studies: 5 had 1 measured effect (the other had 3 effects). 5 of 6 studies and 7 of 8 effects (4 small and 3 medium) were significant in hypothesized direction. One study (indirect) did not have a significant effect. (3 studies with direct evidence (5/5 effects) and 2 studies with indirect (2/2) | 16596 participants across 3 experimental papers; NA participants across 3 quasi-experimental papers | Low ⊕⊕OO | Low due to some indirectness concerns and some small effect sizes, including 1 w/ CI bordering 0 |  |
|  | Enrolment in school (primary or secondary) | 4 studies with 1 effect each. 2 studies/effects significant in hypothesized direction with one small (indirect evidence) and one medium (direct evidence) effect size but latter had a very wide CI and both CIs bordered 0. Other 2 studies had no significant effect | 19979 participants across 2 experimental papers; NA participants across 2 quasi-experimental papers | Very low ⊕OOO | Very low due to small number of studies, some indirectness concerns, and inconsistent findings |  |
|  | Primary school completion | 1 study with 1 effect: non-significant | 4001 participants across 1 quasi-experimental paper | Very low ⊕OOO | Very low due to small number of studies and indirectness concerns |  |
|  | Re-enrolment in school among dropouts | 1 study with 1 effect: beneficial significant effect, medium size but wide CI | 5966 participants across 1 experimental paper | Very low ⊕OOO | Very low due to small number of studies and indirectness concerns |  |
|  | Absenteeism | 1 study with 2 effects: both non-significant | 2366 participants across 1 experimental paper | Very low ⊕OOO | Very low due to small number of studies; null effects |  |
|  | Academic Skills (literacy and numeracy) | 5 studies: 4 studies found at least one significant beneficial effect; 8 of 33 effects were significant in hypothesized direction (1 small, 6 medium, and 1 large effect size) but 2 of CIs bordered 0. One of 2 studies that more directly assessed effects of information on return to schooling did not find any significant effects (0/3) and the other had 1 small w/ CI bordering 0 and 1 medium effect size. | 12452 participants across 4 experimental papers; 35940 participants across 2 quasi-experimental papers | Very low ⊕OOO | Low due to small number of studies, indirectness concerns and inconsistent findings |  |
|  | Cognitive Skills | 1 study with 2 effects: 1 significant beneficial effect (medium size), the other not significant | 1152 participants across 1 experimental paper | Very low ⊕OOO | Very low due to small number of studies and indirectness concerns |  |
| *Barrier 4: School-related gender-based violence* | None | None |  | Very low  ⊕OOO | Very low due to lack of studies addressing SRGBV that examine education outcomes | More research needed due to lack of studies addressing this barrier. |
| *Barrier 5: Gender insensitive school environment* | Grade attainment | 1 w/ 1 significant effect (small); and 1 with 5/9 significant effects (small with CI's close to 0) | 49003 participants across 2 quasi-experimental papers | Very low ⊕OOO | Very low due to small number of studies and indirectness concerns | Despite some encouraging results on improving learning, we conclude that more research is needed due to the small number of studies providing direct estimates of effects. Despite some positive effects, multi-component programs (3, each of which found at least one beneficial effect with a mix of small and medium effect sizes) included school construction, which alone may explain beneficial outcomes. Others (6) were focused on provision of female teachers or training in learner-centered pedagogies, (5/6 finding a significant beneficial effect, however, most effects were small with many CIs bordering zero) and while these programs may comprise one component of efforts to build gender-sensitive schools, they are not sufficient by themselves to make a school gender-sensitive. |
|  | Enrolment in primary school | 4 studies each with at least 1 significant effect. 8/13 significant beneficial effects (3 medium, 5 small; 4 of small w/ CIs bordering 0). 3 were multi-component including construction and 1 focused on female teachers (this latter one had 4 small effects (out of 9 measured) with CIs bordering 0) | 13186 participants across 1 experimental papers; 53004 participants across 3 quasi-experimental papers | Very low  ⊕OOO | Very low due to small number of studies, indirectness concerns, and inconsistent findings |  |
|  | Enrolment in secondary school | 1 study with 1 effect: beneficial significant effect, but small size and CI bordering 0 | 6507 participants across 1 quasi-experimental paper | Very low ⊕OOO | Very low due to small number of studies and indirectness concerns |  |
|  | Primary school completion | 1 study with 1 effect: non-significant | 4001 participants across 1 quasi-experimental paper | Very low ⊕OOO | Very low due to small number of studies and indirectness concerns |  |
|  | Absenteeism | 3 studies: 2/3 studies and 3/4 effects significant (medium size) | 13186 participants across 1 experimental paper; 45406 participants across 2 quasi-experimental papers | Very low  ⊕OOO | Very low due to small number of studies, indirectness concerns, and one study had high risk of bias |  |
|  | Academic Skills (literacy and numeracy) | 7 studies: 5/7 studies and 19/30 effects were significant in hypothesized direction (9 small, 8 medium, 2 large) | 26216 participants across 3 experimental papers; 112379 participants across 5 quasi-experimental papers | Moderate  ⊕⊕⊕O | Moderate due to indirectness concerns and some small effect size |  |
| *Barrier 6: Lack of safe spaces and social connections* | Grade attainment | Estimates (7) from two studies were consistently small and close to null. | 4296 participants across 2 experimental papers | Very low ⊕OOO | Both studies provide indirect evidence of the effects of safe spaces as a platform for delivering life skills and other content. | More research needed due to only one study directly measuring the effects of safe spaces, and otherwise mixed evidence. Two other studies directly assessed grouping girls in the classroom, but these differ from the common approach and their effects were mixed (negative to large beneficial). |
|  | Enrolment in primary school | Small positive significant effects from the study providing direct measure of this intervention. Other (indirect) estimates are null. | 9548 participants across 3 experimental papers | Very low ⊕OOO | Two of three studies provide only indirect evidence. |  |
|  | Enrolment in school (primary or secondary) | Small/medium null effects, only indirect evidence from one study. | 919 participants across 1 experimental paper | Very low ⊕OOO | Only one study providing indirect evidence. |  |
|  | Re-enrolment in school among dropouts | Medium significant positive effect from one study. | 5966 participants across 1 experimental paper | Very low ⊕OOO | Only one study providing indirect evidence. |  |
|  | Absenteeism | Small positive significant effects, including from the study providing direct measure of this intervention. Only two studies. | 2366 participants across 1 experimental paper; 1590 participants across 1 quasi-experimental paper | Very low ⊕OOO | Two studies, one of which provides only indirect evidence. |  |
|  | Academic Skills (literacy and numeracy) | From the studies providing direct evidence, effects range from negative to large and significant. | 15365 participants across 5 experimental papers; 1590 participants across 1 quasi-experimental paper | Low ⊕⊕OO | Multiple studies providing direct evidence, but very different approaches, and results vary. |  |
|  | Cognitive Skills | Null (nearly 0) effect in safe space study arm from one study. | 1152 participants across 1 experimental paper | Very low ⊕OOO | Only one study providing indirect evidence. |  |
| *Barrier 7: Lack of teaching materials and supplies* | Enrolment in primary school | 3 studies: 3/3 studies and 4/4 effects significant in hypothesized direction (medium size effects) | 14676 participants across 2 experimental papers; 4001 participants across 1 quasi-experimental paper | Very low ⊕OOO | Very low certainty due to small number of studies and indirectness concerns | More research needed due to lack of studies directly measuring intervention components that address this barrier. |
|  | Primary school completion | 1 study with 1 effect: non-significant | 4001 participants across 1 quasi-experimental paper | Very low ⊕OOO | Very low due to small number of studies and indirectness concerns |  |
|  | Absenteeism | 1 study: 2/2 effects significant in hypothesized direction (medium) | 13186 participants across 1 experimental paper | Very low ⊕OOO | Very low due to small number of studies and indirectness concerns |  |
|  | Academic Skills (literacy and numeracy) | 4 studies: each with at least 1 sig beneficial effect and 18/26 effects significant in hypothesized direction (7 small, 8 medium, 2 large) | 20206 participants across 4 experimental papers | Very low ⊕OOO | Very low certainty due to small number of studies and indirectness concerns, some (5) wide CI's |  |
|  | Cognitive Skills | 1 study with 1 effect: beneficial sig effect (small) | 1152 participants across 1 experimental papers | Very low ⊕OOO | Very low due to small number of studies and indirectness concerns |  |
| *Barrier 8: Insufficient academic support* | Grade attainment | Small positive significant effect, indirect evidence from one study. | 2459 participants across 1 experimental papers | Very low ⊕OOO | Only one study providing indirect evidence. | Programs that provide training or remedial support, often using technology, are effective at improving learning outcomes. Six studies provide direct evidence of the effectiveness of interventions that provide academic support on academic skills. Results are consistently positive and significant, ranging from small to large.  We find insufficient direct evidence as to whether these interventions improve enrolment or attainment for girls. |
|  | Enrolment in primary school | Effects (7) were mixed, with about half significant (small, medium and large), and half null and close to zero. Results from most direct study were null. | 10956 participants across 4 experimental papers; 57249 participants across 2 quasi-experimental papers | Very low ⊕OOO | Multiple studies, most of which provide indirect evidence. One study with direct evidence had null results. |  |
|  | Primary school completion | Small positive null effect, indirect evidence from one study. | 4001 participants across 1 quasi-experimental paper | Very low ⊕OOO | Only one study providing indirect evidence. |  |
|  | Absenteeism | Most (3/4) estimated effects were null, including from the one study directly measuring these interventions. One significant effect was small and positive (on attendance). | 5458 participants across 2 experimental papers; 1590 participants across 1 quasi-experimental paper | Very low ⊕OOO | Multiple studies, most of which provide indirect evidence. One study with direct evidence had null results. |  |
|  | Academic Skills (literacy and numeracy) | Mixed results, but multiple studies measuring direct effects. Of those six studies, all studies reported positive effects, most of which were significant, ranging from small to large. Results from studies measuring these approaches less directly varied, but many were also positive and significant. | 21995 participants across 8 experimental papers; 55586 participants across 3 quasi-experimental papers | Moderate ⊕⊕⊕O | Multiple studies (six) measuring direct effects of interventions designed to improve academic skills, with largely consistent positive and significant effects ranging in size. |  |
|  | Cognitive Skills | Small positive significant effect in intervention arm focused on building academic skills. | 1152 participants across 1 experimental paper | Very low ⊕OOO | Only one study providing direct evidence. |  |
| *Barrier 9: Inadequate sports programs for girls* | Enrolment in primary school | One study with a medium positive significant effect on the outcome, but indirect assessment of the intervention. | 4001 participants across 1 quasi-experimental paper | Very low ⊕OOO | Only one study providing indirect evidence. | More research needed due to lack of studies directly measuring interventions that address this barrier. |
|  | Primary school completion | One study with a small positive significant effect on the outcome, but indirect assessment of the intervention. | 4001 participants across 1 quasi-experimental papers | Very low ⊕OOO | Only one study providing indirect evidence. |  |
| *Barrier 10: Inadequate health and childcare services* | Enrolment in primary school | One study with a medium positive significant effect on the outcome, but indirect assessment of the intervention. | 4001 participants across 1 quasi-experimental paper | Very low ⊕OOO | Only one study providing indirect evidence. | More research needed due to lack of studies directly measuring interventions that address this barrier. |
|  | Primary school completion | One study with a small positive significant effect on the outcome, but indirect assessment of the intervention. | 4001 participants across 1 quasi-experimental paper | Very low ⊕OOO | Only one study providing indirect evidence. |  |
| *Barrier 11: Inadequate life skills* | Grade attainment | 5 studies: 2/5 studies with one significant effect in expected direction and 2/13 effects (both effects were small), rest were not significant | 45104 participants across 5 experimental papers | Low ⊕⊕OO | Low due to some indirectness concerns and inconsistent findings | More research needed due to heterogenous effects; may be effective in some contexts. Four studies directly assessed effects of life skills: while 2 studies provided direct evidence of a beneficial effect, 2 other studies had null effects.  Mixed results and the small number of studies that disentangle the discrete effects of life skills education give us low confidence in the evidence and we find more research is needed on the characteristics of life skills programs that might be effective at improving girls’ education outcomes. |
|  | Enrolment in primary school | 6 studies: 3/6 studies w/ one significant effect in hypothesized direction and 4/10 effects (1 small and 3 medium), rest were not significant | 31296 participants across 5 experimental papers; 4001 participants across 1 quasi-experimental paper | Very low ⊕OOO | Low due to indirectness concerns and inconsistent findings |  |
|  | Enrolment in school (primary or secondary) | 2 studies: 1/2 studies w one significant beneficial effect; 1/2 effects (small), other not significant | 19979 participants across 2 experimental papers | Very low ⊕OOO | Very low due to small number of studies, some indirectness concerns, and inconsistent findings |  |
|  | Primary school completion | 1 study with 1 effect: non-significant | 4001 participants across 1 quasi-experimental paper | Very low ⊕OOO | Very low due to small number of studies and indirectness concerns |  |
|  | Secondary school completion | 1 study with 1 effect: significant in hypothesized direction (medium but wide CI) | 1597 participants across 1 experimental paper | Very low ⊕OOO | Very low due to small number of studies and indirectness concerns |  |
|  | Re-enrolment in school among dropouts | 1 study with 1 effect: significant in hypothesized direction (medium but wide CI) | 5966 participants across 1 experimental paper | Very low ⊕OOO | Very low due to small number of studies and indirectness concerns |  |
|  | Absenteeism | 3 studies: 1/3 studies with one significant effect in hypothesized direction and 1/4 effects (medium); rest were not significant | 4825 participants across 2 experimental papers; 1590 participants across 1 quasi-experimental papers | Very low ⊕OOO | Very low due to small number of studies, some indirectness concerns, and inconsistent findings |  |
|  | Academic Skills (literacy and numeracy) | 7 studies: 4/7 studies with one significant effect in hypothesized direction. 8/34 significant beneficial effects (7 medium, 1 small), 22/34 were not significant, and 4/34 were significant but not in the hypothesized direction (though 3 of those 4 had CI's bordering 0) | 16251 participants across 5 experimental papers; 2338 participants across 2 quasi-experimental papers | Low ⊕⊕OO | Low due to indirectness concerns and inconsistent findings |  |
|  | Cognitive Skills | 1 study with 2 effects: 1 significant beneficial effect (medium size), the other not significant | 1152 participants across 1 experimental paper | Very low ⊕OOO | Very low due to small number of studies |  |
| *Barrier 12: Inadequate menstrual hygiene management* | Enrolment in primary school | Two studies showed different results - one finding significant improvement and one finding null/unexpected negative effects on enrolment. | 335 participants across 1 experimental paper | Very low ⊕OOO | Very low (due to few studies and serious risk of bias due to imprecision and inconsistent findings) | With only four studies that meet our inclusion criteria, we conclude that more research is needed to understand the effects of interventions designed to address inadequate menstrual hygiene management on education outcomes. While three studies directly assessed the effect of MHM interventions and all reported a significant effect in the expected direction for at least one outcome, effect sizes were predominantly small and null, and concerns about risk of bias and attrition were an issue with two of the studies. |
|  | Absenteeism | Effect sizes ranged from small (negative) to null, with all CIs either crossing 0 or coming close to 0. | 1144 participants across 3 experimental papers | Low ⊕⊕OO | Low (due to few studies, imprecision from one study, and a combination of small and null effect sizes) |  |
|  | Academic Skills (literacy and numeracy) | Most effect sizes are null with large confidence intervals; one effect size is small and positive but comes from a study that did not focus directly on this research question. | 533 participants across 2 experimental papers | Low ⊕⊕OO | Low (due to only two studies, and imprecision from one study) |  |
| *Barrier 13: Lack of water and sanitation* | Grade attainment | Small positive significant effect from study that evaluated broader multi-component intervention. | 17970 participants across 1 quasi-experimental paper | Very low ⊕OOO | Only one study providing indirect evidence of effects of WASH interventions. | We found promising evidence that WASH interventions may improve primary school enrolment and attendance for girls. Three studies provide direct evidence: two of three improve enrolment and attendance for girls with statistically significant effects ranging from small to large. Less evidence is available on learning outcomes, with only one study directly measuring effects and finding no significant results. Therefore, more research is needed to understand the circumstances in which WASH interventions are most likely to improve education outcomes for girls. |
|  | Enrolment in primary school | Largely significant effects in the expected direction (reduced dropout), ranging from small to large, for studies measuring direct effects of WASH interventions. Other studies also found largely positive effects. | 39767 participants across 3 quasi-experimental papers; 19557 participants across 3 experimental papers | Low ⊕⊕OO | Two out of five studies provide direct evidence of the effects of WASH interventions for girls; both found support for effects in expected direction. |  |
|  | Primary school completion | Small positive nonsignificant effect from study that evaluated broader multi-component intervention. | 4001 participants across 1 quasi-experimental paper | Very low ⊕OOO | Only one study providing indirect evidence of effects of WASH interventions. |  |
|  | Absenteeism | Of the two studies providing direct evidence of WASH interventions, one found small positive significant effects, and the other found null effects. | 36786 participants across 3 experimental papers | Low ⊕⊕OO | Two out of three studies provide direct evidence of the effects of WASH interventions for girls; one of the two found support for effects in expected direction. |  |
|  | Academic Skills (literacy and numeracy) | One study estimating direct effects found largely null (close to 0) results. The remaining studies only examined the effects of WASH programs indirectly, and the results were mixed. | 53736 participants across 3 quasi-experimental papers; 13521 participants across 2 experimental papers | Very low ⊕OOO | Only one of five studies provided direct evidence of WASH interventions, and found no effects on learning. |  |
| *Barrier 14: Lack of access to school* | Grade attainment | Nearly all of the estimates from these studies indicated a small or medium, positive and statistically significant effect on grade attainment. | 243212 participants across 9 quasi-experimental papers | Very low ⊕OOO | Despite the inclusion of nine studies, none provided direct estimates of the effects of efforts to expand school access on grade attainment. However, all studies reported on multi-component programs with a substantial focus on school construction, among other components. | We find promising evidence that interventions designed to address inadequate school access may improve school enrolment, attainment, and possibly learning for girls. Studies that more directly measured the impact of these programs found significant effects of small and medium size for enrolment and attainment. Learning outcomes had less consistent results. |
|  | Years of schooling | Both studies found a small to medium positive and statistically significant effect of these interventions on years of schooling. | 5529 participants across 2 quasi-experimental papers | Very low ⊕OOO | Both studies provide indirect evidence of multicomponent government programs with a substantial, but not exclusive, focus on expanding access to school. |  |
|  | Enrolment in primary school | Most estimates indicate a small or medium positive and statistically significant effect of the intervention on primary school enrolment, but most reflect multi-component programs. Estimates from the two studies directly measuring these interventions were positive, medium and significant. | 14676 participants across 2 experimental papers; 319192 participants across 5 quasi-experimental papers | Low ⊕⊕OO | Two out of seven studies provide direct evidence of the effects of interventions expanding school access for girls; both found support for effects in expected direction. |  |
|  | Enrolment in secondary school | Most of the estimates indicate a small or medium positive and statistically significant effect of the intervention on secondary school enrolment, but most reflect multi-component programs. Estimates from the one study directly measuring these interventions were small, positive and significant. | 179700 participants across 7 quasi-experimental papers | Very low ⊕OOO | One out of seven studies provide direct evidence of the effects of interventions expanding school access for girls; that study found support for effects in expected direction. |  |
|  | Grade repetition | This study found a small negative and significant effect of the intervention on grade repetition. | 3451 participants across 1 quasi-experimental paper | Very low ⊕OOO | Only one study providing indirect evidence of effects of interventions aiming to improve school access. |  |
|  | Primary school completion | Four out of five estimates were small and positive; three of those were statistically significant. The fifth estimate was small, negative and null. | 494037 participants across 5 quasi-experimental papers | Very low ⊕OOO | All five studies provide only indirect evidence of effects of interventions aiming to improve school access. |  |
|  | Secondary school completion | All four estimates from two studies were positive; two were medium and significant, the other two were small and null. | 145868 participants across 2 quasi-experimental papers | Very low ⊕OOO | Both studies provide only indirect evidence of effects of interventions aiming to improve school access. |  |
|  | Absenteeism | The one included study found a medium positive and significant effect of the intervention on school attendance. | 13186 participants across 1 experimental paper | Very low ⊕OOO | Only one study providing direct evidence of effects of interventions aiming to improve school access. |  |
|  | Academic Skills (literacy and numeracy) | The results on the effects of these interventions on skills were mixed. Focusing on the five included studies that measured these interventions directly, results varied widely from close to zero and null to large and statistically significant. | 331954 participants across 6 quasi-experimental papers; 20161 participants across 3 experimental papers | Low ⊕⊕OO | Five out of eight studies provide direct evidence of the effects of these interventions on learning, but the results varied considerably, making a clear conclusion difficult. |  |
| *Barrier 15: Poor policy/legal environment* | Grade attainment | All estimates were positive and statistically significant, most were medium with one small effect. | 42869 participants across 6 quasi-experimental papers | Very low ⊕OOO | Multiple studies, all of which provide indirect evidence of the effects of interventions designed to improve the policy/legal environment. | More research needed due to heterogenous effects and lack of direct evidence. Overall, when assessing the interventions that also focus on improving access to school, largely through school construction, we find promising evidence of the effects of policies and laws aiming to improve education outcomes for girls. However, when we set aside those policies, the effects of which may be fully explained by school construction, the remaining policies are varied, and the findings inconsistent, and we conclude that more research is needed on which policies might be most effective at improving girls’ education outcomes. |
|  | Years of schooling | One study reported a significant medium positive effect on years of schooling, while the other reported a significant small negative effect on years of schooling. | 2078 participants across 1 quasi-experimental paper | Very low ⊕OOO | Two studies, only one of which provides direct evidence of effects of interventions aiming to improve school access. |  |
|  | Enrolment in primary school | All three estimates were small and null. One was positive while two were close to zero. | 17721 participants across 1 experimental paper; 53248 participants across 1 quasi-experimental paper | Very low ⊕OOO | Two studies, only one of which provides direct evidence of effects of interventions aiming to improve school access. |  |
|  | Enrolment in secondary school | All studies reported small to medium positive effects, most of which were statistically significant. | 52002 participants across 4 quasi-experimental papers | Very low ⊕OOO | Multiple studies, all of which provide indirect evidence of the effects of interventions designed to improve the policy/legal environment. |  |
|  | Primary school completion | One study reported a small positive significant effect, while the other reported a small negative null effect. | 6615 participants across 2 quasi-experimental papers | Very low ⊕OOO | Two studies, neither of which provides direct evidence of effects of interventions aiming to improve school access. |  |
|  | Secondary school completion | Both effects were medium, positive and statistically significant. | 2078 participants across 1 quasi-experimental paper | Very low ⊕OOO | One study, providing only indirect evidence of effects of interventions aiming to improve school access. |  |
|  | Academic Skills (literacy and numeracy) | All effects (from one study) were medium, positive and statistically significant, although some had fairly wide confidence intervals. | 53248 participants across 1 quasi-experimental papers | Very low ⊕OOO | One study, providing only indirect evidence of effects of interventions aiming to improve school access. |  |
| *Barrier 16: Inability to afford tuition and fees* | Grade attainment | 9 studies with 1 measured effect each. All significant in hypothesized direction (4 small, 4 medium, 1 large). Of the direct studies 3/3 had a sig beneficial effect. | 80252 participants across 8 quasi-experimental papers; 2064 participants across 1 experimental papers | Low ⊕⊕OO | Low due to small number of studies and indirectness concerns | The impacts of programs and policies that directly addressed the inability to afford tuition and fees appear effective, especially at improving enrolment. Nine studies directly measured the effects of these programs. Of these 5 out of 7 find at least one significant beneficial effect for enrolment and attainment outcomes, including both large and small effect sizes. For academic skills most studies that directly assess effects find at least one significant beneficial effect, ranging from small (close to null) to medium in size. (Note that we excluded cash transfer interventions, or other types of transfers to the household, so these results apply to transfers directly to schools.) |
|  | Enrolment in primary school | 7 studies with 10 measured effects. 5/7 studies found at least 1 beneficial significant effect; 5/10 effects were sig in hypothesized direction (2 small with CIs bordering 0; 3 large). Rest of effects were not significant. Of direct studies 3/5 found at least 1 beneficial significant effect. | 19245 participants across 4 experimental papers; 25431 participants across 3 quasi-experimental papers | Moderate ⊕⊕⊕O | Moderate due to some indirectness concerns and inconsistent findings |  |
|  | Enrolment in secondary school | 3 studies w/ 1 effect each. 2/3 studies had beneficial significance: 1 effect was large, 1 was medium, and 1 was not significant. Of direct studies 1/1 found at least 1 significant beneficial effect. | 13798 participants across 2 quasi-experimental papers; 2064 participants across 1 experimental paper | Very low ⊕OOO | Very low due to small number of studies, indirectness concerns, and inconsistent findings |  |
|  | Enrolment in school (primary or secondary) | 1 study with 2 effects, both small, significant effects in hypothesized direction. No direct studies. | 2622 participants across 1 quasi-experimental paper | Very low ⊕OOO | Very low due to small number of studies and indirectness concerns |  |
|  | Primary school completion | 2 studies with 1 effect each: 2/2 effects (small) sig in hypothesized direction. Of direct studies, 1/1 found a significant, small beneficial effect (though CI bordered 0) | 17503 participants across 2 quasi-experimental papers | Very low ⊕OOO | Very low due to small number of studies and indirectness concerns |  |
|  | Secondary school completion | 2 studies with 1 effect each: 1 large significant effect in hypothesized direction and 1 not significant. Both these studies provide direct evidence. | 2064 participants across 1 experimental paper; 12966 participants across 1 quasi-experimental paper | Very low ⊕OOO | Very low due to small number of studies |  |
|  | Academic Skills (literacy and numeracy) | 6 studies: 4/6 studies found at least 1 significant beneficial effect. 6/9 effects were sig in hypothesized direction (5 medium and 1 small whose CI bordered 0). Of the direct studies 3/4 found at least one sig effect in hypothesized direction. | 16854 participants across 4 quasi-experimental papers; 2399 participants across 2 experimental papers | Moderate ⊕⊕⊕O | Moderate due to small number of studies, some indirectness concerns and some inconsistent findings |  |
|  | Cognitive Skills | 1 study: 3/3 effects not significant. This study provided direct evidence. | 2064 participants across 1 experimental paper | Very low ⊕OOO | Very low due to small number of studies |  |
| *Barrier 17: Inability to afford school materials* | Grade attainment | 2 studies: 3/3 effects significant in hypothesized direction (all small and 2 had CIs bordering 0). 1/1 direct studies found small significant effects in hypothesized direction. | 19289 participants across 1 experimental paper; 17970 participants across 1 quasi-experimental paper | Very low ⊕OOO | Very low due to small number of studies and some indirectness concerns | While findings suggest that programs that address the cost of school materials, at least as a part of the overall interventions, are promising, we have greater confidence in results for enrolment and attainment outcomes than for academic skills. Four studies measured the effects of providing school materials directly, 2 of 3 studies found a significant effect for at least one attainment or enrolment outcome (small and medium effects). For academic skills, the one direct study found no significant effects. |
|  | Years of schooling | 1 study with 1/1 effect significant in hypothesized direction (small and CI bordered 0) | 3451 participants across 1 quasi-experimental paper | Very low ⊕OOO | Very low due to small number of studies and indirectness concerns |  |
|  | Enrolment in primary school | 9 studies: 8/9 studies found at least 1 significant beneficial effect. 10/12 effects significant in hypothesized direction (5 small, 4 medium, 1 large). Remaining 2 effects were not significant. Of the 2 direct studies 1 found a small significant effect (w/ CI bordering 0) | 43654 participants across 7 experimental papers; 279459 participants across 2 quasi-experimental papers | Low ⊕⊕OO | Low due to indirectness concerns |  |
|  | Enrolment in secondary school | 1 study (direct): 2/3 effects significant in hypothesized direction (medium). Other effect was not significant. | 748 participants across 1 quasi-experimental paper | Very low ⊕OOO | Very low due to small number of studies and indirectness concerns |  |
|  | Enrolment in school (primary or secondary) | 1 study w 1/1 significant effect in hypothesized direction (small) | 2622 participants across 1 quasi-experimental papers | Very low ⊕OOO | Very low due to small number of studies and indirectness concerns |  |
|  | Grade repetition | 1 study w 1/1 significant effect in hypothesized direction (small) | 3451 participants across 1 quasi-experimental paper | Very low ⊕OOO | Very low due to small number of studies and indirectness concerns |  |
|  | Absenteeism | 4 studies with 1 effect each. 1/4 effects sig in expected direction (medium). The rest were not significant. Neither of the 2 studies measuring direct effects found significant effects. | 22857 participants across 4 experimental papers | Very low ⊕OOO | Very low due to small number of studies, indirectness concerns, and inconsistent findings |  |
|  | Academic Skills (literacy and numeracy) | 7 studies: 4/7 studies found at least 1 significant beneficial effect. 11/19 effects significant in hypothesized direction (3 small, 5 medium, and 3 large). The remaining 8 effects were not significant. The one direct study did not find a significant effect. | 21755 participants across 5 experimental papers; 36688 participants across 3 quasi-experimental papers | Very low ⊕OOO | Very low due to indirectness concerns and inconsistent findings |  |
| *Barrier 18: Lack of adequate food* | Grade attainment | The two studies with direct evidence of these interventions reported small positive effects, one of which was significant. | 22493 participants across 2 experimental papers; 17970 participants across 1 quasi-experimental paper | Low ⊕⊕OO | Two out of three studies provide direct evidence of the effects of interventions addressing inadequate food; one of the two found support for effects in expected direction. | Overall, we find evidence that interventions addressing lack of food may be effective at improving school enrolment, attainment, and attendance for girls. The results on learning were mixed. Seven studies measured direct effects of providing food. Six of these assessed girls’ enrolment, attainment, and/or attendance, finding at least one significant effect (effect sizes ranging from small to medium). The results on learning were mixed, including null to medium effects and one large effect size. More research is needed to isolate the effects of these interventions, and to clarify the circumstances in which programs that address inadequate food access are most likely to be effective. |
|  | Enrolment in primary school | Of the four studies providing direct evidence of effects of these interventions, two found small/medium positive significant effects and the other two found a mix of null and positive significant effects. | 5799 participants across 2 experimental papers; 358076 participants across 5 quasi-experimental papers | Moderate ⊕⊕⊕O | Four studies providing direct evidence of the effects of interventions addressing inadequate food, with largely positive and significant effects; some null results. |  |
|  | Enrolment in school (primary or secondary) | This study found small positive significant effects of the intervention on school enrolment for girls. | 19060 participants across 1 experimental paper | Very low ⊕OOO | One study, providing direct evidence of effects of interventions aiming to improve access to food. |  |
|  | Grade repetition | Both studies reported small negative effects on grade repetition, all of which were null. | 4344 participants across 2 experimental papers | Low ⊕⊕OO | Two studies provide direct evidence of the effects of interventions addressing inadequate food. |  |
|  | Absenteeism | All three studies reported small to medium positive effects on attendance. Estimates from studies measuring direct effects were all positive, and mostly significant. | 6710 participants across 3 experimental papers | Low ⊕⊕OO | Two of three studies provide direct evidence of the effects of interventions addressing inadequate food. |  |
|  | Academic Skills (literacy and numeracy) | Of the two studies providing direct evidence, results were mixed and ranged from negative and significant to positive and significant. | 6710 participants across 3 experimental papers; 35940 participants across 2 quasi-experimental papers | Low ⊕⊕OO | Two of four studies provide direct evidence of the effects of interventions addressing inadequate food, but findings vary considerably. |  |
|  | Cognitive Skills | Estimated effects on cognitive skills ranged from null (close to zero) to medium, positive and significant. | 8484 participants across 3 experimental papers | Low ⊕⊕OO | Three studies providing direct evidence of the effects of interventions addressing inadequate food, but findings vary considerably. |  |

*Note that when two papers from the same study were in a single barrier-outcome pairing, both papers were counted, but participants were only counted once

## GRADE Summary 2

## Findings of Effects for Girls and Boys Combined, and Gender Differential Effects: GRADE Certainty in Evidence and Consolidated Summary

| **Barrier** | **Outcome Description** | **Effect Direction and Size Summary** | **Number of Papers and Participants** | **Certainty in Evidence (Grade Low-High)** | **GRADE Ranking Based on:** | **Consolidated Summary** |
| --- | --- | --- | --- | --- | --- | --- |
| ***Barrier 1: Lack of support for girls' education*** | Grade attainment | Small positive significant effect overall (1), and significantly larger for girls, but indirect measure of effect of this component. | 17970 participants across 1 quasi-experimental paper | Very low ⊕OOO | Only one study providing indirect evidence of effects of this component. | More research needed due to a lack of direct evidence. |
|  | Enrolment in primary school | Small significant effects for girls and boys combined (2), one of which is significantly larger for girls. Mostly indirect evidence. | 7327 participants across 1 experimental paper; 17970 participants across 1 quasi-experimental paper | Very low ⊕OOO | Only two studies, both of which are largely indirect evidence of effects of this component. |  |
|  | Academic Skills (literacy and numeracy) | Estimates range from small to large, some null and some significant. Some larger for girls. Most evidence is indirect. | 7327 participants across 1 experimental paper; 35940 participants across 2 quasi-experimental papers | Very low ⊕OOO | Wide-ranging estimates from only two studies, with largely indirect evidence. |  |
| ***Barrier 3: Lack of information on returns to education for girls/***  ***Alternative roles for women*** | Grade attainment | 1 study w/ 1 effect: small significant beneficial effect for girls and boys combined with greater effect for girls. | 17970 participants across 1 quasi-experimental paper | Very low ⊕OOO | Very low due to small number of studies and indirectness concerns | More research needed due to a lack of direct evidence (only one study provided direct evidence). |
|  | Years of schooling | 1 study w/ 1 effect: small significant beneficial effect for girls and boys combined with greater effect for girls. | 5496 participants across 1 quasi-experimental paper | Very low ⊕OOO | Very low due to small number of studies |  |
|  | Enrolment in primary school | 1 study w/ 1 effect: small significant beneficial effect for girls and boys combined with greater effect for girls. | 17970 participants across 1 quasi-experimental paper | Very low ⊕OOO | Very low due to small number of studies and indirectness concerns |  |
|  | Enrolment in school (primary or secondary) | 1 study w/ 1 effect: not significant for girls and boys combined; no gender difference | 5496 participants across 1 quasi-experimental paper | Very low ⊕OOO | Very low due to small number of studies |  |
|  | Academic Skills (literacy and numeracy) | 1 study (2 papers) w/ 3 effects: 3/3 significant beneficial effects for girls and boys combined (1 medium, 2 large) and 1 of the effects was greater for girls (no gender difference for other 2) | 35940 participants across 2 quasi-experimental papers | Very low ⊕OOO | Very low due to small number of studies and indirectness concerns |  |
| ***Barrier 5: Gender insensitive school environment*** | Grade attainment | 1 study w/ 1 effect: small significant beneficial effect for girls and boys combined with greater effect for girls. | 17970 participants across 1 quasi-experimental paper | Very low ⊕OOO | Very low due to small number of studies and indirectness concerns | More research needed due to heterogenous effects. Four single component programs (one of which found a greater effect for girls, the others did not) and one multi-component which included school construction (found a greater effect for girls than for boys for 3 outcomes). |
|  | Enrolment in primary school | 1 study w/ 1 effect: small significant beneficial effect for girls and boys combined with greater effect for girls. | 17970 participants across 1 quasi-experimental paper | Very low ⊕OOO | Very low due to small number of studies and indirectness concerns |  |
|  | Absenteeism | 1 study w/ 1 effect: small significant beneficial effect for girls and boys combined (CI borders 0); no significant difference between girls and boys | 43816 participants across 1 quasi-experimental paper | Very low ⊕OOO | Very low due to small number of studies and some indirectness concerns |  |
|  | Academic Skills (literacy and numeracy) | 5 studies w/ 15 effects: 4/15 significant beneficial effects for girls and boys combined (2 medium, 2 large); the 2 medium effects were significantly greater for girls than boys; no gender differences for the 2 large effects | 13030 participants across 2 experimental papers; 110789 participants across 4 quasi-experimental papers | Very low ⊕OOO | Very low due to indirectness concerns and inconsistent findings |  |
| ***Barrier 7: Lack of teaching materials and supplies*** | Enrolment in primary school | 1 study w/ 1 effect: medium significant beneficial effect for girls and boys combined with greater effect for girls. | 1490 participants across 1 experimental paper | Very low ⊕OOO | Very low due to small number of studies and indirectness concerns | More research needed due to a lack of direct evidence. |
|  | Academic Skills (literacy and numeracy) | 1 study w/ 1 effect: large sig beneficial effect (but wide CI) for girls and boys combined with greater effect for girls. | 1490 participants across 1 experimental paper | Very low ⊕OOO | Very low due to small number of studies and indirectness concerns |  |
| ***Barrier 8: Insufficient academic support*** | Enrolment in primary school | Small positive significant effect for girls and boys combined, no difference by sex, one study measuring direct effects of intervention. | 7327 participants across 1 experimental paper | Very low ⊕OOO | Only one study providing direct evidence of effects of this component. | More research needed due to heterogenous effects. Four studies provide fairly direct evidence, but none show greater effects for girls than boys. |
|  | Absenteeism | Medium negative nonsignificant effect on absenteeism for girls and boys combined, no difference by sex, one study measuring direct effects of intervention. | 2999 participants across 1 experimental paper | Very low ⊕OOO | Only one study providing direct evidence of effects of this component. |  |
|  | Academic Skills (literacy and numeracy) | Most (3/4) studies reported one or more significant positive effects on skills for girls and boys combined, ranging from small to large. None found differences in effects by sex. | 13671 participants across 4 experimental papers | Low ⊕⊕OO | Several studies providing direct evidence of the effects of this component, variation in magnitude of estimates. |  |
| ***Barrier 11: Inadequate life skills*** | Academic Skills (literacy and numeracy) | 1 study w/ 2 effects: 1 effect was large and in hypothesized direction for girls and boys combined, no gender differences. 1 effect was not significant | 5485 participants across 1 experimental paper | Very low ⊕OOO | Very low due to small number of studies and indirectness concerns | More research needed due to a lack of direct evidence. |
| ***Barrier 13: Lack of water and sanitation*** | Grade attainment | Small positive significant effect for girls and boys, significantly larger effect for girls, from one study that evaluated broader multi-component intervention (not focused on WASH). | 17970 participants across 1 quasi-experimental paper | Very low ⊕OOO | Only one study providing indirect evidence of effects of this component. | More research needed due to a lack of direct evidence (only 1 study provided direct evidence). |
|  | Enrolment in primary school | One study providing direct evidence found significant negative effects on dropout, which is larger among older students, but no difference by sex. Study reported indirect evidence found small positive effect for girls and boys combined, larger for girls. | 35766 participants across 2 quasi-experimental papers | Very low ⊕OOO | Two studies, only one of which provided direct evidence of effects of this component. |  |
|  | Academic Skills (literacy and numeracy) | Study assessing effects of larger multi-component intervention found large positive and significant effects on skills overall, with no difference by sex. | 35940 participants across 2 quasi-experimental papers | Very low ⊕OOO | Only one study (two papers) providing indirect evidence of effects of this component. |  |
| ***Barrier 14: Lack of access to school*** | Grade attainment | The authors of the one included study report a small to medium positive effect of the intervention on grade attainment for boys and girls combined, an effect that is significantly larger for girls. | 17970 participants across 1 quasi-experimental paper | Very low ⊕OOO | Only one study providing indirect evidence of the effects of this component. | More research is needed due to lack of direct evidence. |
|  | Enrolment in primary school | The two studies both report positive and significant effects, one small and one medium, on primary school enrolment. In both cases the effects were significantly larger for girls than boys. | 1490 participants across 1 experimental papers; 17970 participants across 1 quasi-experimental paper | Very low ⊕OOO | Two studies, only one of which provides direct evidence of the effects of this component. |  |
|  | Academic Skills (literacy and numeracy) | Of the three studies providing direct evidence, all three reported medium to large positive effects on at least one skill. | 6975 participants across 2 experimental papers; 97268 participants across 4 quasi-experimental papers | Low ⊕⊕OO | Several studies providing direct evidence of the effects of this component, variation in magnitude of estimates. |  |
| ***Barrier 15: Poor policy/legal environment*** | Years of schooling | The author reported a medium positive significant effect of the intervention on years of schooling for girls and boys combined, with no difference by sex. | 201656 participants across 1 quasi-experimental paper | Very low ⊕OOO | Only one study providing direct evidence of the effects of this component. | More research needed due to lack of direct evidence. |
|  | Enrolment in primary school | The authors reported a medium positive significant effect on primary school enrolment for boys and girls combined, with no difference by sex. | 17721 participants across 1 experimental paper | Very low ⊕OOO | Only one study providing direct evidence of the effects of this component. |  |
| ***Barrier 16: Inability to afford tuition and fees*** | Enrolment in primary school | 1 study w/ 2 effects: 2 significant beneficial effects (medium) for girls and boys combined; no gender differences | 17721 participants across 1 experimental paper | Very low ⊕OOO | Very low due to small number of studies | More research needed due to a lack of direct evidence. |
|  | Enrolment in school (primary or secondary) | 1 study w/ 2 effects: neither effect was significant; no gender differences | 2622 participants across 1 quasi-experimental paper | Very low ⊕OOO | Very low due to small number of studies and indirectness concerns |  |
|  | Academic Skills (literacy and numeracy) | 2 studies w/ 4 effects: all non-significant; no gender differences | 1104 participants across 1 quasi-experimental papers; 18581 participants across 1 experimental paper | Very low ⊕OOO | Very low due to small number of studies and some indirectness concerns |  |
| ***Barrier 17: Inability to afford school materials*** | Grade attainment | 1 study w/ 1 effect: small significant beneficial effect for girls and boys combined, with greater effect for girls. | 17970 participants across 1 quasi-experimental paper | Very low ⊕OOO | Very low due to small number of studies and indirectness concerns | More research needed due to a lack of direct evidence. |
|  | Enrolment in primary school | 2 studies with 2 effects: 2 significant beneficial effects (small and medium) for girls and boys combined, with greater effects for girls | 1490 participants across 1 experimental papers; 17970 participants across 1 quasi-experimental paper | Very low ⊕OOO | Very low due to small number of studies and indirectness concerns |  |
|  | Enrolment in school (primary or secondary) | 1 study w/ 1 effect: not significant for girls and boys combined; no gender difference | 2622 participants across 1 quasi-experimental paper | Very low ⊕OOO | Very low due to small number of studies and indirectness concerns |  |
|  | Absenteeism | 2 studies w/ 2 effects: 1 small significant beneficial effect for girls and boys combined and 1 effect not significant; neither had a gender difference | 7305 participants across 2 experimental papers | Very low ⊕OOO | Very low due to small number of studies and inconsistent effects |  |
|  | Academic Skills (literacy and numeracy) | 3 studies w/ 6 effects: all 6 effects significant in hypothesized direction for girls and boys combined (3 large, 3 medium); for 2 of 6 effects were greater for girls than boys | 1490 participants across 1 experimental paper; 39391 participants across 3 quasi-experimental papers | Very low ⊕OOO | Very low due to small number of studies and indirectness concerns |  |
| ***Barrier 18: Lack of adequate food*** | Grade attainment | This study reported a small positive significant effect on grade attainment for girls and boys combined, which was significantly larger for girls. | 17970 participants across 1 quasi-experimental paper | Very low ⊕OOO | Only one study providing indirect evidence of the effects of this component. | More research needed due to a lack of direct evidence. |
|  | Enrolment in primary school | Both studies reported small positive significant effects on primary school enrolment, in all cases significantly larger for girls than boys. | 35583 participants across 2 quasi-experimental papers | Very low ⊕OOO | Two studies, one of which provides indirect evidence of the effects of this component. |  |
|  | Academic Skills (literacy and numeracy) | Both papers (from the same study) reported medium to large positive significant effects on academic skills for boys and girls combined. | 35940 participants across 2 quasi-experimental papers | Very low ⊕OOO | Only one study providing indirect evidence of the effects of this component. |  |

Note: Does not include Chatterjee (2017) and Jacoby and Mansuri (2011) due to lack of reporting of the overall effects of their evaluated programs on both boys and girls.

*When two papers from the same study were in a single barrier-outcome pairing, both papers were counted, but participants were only counted once.
